# Stress-Adaptive Responses Associated with High-Level Carbapenem Resistance in KPC-Producing* Klebsiella pneumoniae*

**DOI:** 10.1155/2018/3028290

**Published:** 2018-03-19

**Authors:** Sheila Adams-Sapper, Adam Gayoso, Lee. W. Riley

**Affiliations:** ^1^School of Public Health, Division of Infectious Diseases and Vaccinology, University of California, Berkeley, Berkeley, CA, USA; ^2^Department of Computer Science, Columbia University, 500 W 120th Street, New York, NY 10027, USA

## Abstract

Carbapenem-resistant Enterobacteriaceae (CRE) organisms have emerged to become a major global public health threat among antimicrobial resistant bacterial human pathogens. Little is known about how CREs emerge. One characteristic phenotype of CREs is heteroresistance, which is clinically associated with treatment failure in patients given a carbapenem. Through* in vitro* whole-transcriptome analysis we tracked gene expression over time in two different strains (BR7, BR21) of heteroresistant KPC-producing* Klebsiella pneumoniae,* first exposed to a bactericidal concentration of imipenem followed by growth in drug-free medium. In both strains, the immediate response was dominated by a shift in expression of genes involved in glycolysis toward those involved in catabolic pathways. This response was followed by global dampening of transcriptional changes involving protein translation, folding and transport, and decreased expression of genes encoding critical junctures of lipopolysaccharide biosynthesis. The emerged high-level carbapenem-resistant BR21 subpopulation had a prophage (*IS*1) disrupting* ompK36* associated with irreversible OmpK36 porin loss. On the other hand, OmpK36 loss in BR7 was reversible. The acquisition of high-level carbapenem resistance by the two heteroresistant strains was associated with distinct and shared stepwise transcriptional programs. Carbapenem heteroresistance may emerge from the most adaptive subpopulation among a population of cells undergoing a complex set of stress-adaptive responses.

## 1. Introduction

In 2013, the Centers for Disease Control and Prevention (CDC) designated carbapenem-resistant Enterobacteriaceae (CRE) as “urgent threat” pathogens [1]. In early 2017 the World Health Organization (WHO) included CREs among “Priority 1, critical” pathogens among a global priority list of antibiotic-resistant bacteria (http://www.who.int/mediacentre/news/releases/2017/bacteria-antibiotics-needed/en/). CREs are considered high-consequence antibiotic threats because infections caused by them are desperately in need of new treatments [1–3]. Among the most worrisome CREs are the Gram-negative bacilli that produce* Klebsiella pneumoniae* carbapenemase (KPC), a broad-spectrum *β*-lactamase. KPC inactivates carbapenems as well as all other *β*-lactam drugs.* Klebsiella pneumoniae*, a common cause of infections associated with health-care settings, is the most frequently identified KPC-producer [4]. The *bla*_KPC_ gene that encodes the enzyme is carried on several types of plasmids that are readily transmitted to other Gram-negative species such as* Escherichia coli*, one of the most important causes of community-onset infections [4, 5].

KPC-producing strains are sometimes missed by routine automated-device susceptibility tests [6–10], which are often suggested to result from heterogeneous subpopulations among these strains [7, 11–14]. Such subpopulations can adapt to changing environments [7, 15–18]. These subpopulations not only complicate their detection but also may acquire* in vivo* high-level resistance that leads to treatment failure and increased mortality [6, 8–10, 19–25]. Indeed, mortality associated with KPC-producing* K. pneumoniae* (KPC-Kp) infections in many hospitals exceeds 50% [6, 20, 23, 25]. The efficacious treatment of KPC-Kp infections remains very challenging [6, 8, 20, 23, 25, 26].

We previously showed that exposure of carbapenem-heteroresistant KPC-Kp strains to a bactericidal concentration of imipenem resulted in a reproducible, biphasic pattern of near-complete killing of the population, followed by recovery within 20 hours (h) [11]. The minimum inhibitory concentration (MIC) of imipenem for the recovered population increased at least fourfold compared to the MIC for the population before imipenem exposure. This high-level resistance characteristically occurred through loss of the OmpK36 porin that facilitates entry of imipenem.

Here, we undertook this study to understand how subpopulations of heteroresistant KPC-Kp survive after exposure to a bactericidal concentration of a carbapenem. We identified sequential complex stress-related transcriptional changes that these KPC-Kp strains undergo, which were associated with selection of high-level carbapenem-resistant bacterial cell populations. Furthermore, this high-level resistance appeared to involve mechanisms beyond drug inactivation by KPC-mediated hydrolysis or drug exclusion by porin modification.

## 2. Results and Discussion

### 2.1. Few Genetic Mutations Observed in Two Clonally Related Carbapenem-Heteroresistant Strains after Lethal Imipenem Exposure

We compared two clinical strains of KPC-Kp (BR7 and BR21) that are carbapenem-heteroresistant and carry *bla*_KPC_; they belong to multilocus sequence type ST437—members of the ST258 clonal complex most commonly distributed worldwide [27–30]. The imipenem heteroresistant behavior of both strains was previously characterized [11]. They are 99.9% similar at the genome sequence level, excluding one 70 kb prophage carried by BR21 (Tables S1 and S2). We also analyzed the whole genomes of the 2 h and 8 h imipenem-exposed samples of each strain to look for mutations associated with lethal-dose imipenem exposure (Table S2). We compared these strains because of our previous observation that OmpK36 porin synthesis differed between them [11]. While neither strain produced the porin after 8 h of lethal imipenem exposure, strain BR7 resumed production of the porin after multiple passages in drug-free media (reverting to wild-type imipenem susceptibility), while strain BR21 did not. The only major difference in the chromosomal sequences of imipenem-exposed samples compared to their unexposed counterparts was that due to low quality assembly and base-calling errors, primarily within and around mobile genetic elements. One exception was an IS*I* interruption found in the coding region of* ompK36* in 8 h-exposed samples of BR21, but not in unexposed or 2 h-exposed samples (Table S2). The element was 100% identical to a chromosomal phage-encoded IS*1* from SfIV (NCBI number NC022749) located in a P4 prophage region interrupted by genes from other phage sources (Table S1).

### 2.2. A Diverse Transcriptional Response Is Observed in Two Clonally Related Carbapenem-Heteroresistant Strains after Lethal Imipenem Exposure Followed by Growth in Drug-Free Medium

**Figure 1:**
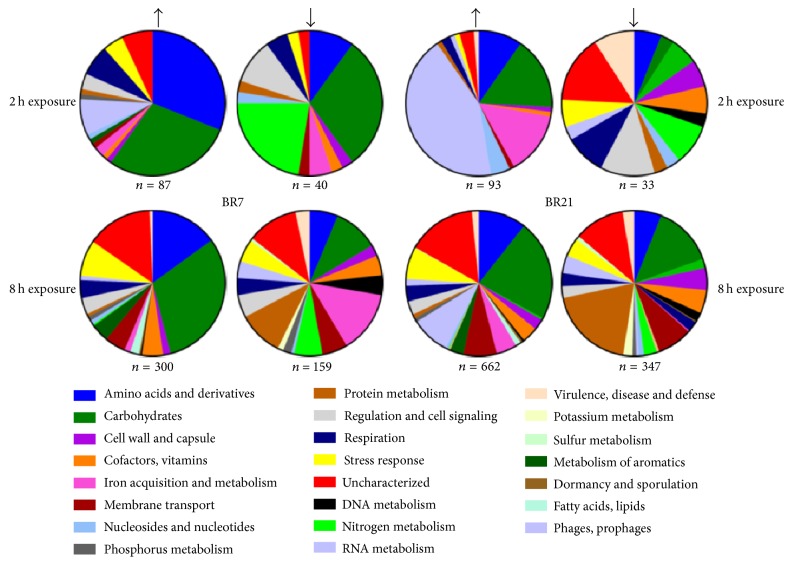
*Differential expression after 2 h and 8 h of lethal imipenem exposure*. The proportion (%) of genes by ontological category showing increased or decreased expression (indicated by black arrows) after 2 h or 8 h of imipenem exposure for strains BR7 and BR21 (shown here with minimum threshold of 2-fold differential expression of imipenem-exposed compared to unexposed). Note that the proportion of stress-response genes is limited to those identified through initial annotation (see text for details).

Despite their genomic and phenotypic similarity, we observed some striking transcriptome-level differences between the two strains after exposure to imipenem followed by incubation in drug-free medium. While both had relatively few differentially expressed genes after 2 h of imipenem exposure (Figure 1), BR21 had more than twice the number (1009) of differentially expressed genes compared to BR7 (459) after 8 h of exposure. Both showed strong expression of genes for carbohydrate metabolism after 2 h of exposure (Figure 1). BR7 also showed increased expression of genes involved in amino acid metabolism and transport, while BR21 showed increased expression of genes involved in iron acquisition and metabolism and in prophage genes. In general, if differential expression was observed for a shared gene in both strains, the direction (up or down) was in agreement (Table S3).

**Figure 2:**
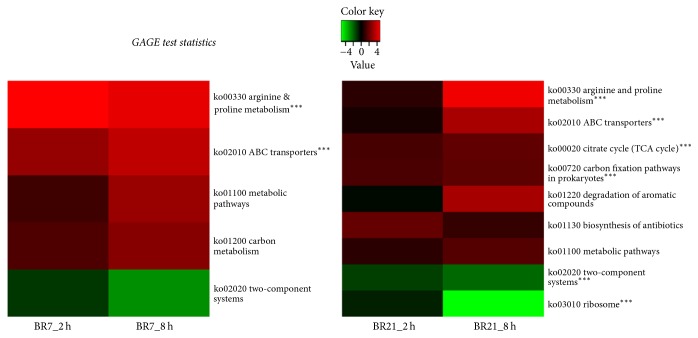
Significantly enriched KEGG orthology (KO) pathways after 2 h and 8 h of imipenem exposure followed by growth in drug-free broth medium. Generally applicable gene-set enrichment (GAGE) analysis shows log_2_ fold change (FC) ratios from low (green) to high (red) expression in significantly enriched KO pathways for imipenem-exposed compared to unexposed bacterial populations (three biological replicates in each sample group). A false discovery rate *q*-value cutoff ≤ 0.2 was applied. ^*∗∗∗*^KO pathways with *q*-value of <0.1.

Gene Set Analysis (GSA) showed significant expression of genes in five and nine KEGG orthology (KO) pathways in BR7 and BR21, respectively (Figure 2). BR7 had only one differentially expressed plasmid-encoded gene while BR21 had 14 differentially expressed genes among its two plasmids which encoded conjugative transfer, restriction modification, DNA repair, and iron transport functions. The plasmid-mediated *bla*_KPC_ gene was 100% identical in sequence in both strains and was not differentially expressed after imipenem exposure.

### 2.3. Lethal Imipenem Exposure Is Associated with Differential Expression of Genes Encoding Metabolic Regulatory Pathways

**Figure 3:**
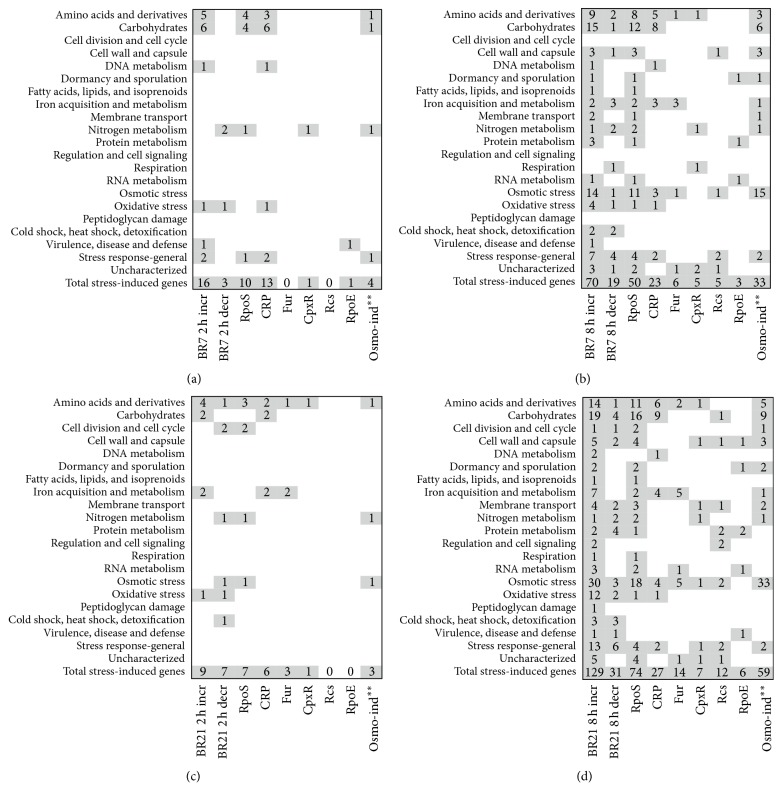
*Transcriptional changes associated with strong osmotic and general RpoS stress response after lethal imipenem exposure*. The panels indicate the number of genes (by ontological category) associated with stress-response regulons RpoS, CRP, Fur, CpxR, Rcs, and RpoE for BR7 2 h (a), 8 h (b), BR21 2 h (c), and BR21 8 h (d). The first two columns in each panel indicate total stress-associated genes with increased (incr, column 1) or decreased (decr, column 2) expression (shown here with minimum threshold of 2-fold differential expression of imipenem-exposed compared to unexposed). Genes associated with stress regulons (in addition to those shown in Figure 1) were identified by RegulonDB, UniProt, and the experimental work of others. ^*∗∗*^Genes associated with osmotic stress were identified through the experimental work cited here [31]. Table S4 presents the data by individual differentially expressed genes.

Ontological annotation categorized 77 differentially expressed genes as those involved in adaptions to osmotic and oxidative stress, membrane and cell wall damage, and general stress responses. We identified 90 additional differentially expressed genes associated with stress regulons based on a search of RegulonDB, UniProt, and the work of others (Table S4, Figure 3) [31, 34, 35, 37–58]. These genes are involved in diverse categories of amino acid, carbohydrate, and protein metabolism, as well as cell wall biosynthesis and transport. Osmotic stress-associated genes had greater than 2-fold change in expression in 33 (36%) of 89 and 59 (36%) of 160 genes associated with stress response in imipenem-exposed BR7 and BR21, respectively, compared to drug unexposed strains (Figure 3). The strongest association was observed at 8 h of imipenem exposure in both strains.

**Figure 4:**
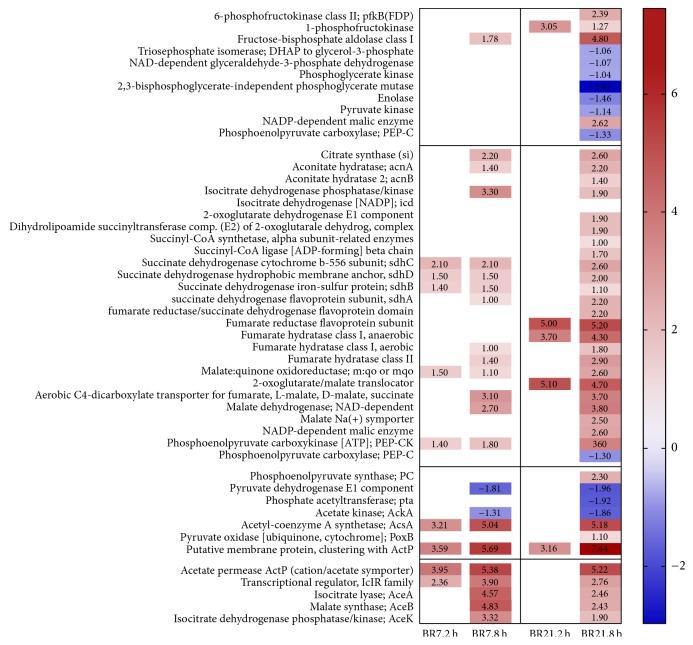
*Transcriptional changes in glycolytic pathway: upper glycolysis (section 1, lines 1–11), lower glycolysis: pyruvate and acetate (section 3, lines 38–44), the tricarboxylic acid (TCA) pathway (section 2, lines 12–37), and the glyoxylate shunt (section 4, lines 45–48) after lethal imipenem exposure*. At 8 h of imipenem exposure, both BR7 and BR21 strains exhibit a similar metabolic transcriptional profile. Illustrations to guide the reader through these pathways are provided in cited research [32, 33]. Color scale shows gene expression of log_2_ fold change (FC) from low (blue) to high (red) expression in imipenem-exposed compared to unexposed bacterial populations (three biological replicates in each sample group). A false discovery rate *p* value correction (FDR) ≤ 0.05 was applied.

The genes encoding enzymes in glycolysis were either not differentially expressed (BR7) or were downregulated (BR21), with the exception of the earliest genes in the pathway (Figure 4). As a result, pyruvate generated from glycolysis is unlikely to serve as a primary source of acetyl-CoA needed to enter the TCA cycle. GSA indicated a significant enrichment of genes in the TCA cycle in BR21 (Figure 2, Table S3). The genes encoding the initial enzymes for the TCA cycle from citrate synthase (the formation of citrate from acetyl-CoA and oxaloacetate) and the reversible conversion of citrate to isocitrate showed increased expression in 8 h-exposed samples of both strains (Figure 4). Expression of the genes of the glyoxylate bypass cycle, catalyzed by the* aceBAK* operon to mediate growth on acetate or fatty acids in the absence of glucose, was strongly increased in both BR7 and BR21 [32, 33, 49, 54, 59]. The 8 h-exposed samples of both strains also had reduced expression of the genes encoding the pyruvate dehydrogenase complex (Figure 4). Increased expression of genes encoding conversion of pyruvate to acetate (pyruvate oxidase) was observed in 8 h-exposed samples of BR21. Genes encoding conversion of pyruvate to acetaldehyde (pyruvate decarboxylase) were upregulated in 8 h-exposed samples of both strains. These observations are consistent with a metabolic shift from the TCA to the glyoxylate shunt pathway [49, 59].

**Figure 5:**
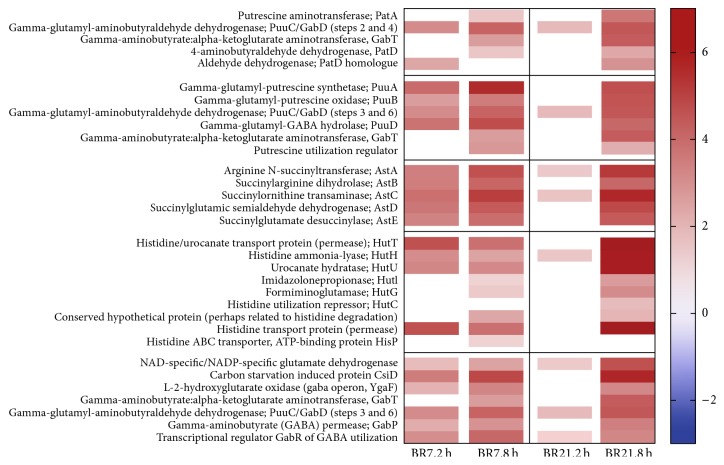
*Transcriptional response after lethal imipenem exposure is associated with key metabolites entering the TCA pathway via increased expression of glutamate/γ-aminobutyrate (GABA) catabolic pathways*: transaminase pathway of putrescine degradation (section 1, lines 1–5), glutamylated putrescine pathway of putrescine degradation (section 2, lines 6–11), arginine degradation pathway to glutamate (section 3, lines 12–16), histidine degradation pathway to glutamate (section 4, lines 17–25), and glutamate flux to TCA, including the GABA shunt operon (section 5, lines 26–32). Illustrations to guide the reader through these pathways are provided in cited research [34–36].

Numerous genes involved in glutamate metabolism showed strong enhanced expression after imipenem exposure (Figure 5). Glutamate is known to be a predominant source of carbon and nitrogen, especially in osmotically stressed cells [34, 35, 43, 60–62]. The genes of the ATP-binding cassette (ABC) glutamate-aspartate transporter* gltIJKL* had increased expression in all imipenem-exposed samples of BR7 and in 8 h-exposed samples of BR21. These findings were confirmed by GSA for both strains (Table S3). The* glt*-encoded glutamate synthesis genes (from *α*-ketoglutarate and asparagine) were not differentially expressed. Instead, we saw high-level expression of genes in four different catabolic pathways, which may result in glutamate and GABA production as by-products or end-products (Figure 5). In fact, six of the nine genes with increased expression in both strains at 2 h and 8 h of exposure were found in these pathways.

We also observed increased expression in all genes of the transaminase and glutamylated putrescine catabolic pathways (Figure 5). GSA supported the findings of significant enrichment of genes in these pathways (Figure 2, Table S3). The GABA shunt, an important part of these pathways, feeds succinate and NAD(P)H formed in the putrescine degradation pathways into the TCA cycle [34, 43]. These genes belong to the well-characterized carbon starvation-induced operon,* csiD*-*ygaF*-*gabDTP* [31, 34, 35].

All of the genes in the arginine and histidine catabolic pathways leading to the formation of glutamate were strongly expressed (Figure 5). GSA identified significant enrichment of the genes in the arginine catabolic pathway (Figure 2, Table S3). The first gene in this pathway is reported to be RpoS-controlled [35].

GABA and glutamate are among the first compatible solutes that rapidly accumulate during osmotic stress and two of the most predominant compatible solutes in bacteria [43, 61, 62]. Glutamate also induces the uptake of other important osmoprotectants. Accordingly, we observed increased expression of genes encoding transport for the osmolytes glycine betaine and the CRP-regulated* osmC*,* osmY*, and the proline transporter,* putP* (Table S4).

### 2.4. Imipenem Exposure Is Associated with Differential Expression of Genes Involved in Outer Membrane Protein Integrity, Transport, and Processing, as well as in Global Dampening of Protein Expression

We previously reported that the observed loss of OmpK36 in 8 h-exposed samples of both BR7 and BR21 was a key factor in the conversion from relative imipenem susceptibility to high-level imipenem resistance [11]. Mutants with loss of the carbapenem-heteroresistant phenotype did not acquire high levels of imipenem resistance and did not abolish production of this porin [63]. We found no differential expression of* ompK36* in 2 h-exposed B21 samples, nor in any imipenem-exposed BR7 samples (Table S3). Expression of* ompK35* did not differ with imipenem exposure. In fact,* ompK35* had very low expression even in unexposed samples, which agrees with our former report of its absence in SDS-PAGE analysis [11].

Small RNAs are known to control the synthesis of outer membrane porins [45, 64, 65]. We found no differential expression of* micF (ompK35)*,* micC*, or* rybB (ompK36)*. Other small noncoding RNAs have been reported to control the translation of* ompC* in* E. coli* [45, 66], but we did not identify similar sequences in our* K. pneumoniae* strains. However, in both strains we found significant differential expression of* micA*, a small RNA reported to be under the control of RpoE in response to accumulation of misfolded proteins, which decreases the stability of the* ompA* transcript in* E. coli* (Figure S1) [45].

We next examined the expression of genes involved in protein processing and export from the cytoplasm to the outer membrane. Surprisingly, while BR7 only showed increased expression of the* clpA* heat shock and* hflc*-like protease genes in 8 h-exposed samples, BR21 showed differential expression in numerous genes associated with these functions (Figure S1). These include decreased expression in 8 h-exposed samples of the* sec*-encoded genes,* secA* and* secY*. The Sec-dependent pathway is important for export of a majority of proteins, including the *β*-barrel outer membrane porins [37]. Expression of the integral membrane universal stress protein B* (uspB)* was increased in 8 h-exposed samples of both strains. This protein is induced by RpoS and carbon starvation and has been implicated in sensing and responding to membrane damage [42]. The RpoS-regulated, osmotically induced protein* osmB*, described as having a role in membrane resealing, [40] had increased expression only in 8 h-exposed samples of BR21.

We also found decreased expression of genes encoding ribosomal proteins and translation initiating factors in 8 h-exposed samples of both strains, indicating a global dampening of protein translation (Figure S1). This may be an additional effect of the stress response imposed by the antibiotic, involving cellular homeostasis to restrict protein expression. In particular, decreased expression was observed in the gene encoding the CshA DEAD-box protein, which is associated with slow growth, ribosomal biogenesis, and RNA transcript degradation [67]. GSA identified significant enrichment of genes in the ribosome pathway in BR21 (Figure 2).

CREs are a major threat to successful treatment outcomes and an urgent and growing threat to public health [1, 4]. Here, we observed that emergence of high-level carbapenem-resistant subpopulations from a heteroresistant population of KPC-Kp is associated with differential adaptive response to stress initiated by an antibiotic. In two genotypically related strains of heteroresistant* K. pneumoniae* strains exposed to imipenem followed by incubation in drug-free medium, we observed (1) transcriptional changes that first involved general and osmotic stress response, followed by carbon source utilization and then protein processing, outer membrane integrity, and transport; (2) these sequential stress-adaptive responses to be associated with transient emergence (BR7) as well as irreversible appearance (BR21) of high-level imipenem resistant subpopulations; and (3) transcriptional changes associated with heritable loss of OmpK36 production and enrichment of subpopulations (BR21) that gained high-level CR.

We found that the loss of OmpK36 in the two strains exposed to imipenem followed distinct pathways. Imipenem-exposed BR21 samples showed changes in genes involved in potential modifications in the heptose core, which have been associated with translational repression of* ompC* in* E. coli* [68–70]. Strain BR7 had decreased expression of genes encoding a critical juncture of LPS synthesis that may result in the heterogeneity of LPS, or in the reduction of Lipid A in the outer membrane (Figure S2). Such changes could lead to failure of OmpK36 insertion into the outer membrane [68–70]. We also observed an increase in the noncoding RNA,* micA*, which may cause misfolded outer membrane proteins in response to lethal imipenem exposure [45]. Unlike BR21, which permanently lost OmpK36 as a result of an IS*1* insertion, OmpK36 loss in BR7 was reversible, perhaps via renaturation of the transiently misfolded protein. In the latter, once the drug was withdrawn, porin expression was restored, which may indicate a potential fitness advantage to this strain in the absence of drug exposure.

We previously showed that hydrolysis by KPC is required for imipenem-resistant KPC-Kp subpopulations to emerge from a relatively susceptible population [11]. Our present study suggests that KPC is necessary but not sufficient for KPC-Kp to gain high-level carbapenem resistance. Perhaps the first step involves KPC *β*-lactamase that hydrolyzes a relatively low concentration of imipenem entering the periplasm. This allows some subpopulations to survive. However, continued entry of the drug causes peptidoglycan damage. We propose that this damage induces general and osmotic stress responses followed by redirection of bacterial metabolism from glucose utilization to glutamate-dependent metabolism. This serves multiple purposes to set the stage for a catabolite-mediated adaptive response that feeds key metabolites into the TCA cycle. This response allows yet another set of subpopulations to survive long enough for abolished production of OmpK36, which leads to diminished cell entry of the drug and therefore high-level CR. Phage induction triggered by carbapenem-imposed stress may also play a role in this sequential adaptive response, as observed with BR21.

The limitation of our findings includes results that were obtained under* in vitro* laboratory conditions with controlled exposures. The transcriptional profile analysis was performed with RNA extracted from bacteria incubated as described in Materials and Methods in drug-free medium after they were exposed to imipenem for 2 and 8 hours, respectively, as analysis of cells immediately after drug exposure would have included mostly transcripts associated with dying cells. The high-level resistance of BR21 was irreversible, whereas BR7 reverted to its preexposure heteroresistant phenotype after incubation in drug-free medium. Thus, what we describe here is not directly related to imipenem exposure but the effect of bacterial propagation in drug-free medium for a subpopulation that survived the initial imipenem selective stress.

Despite these limitations, we observed distinct evolutionary pathways associated with appearance of high-level drug resistance of two carbapenem heteroresistant KPC-Kp strains exposed to a carbapenem prior to their incubation in drug-free medium. We suggest that high-level carbapenem resistance in heteroresistant* K. pneumoniae* strains results from sequential adaptive changes in response to stress first induced by a carbapenem. Further studies of additional CRE strains are required to confirm or generalize these observations.

These findings provide a rationale for potentially targeting the metabolic pathways we found to be involved in the emergence of CRE subpopulations. They may include inhibition of bacteria-specific polyamine and glutamate catabolic, as well as stress response, phage induction, and LPS synthetic pathways.

## 3. Materials and Methods

### 3.1. Study Strains and Sample Preparation

The KPC-Kp BR7 and BR21 strains in this study were obtained from bloodstream and urinary tract infections, respectively, collected from different hospitals in Rio de Janeiro, Brazil, in 2009. Their phenotypic imipenem heteroresistance was previously characterized [11]. No individual patient data was collected. Imipenem-exposed samples were obtained from dilution of overnight cultures standardized by optical density at 600 nm (OD_600_) to a starting inoculum of 10^6^ cfu/ml. Three biological replicates were each incubated in a lethal imipenem dose of 16 ug/ml for 2 or 8 hours at 37°C with shaking in Mueller-Hinton media (MH). The surviving cells were centrifuged for 15 minutes at 5000 ×g, resuspended in 2 ml drug-free cation-adjusted MH, and grown to midlogarithmic phase (OD_600_, 0.5). The time required to reach midlogarithmic phase varied from 5 to 6 hours for 8-hour exposed samples and 12 to 15 hours for 2-hour exposed samples. Unexposed control samples were prepared under the same conditions without imipenem and required 2-3 hours to achieve midlogarithmic phase growth. RNA was extracted with the Qiagen RNeasy MiniKit, after stabilization of RNA transcripts with the RNAprotect reagent, according to manufacturer's protocol (Qiagen, MD, USA). Genomic DNA was prepared from all strains with the Qiagen Blood and Tissue DNeasy kit.

### 3.2. Whole Genome/Transcriptome Sequencing

The number of biological replicates (3) was determined for transcriptome sequencing with published methods to optimize sequencing depth and achieve the power to detect differential expression [71]. RNA and DNA samples were submitted to the QB3 Functional Genomics Laboratory (UC Berkeley) for library preparation and cDNA synthesis (RNA). Study samples were submitted for whole genome sequencing on the Illumina platform at the QB3/Vincent J. Coates Genomics Sequencing Facility (UC Berkeley). Final libraries were quantified by Bioanalzyer and then sequenced via a 300 base-pair, paired-end run on a MiSeq instrument (genome), or via a 100 base-pair, paired-end run on a HiSeq 4000 instrument (transcriptome). Quality of Illumina sequences was analyzed with FastQC (Babraham Institute, Cambridge, UK). Illumina adaptors and low-quality sequences were trimmed with Geneious® version 9.0 (Biomatters, Ltd., New Zealand) or Trimmomatic 0.36 [72].

*K. pneumoniae* strains BR7 and BR21 were additionally sequenced on the Pacific Biosciences (PacBio) RSII sequencing platform (Pacific Biosciences, Menlo Park, California) at the University of California, Davis Genome Center. The data were de novo assembled with the PacBio hierarchical genome assembly pipeline (HGAP.2) and polished with the Quiver software package. For whole genome assembly, PacBio chromosomal and plasmid DNA sequences were used as reference sequences for mapping Illumina data from the imipenem-exposed study samples. Reads were mapped to plasmids and then to the chromosome with Geneious. Assemblies were submitted to RAST for annotation and subsystem ontological categorization [73]. Variant analysis was performed with the progressive Mauve algorithm running within Geneious. Highly variable phage (with the exception of annotated prophage regions), mobile elements, and intergenic regions were excluded from this portion of the analysis.

### 3.3. Whole Transcriptome Assembly and Statistical Analysis

Whole transcriptome assembly was performed on reads from triplicate biological samples indexed and mapped to PacBio reference sequences with Bowtie 2.2.3 and TopHat 2.0.13 (http://ccb.jhu.edu/software.shtml). Raw reads per sample are shown in Table S3. Uniquely mapped reads were sorted with SamTools 0.1.19 [74], and counts were obtained with HTSeq 0.6.1. [75]. Differential expression analysis was performed with EdgeR (R, 3.2.3) [76] with count data under a negative binomial model. Low counts were filtered; then remaining counts were normalized to correct for different composition of the sample read libraries. Statistically significant differential expression based on these counts was determined by Fisher's Exact Test to analyze pair-wise tests for differential expression between 3 biological replicates of the 2 h- or 8 h-imipenem-exposed samples compared to unexposed replicate samples and were reported as a log_2_-fold change of the resulting normalized differential expression counts. The topTags function subsequently adjusted the raw *p* values for multiple comparisons with the Benjamini-Hochberg False Discovery Rate (FDR) correction. Differentially expressed genes with FDR ≤ 0.05 were considered statistically significant. Pie chart and heat map figures were created with GraphPad Prism version 7 (GraphPad software, La Jolla, CA). Raw data, including dispersion of counts between biological replicates, is available as supplemental material (Table S3). Gene Set Analysis (GSA) was conducted to determine if differential expression of genes in certain groups was overrepresented in imipenem-exposed samples. The set of differentially expressed genes generated from EdgeR analysis were submitted to the KEGG Automated Annotation Server (KAAS, http://www.genome.jp/tools/kaas/) to generate KEGG orthology (KO) identifiers [77] and then used as input for Generally Applicable Gene-set Enrichment (GAGE) analysis running on the R/Bioconductor Pathview server (https://pathview.uncc.edu) [78–80]. An FDR *q*-value cutoff of 0.2 was used to assess pathway significance.

### 3.4. Accession Numbers

Complete genomes and plasmids for BR7 and BR21 are deposited in the NCBI database under Bioproject PRJNA358426 and Biosamples SAMN06173548 (BR7) and SAMN06173549 (BR21).

## Supplementary Materials

Supplementary Materials Figure S1: lethal imipenem exposure is associated with a global dampening of protein synthesis. Changes in outer membrane protein synthesis, protein transport and processing (Section 1, above the horizontal line), and protein translation (Section 2, below the horizontal line) were observed to a greater extent in BR21 than in BR7 at 8 h of imipenem exposure. Figure S2: transcriptional changes in the peptidoglycan biosynthetic pathway (Section 1, lines 1–8) and in key junctures of lipopolysaccharide (LPS) biosynthesis (Section 2, lines 9–23) after lethal imipenem exposure. At 8 h of exposure, genes involved in LPS synthesis were downregulated more prominently in BR21 than in B7. Table S1: characteristics of heteroresistant KPC-producing* K. pneumoniae* study strains, including source of infection, chromosome size, plasmid types and sizes, and carriage of prophage. Table S2: chromosomal sequence identity and variant analysis of KPC* K. pneumoniae* study strains and variant analysis between unexposed and imipenem-exposed samples. BR7 v BR21, chromosomal identity: Table S3: differentially expressed genes (FDR < 0.05) due to lethal IPM exposure of 2 h- and 8 h-exposed samples of heteroresistant KPC-producing* K. pneumoniae* strains. Chromosomal and plasmid-borne genes are shown separately. Raw reads by sample are shown at the bottom of the tables. Table S4: differentially expressed genes induced by stress regulons as a response to lethal imipenem exposure of heteroresistant KPC-producing* K. pneumoniae* study strains.
