# Supplementary material for: Stress-Adaptive Responses Associated with High-Level Carbapenem Resistance in KPC-Producing Klebsiella pneumoniae
Source: J Pathog. 2018 Mar 19;2018:3028290. doi: 10.1155/2018/3028290 (PMC5883989; doi:10.1155/2018/3028290)
Supplement: Supplementary Materials — Figure S1: lethal imipenem exposure is associated with a global dampening of protein synthesis. Changes in outer membrane protein synthesis, protein transport and processing (Section 1, above the horizontal line), and protein translation (Section 2, below the horizontal line) were observed to a greater extent in BR21 than in BR7 at 8 h of imipenem exposure. Figure S2: transcriptional changes in the peptidoglycan biosynthetic pathway (Section 1, lines 1–8) and in key junctures of lipopolysaccharide (LPS) biosynthesis (Section 2, lines 9–23) after lethal imipenem exposure. At 8 h of exposure, genes involved in LPS synthesis were downregulated more prominently in BR21 than in B7. Table S1: characteristics of heteroresistant KPC-producing K. pneumoniae study strains, including source of infection, chromosome size, plasmid types and sizes, and carriage of prophage. Table S2: chromosomal sequence identity and variant analysis of KPC K. pneumoniae study strains and variant analysis between unexposed and imipenem-exposed samples. BR7 v BR21, chromosomal identity: Table S3: differentially expressed genes (FDR < 0.05) due to lethal IPM exposure of 2 h- and 8 h-exposed samples of heteroresistant KPC-producing K. pneumoniae strains. Chromosomal and plasmid-borne genes are shown separately. Raw reads by sample are shown at the bottom of the tables. Table S4: differentially expressed genes induced by stress regulons as a response to lethal imipenem exposure of heteroresistant KPC-producing K. pneumoniae study strains. [file 3028290.f1.pdf]

SUPPLEMENTARY MATERIALS

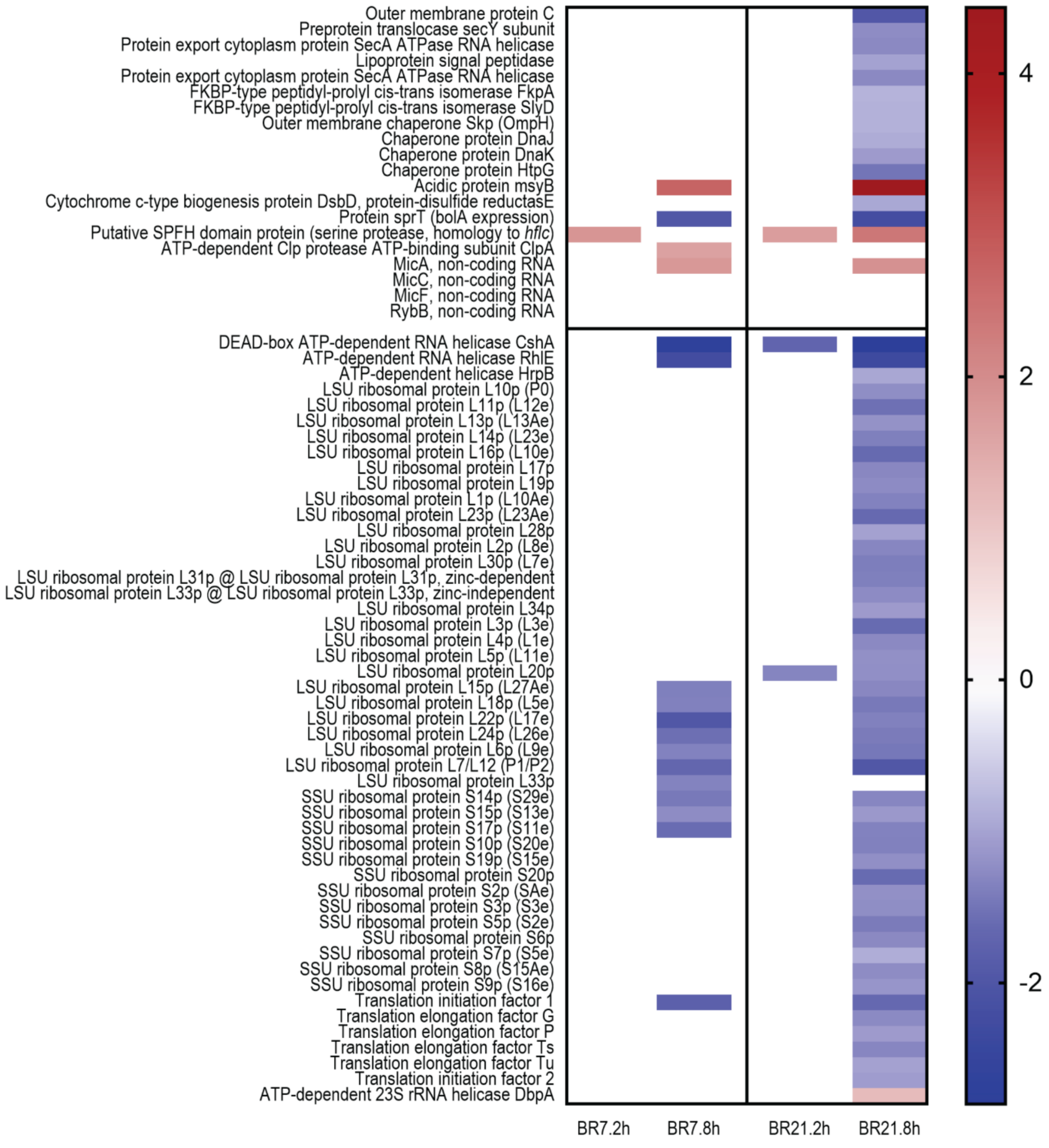

Fig. S1. **Lethal imipenem exposure is associated with a global dampening of protein synthesis.** Changes in outer membrane protein synthesis, protein transport and processing

(section 1, above the horizontal line), and protein translation (section 2, below the horizontal line) were observed to a greater extent in BR21 than in BR7 at 8h of imipenem exposure.

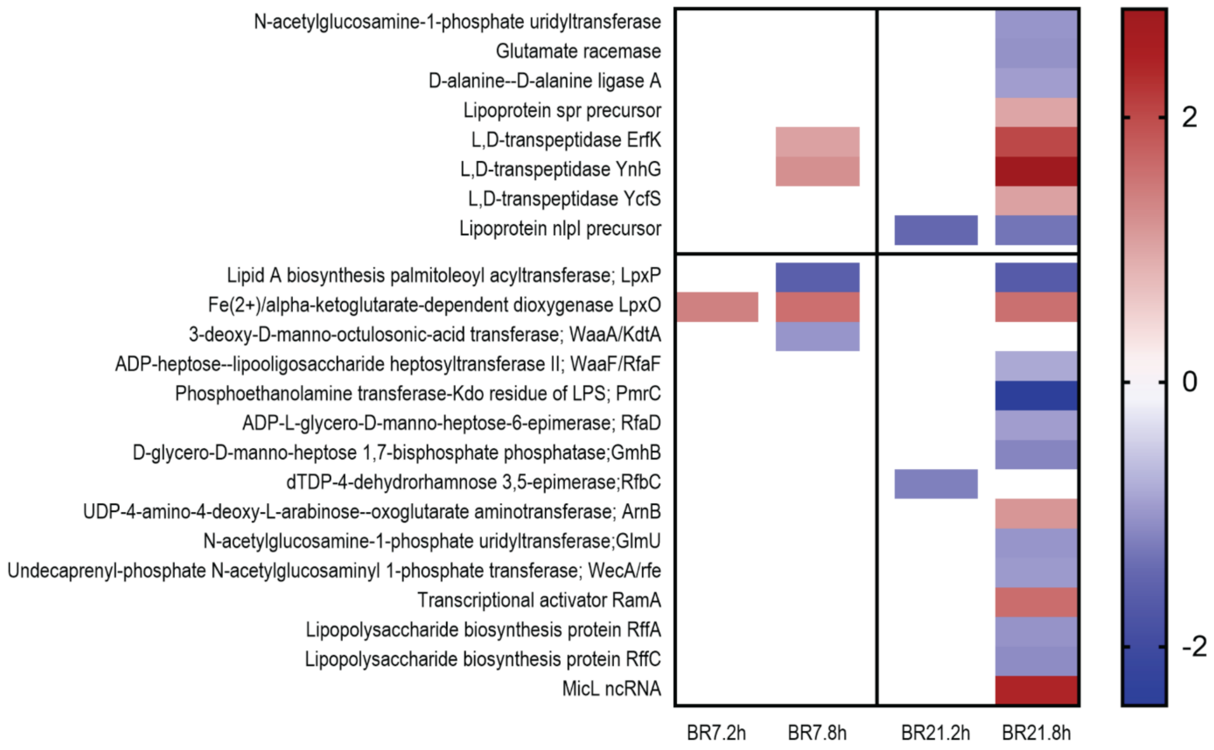

Fig. S2. **Transcriptional changes in the peptidoglycan biosynthetic pathway (section 1, lines 1-8), and in key junctures of lipopolysaccharide (LPS) biosynthesis (section 2, lines 9-23) after lethal imipenem exposure.** At 8h of exposure, genes involved in LPS synthesis were down-regulated more prominently in BR21 than in B7.

Table S1: Characteristics of heteroresistant KPC-producing *K. pneumoniae* study strains, including source of infection, chromosome size, plasmid types and sizes, and carriage of prophage.

	clonal group	source	chromosome size	plasmid Inc types, size	prophage	type	host
BR7	ST437 (CG258)	blood	5,308,011		Fels2, NC010463 (intact)	Caudovirales, Peduovirinae, P2-like	Enterobacteriaceae
					SEN34, NC028699 (intact)	Caudovirales, Podoviridae	*Salmonella* serovars
					Bphi-B1251, NC019541 (intact)	Caudovirales, Podoviridae	*Acinetobacter* spp.
					ST64B, NC004313 (intact)	Caudovirales, Mu-like	*Salmonella* serovars
					P4, NC001609 (partial)	Caudovirales, Peduovirinae, P2-like	Enterobacteriaceae
				pQIL (FIB, N), 112 kb (harbors *bla* $_{KPC}$)	SPBc2, NC001884 (partial)	Caudovirales, Siphoviridae	*Bacillus* spp.
					FiAA91-ss, NC022750 (partial)	Caudovirales, Peduovirinae, P2-like	Enterobacteriaceae
					P1, NC005856 (partial)	Caudovirales, Myoviridae, P1	Enterobacteriaceae
BR21	ST437 (CG258)	urine	5,368,098		SPN1S, NC016761 (intact)	Caudovirales, Podoviridae	*Salmonella* serovars
					Fels2, NC010463 (intact)	Caudovirales, Peduovirinae, P2-like	Enterobacteriaceae
					HK639, NC016158 (intact)	Caudovirales, Siphoviridae, Lambda-like	*E. coli*
					SEN34, NC028699 (intact)	Caudovirales, Podoviridae	*Salmonella* serovars
					ST64B, NC004313 (partial)	Caudovirales, Mu-like	*Salmonella* serovars
					P4, NC001609 (partial)	Caudovirales, Peduovirinae, P2-like	Enterobacteriaceae
					TL2011b, NC019445 (partial)	Caudovirales, Podoviridae	*E. coli*
				IncF, 214 kb	P1, NC005856 (partial)	Caudovirales, Myoviridae, P1	Enterobacteriaceae
					Ectocarpus siliculosus virus 1, NC002687 (partial)	Phycodnaviridae, Phaeovirus	marine brown algae
					Spbeta-like, NC029119 (partial)	Caudovirales, Siphoviridae	*Staphylococcus* spp.
				IncN, 121 kb (harbors *bla* $_{KPC}$)	P1, NC005856 (partial)	Caudovirales, Myoviridae, P1	Enterobacteriaceae
					Stx2-converting phage 1717, NC011357 (partial)	Caudovirales, Siphoviridae, Lambda-like	*E. coli* O157:H7

Table S2. Chromosomal sequence identity and variant analysis of KPC *K. pneumoniae* study strains, and variant analysis between unexposed and imipenem-exposed samples.
BR7 v BR21, chromosomal identity:

product	Protein Effect	SNV (functional)	SNV (silent)	MNP	indels	variant nucleotide(s)	Minimum	Maximum	Length	Polymorphism Type	Variant P-Value (approximate)	Amino Acid Change	CDS Position
ADA regulatory protein / Methylated-DNA--protein-cysteine methyltransferase (EC 2.1.1.63)	Substitution	1				C	1,263,876	1,263,876	1	SNP (transition)	0.01	S -> P	118
Adenylosuccinate lyase (EC 4.3.2.2)	Substitution	1				T	3,480,667	3,480,667	1	SNP (transversion)	0.01	I -> N	836
Aldehyde dehydrogenase (EC 1.2.1.3)	Extension	0	1			T	187,193	187,193	1	SNP (transversion)	0.01		1,240
Carbon starvation protein A	Substitution	1				A	2,901,651	2,901,651	1	SNP (transversion)	0.01	A -> D	1,550
Cellulose synthase operon protein C	Extension	0			1		1,150,481	1,154,530	4,050	Deletion	0.1		4
Cyanate transport protein CynX	Substitution	1				A	5,191,198	5,191,198	1	SNP (transition)	0.01	M -> I	426
Cyclic di-GMP binding protein precursor	Extension	**0**			**1**		1,154,530	1,154,532	3	Deletion	0.1		2,434
Di/tripeptide permease DtpA	Substitution	1				T	4,382,479	4,382,479	1	SNP (transition)	0.01	P -> S	1,009
DNA helicase IV	Substitution	1				A	3,344,596	3,344,596	1	SNP (transversion)	0.01	L -> M	979
Endoglucanase precursor (EC 3.2.1.4)	Extension	0			1		1,148,992	1,149,993	1,002	Deletion	0.1		1
Exonuclease SbcD	Substitution	1				G	2,557,445	2,557,445	1	SNP (transversion)	0.01	R -> P	398
FIG002337: predicted inner membrane protein	Extension	0			1		1,147,215	1,148,884	1,670	Deletion	0.1		11
FIG00613381: hypothetical protein	Extension	0			1		1,150,005	1,150,481	477	Deletion	0.1		1
FIG00731367: hypothetical protein	Substitution	1				G	1,828,190	1,828,190	1	SNP (transversion)	0.01	L -> F	1,446
FIG01046265: hypothetical protein	Substitution	1				G	3,399,312	3,399,312	1	SNP (transversion)	0.01	D -> A	434
Glucosamine--fructose-6-phosphate aminotransferase [isomerizing] (EC 2.6.1.16)	Substitution	1				A	2,123,552	2,123,552	1	SNP (transversion)	0.01	S -> T	361
Glucose-1-phosphatase (EC 3.1.3.10)	Substitution	**1**				G	3,372,816	3,372,816	1	SNP (transition)	0.01	M -> V	19
Mobile element (HokE protein)	Truncation	**0**			**1**	AGAGCAATGT	290,778	290,777	0	Insertion	0.01		81
hypothetical protein	Extension	0			1		2,009,528	2,009,755	228	Deletion	0.1		1
LysR-family transcriptional regulator STM3020	Substitution	1				T	464,901	464,901	1	SNP (transition)	0.01	V -> I	34
Maltose/maltodextrin transport ATP-binding protein MalK (EC 3.6.3.19)	Substitution	1				A	1,720,316	1,720,316	1	SNP (transition)	0.01	G -> E	107
Mobile element protein	Extension	0			1		2,009,932	2,010,177	246	Deletion	0.1		1
Multidrug transporter MdtC	Substitution	1				T	4,957,204	4,957,204	1	SNP (transversion)	0.01	A -> S	2,824
Possible hydrolase or acyltransferase RutD in novel pyrimidine catabolism pathway	Substitution	1				A	3,380,073	3,380,073	1	SNP (transversion)	0.01	G -> V	260
PTS system, chitobiose-specific IIB component (EC 2.7.1.69)	Substitution	1				C	3,578,257	3,578,257	1	SNP (transition)	0.01	V -> A	314
Putative cytoplasmic protein USSDB7A	Substitution	1				T	3,790,507	3,790,507	1	SNP (transition)	0.01	L -> F	259
Putative major fimbrial subunit	Substitution	1				C	1,777,298	1,777,298	1	SNP (transversion)	0.01	Q -> P	413
Putative membrane protein	Substitution	1				A	663,835	663,835	1	SNP (transition)	0.01	A -> V	50
Redox-sensing transcriptional regulator QorR	Frame Shift	0			1	C	1,907,721	1,907,720	0	Insertion	0.01		288
Respiratory nitrate reductase alpha chain (EC 1.7.99.4)	Substitution	1				G	4,613,705	4,613,705	1	SNP (transversion)	0.01	I -> L	2,866
Ribose ABC transport system, permease protein RbsC (TC 3.A.1.2.1)	Substitution	**0**		**1**		TC	2,907,748	2,907,749	2	Substitution	0.01	L -> S	826

Signal recognition particle receptor protein FtsY (=alpha subunit) (TC 3.A.5.1.1)	Substitution	1				T	1,078,728	1,078,728	1	SNP (transition)	0.01	V -> I	925
sugar-proton symporter	Substitution	1				G	42,039	42,039	1	SNP (transversion)	0.01	G -> A	236
Survival protein SurA precursor (Peptidyl-prolyl cis-trans isomerase SurA) (EC 5.2.1.8)	Substitution	1				A	2,226,905	2,226,905	1	SNP (transversion)	0.01	Q -> L	1,178
Transcriptional regulatory protein PhoP	Substitution	1				A	3,478,454	3,478,454	1	SNP (transversion)	0.01	A -> S	88
Tyrosine-protein kinase Wzc (EC 2.7.10.2)	Substitution	1				G	4,927,698	4,927,698	1	SNP (transversion)	0.01	T -> P	1,927
UDP-N-acetylmuramate:L-alanyl-gamma-D-glutamyl- meso-diaminopimelate ligase (EC 6.3.2.-)	Substitution	1				G	1,930,986	1,930,986	1	SNP (transition)	0.01	D -> G	1,316
Uracil phosphoribosyltransferase (EC 2.4.2.9) CDS	Substitution	0	1			A	21,337	21,963	1	SNP			
VgrG protein	Truncation	**0**			**1**	GCCGTCGTAT	4,671,707	4,671,706	0	Insertion	0.01		196
3-oxoacyl-[acyl-carrier-protein] synthase, KASII (EC 2.3.1.179)	None	0				T	4,176,067	4,176,067	1	SNP (transversion)	0.01		648
6-phospho-beta-glucosidase (EC 3.2.1.86)	None	0				A	3,128,933	3,128,933	1	SNP (transition)	0.01		972
ABC transporter, permease protein YnjC	None	0				A	3,562,870	3,562,870	1	SNP (transversion)	0.01		921
glutamyl-Q-tRNA synthetase	None	0				T	2,344,119	2,344,119	1	SNP (transition)	0.01		69
Hydroxymethylpyrimidine ABC transporter, substrate-binding component	None	0				A	4,200,662	4,200,662	1	SNP (transition)	0.01		789
Nucleoside-diphosphate-sugar epimerases	None	0				A	2,598,575	2,598,575	1	SNP (transition)	0.01		1,062
outside CDS	none	0				A	229,031	229,031	1	SNP (transversion)	0.01		
outside CDS	none	0				T	357,590	357,589	0	Insertion (tandem repeat)	0.01		
outside CDS	none	0				T	776,201	776,201	1	SNP (transversion)	0.01		
outside CDS	none	0				C	2,840,713	2,840,713	1	SNP (transition)	0.01		
outside CDS	none	0				T	3,059,710	3,059,709	0	Insertion (tandem repeat)	0.01		
outside CDS	none	0				A	3,403,997	3,403,997	1	SNP (transversion)	0.01		
outside CDS	none	0				T	3,617,754	3,617,754	1	SNP (transversion)	0.01		
outside CDS	none	0				C	3,750,252	3,750,252	1	SNP (transition)	0.01		
outside CDS	none	0				T	4,412,889	4,412,889	1	SNP (transversion)	0.01		
outside CDS	none	0				T	4,544,142	4,544,142	1	SNP (transition)	0.01		
outside CDS	none	0				C	4,671,983	4,671,983	1	SNP (transversion)	0.01		
Para-aminobenzoate synthase, aminase component (EC 2.6.1.85)	None	0				C	4,735,945	4,735,945	1	SNP (transversion)	0.01		681
Phage terminase, ATPase subunit	None	0				A	140,267	140,267	1	SNP (transversion)	0.01		138
Putative ion-channel protein	None	0				T	5,201,770	5,201,770	1	SNP (transition)	0.01		921
Pyruvate-flavodoxin oxidoreductase (EC 1.2.7.-)	None	0				C	3,841,999	3,841,999	1	SNP (transition)	0.01		2,610
regulator of length of O-antigen component of lipopolysaccharide chains	None	0				T	1,573,212	1,573,212	1	SNP (transition)	0.01		438
transcriptional regulator, RpiR family	None	0				C	1,293,713	1,293,713	1	SNP (transition)	0.01		519
tRNA-guanine transglycosylase (EC 2.4.2.29)	None	0				G	2,577,097	2,577,097	1	SNP (transition)	0.01		261
			2	1	10								

BR7 v BR21 chromosomal summary:

functional, SNP (non-synonymous)	26
silent (synonymous), inside CDS	2
MNP (non-synonymous)	1
MNP, insertions or deletions	10
polymorphisms outside CDS	11
	50

	BR21 reference	BR7 nucleotide identity	% nucleotide identity BR21 v BR7
Length LCB 1	5,262,336		
Length LCB 2	38,801		
	5,301,137	5,301,069	99.9987%

BR7 vs 2h-, 8h-imipenem-exposed samples:

BR7, 2h-exposed	none
BR7, 8h-exposed	none

BR21 vs 2h-, 8h-imipenem-exposed samples:

BR21, 2h-exposed	none
BR21, 8h-exposed	IS*1* insertion, *ompK36* coding region

Table S3. Differentially expressed genes (FDR < 0.05) due to lethal IPM exposure of 2h- and 8h-exposed samples of heteroresistant KPC-producing *K.pneumoniae strains.*
Chromosomal and Plasmid-borne genes are shown separately. Raw reads by sample are shown at the bottom of the tables.

Chromosomal Genes (Plasmid-borne genes listed separately below)	**SEED CATEGORY**	**SEED SUBCATEGORY**	BR7-2h-up	BR7-2h-down	BR21-2h-up	BR21-2h-down	BR7-8h-up	BR7-8h-down	BR21-8h-up	BR21-8h-down
2,4-dihydroxyhept-2-ene-1,7-dioic acid aldolase (EC 4.1.2.-)	Amino Acids and Derivatives	Aromatic amino acids and	0	0	0	0	1	0	1	0
3,4-dihydroxyphenylacetate 2,3-dioxygenase (EC 1.13.11.15)	Amino Acids and Derivatives	Aromatic amino acids and	0	0	0	0	1	0	1	0
4-aminobutyraldehyde dehydrogenase (EC 1.2.1.19)	Amino Acids and Derivatives	Arginine; urea cycle, polya	0	0	0	0	1	0	1	0
4-hydroxyphenylpyruvate dioxygenase (EC 1.13.11.27)	Amino Acids and Derivatives	Aromatic amino acids and	0	0	0	0	1	0	0	0
5-carboxymethyl-2-hydroxymuconate delta-isomerase (EC 5.3.3.10)	Amino Acids and Derivatives	Aromatic amino acids and	0	0	0	0	0	0	1	0
5-carboxymethyl-2-hydroxymuconate semialdehyde dehydrogenase (EC 1.2.1.60)	Amino Acids and Derivatives	Aromatic amino acids and	1	0	0	0	1	0	1	0
5-carboxymethyl-2-oxo-hex-3- ene-1,7-dioate decarboxylase (EC 4.1.1.68) / 2-hyd	Amino Acids and Derivatives	Aromatic amino acids and	1	0	0	0	1	0	1	0
5-methyltetrahydropteroyltriglutamate-- homocysteine methyltransferase (EC 2.1	Amino Acids and Derivatives	Lysine, threonine, methion	0	0	0	0	0	0	1	0
Acetolactate synthase small subunit (EC 2.2.1.6)	Amino Acids and Derivatives	Branched-chain amino aci	0	0	0	0	0	0	1	0
Acetylglutamate kinase (EC 2.7.2.8)	Amino Acids and Derivatives	Arginine; urea cycle, polya	0	0	0	0	0	0	1	0
Acetylornithine aminotransferase (EC 2.6.1.11)	Amino Acids and Derivatives	Arginine; urea cycle, polya	0	0	0	0	0	0	1	0
Adenylylsulfate kinase (EC 2.7.1.25)	Amino Acids and Derivatives	Lysine, threonine, methion	0	1	0	0	0	0	0	0
Alpha-ketoglutarate permease	Amino Acids and Derivatives	Glutamine, glutamate, aspa	1	0	0	0	1	0	1	0
Aminomethyltransferase (glycine cleavage system T protein) (EC 2.1.2.10)	Amino Acids and Derivatives	Alanine, serine, and glycine	0	0	1	0	0	0	1	0
Anthranilate synthase, amidotransferase component (EC 4.1.3.27)	Amino Acids and Derivatives	Aromatic amino acids and	0	0	0	0	0	0	1	0
Anthranilate synthase, aminase component (EC 4.1.3.27)	Amino Acids and Derivatives	Aromatic amino acids and	0	0	0	0	0	0	1	0
Arginine ABC transporter, ATP-binding protein ArtP	Amino Acids and Derivatives	Arginine; urea cycle, polya	0	0	0	0	0	0	1	0
Arginine ABC transporter, periplasmic arginine-binding protein ArtJ	Amino Acids and Derivatives	Arginine; urea cycle, polya	0	0	0	0	0	0	1	0
Arginine exporter protein ArgO	Amino Acids and Derivatives	Arginine; urea cycle, polya	0	0	0	0	0	0	1	0
Arginine N-succinyltransferase (EC 2.3.1.109); AstA	Amino Acids and Derivatives	Arginine; urea cycle, polya	1	0	1	0	1	0	1	0
AroM protein	Amino Acids and Derivatives	Aromatic amino acids and	0	0	0	0	0	0	1	0
Aromatic amino acid transport protein AroP	Amino Acids and Derivatives	Aromatic amino acids and	0	0	0	0	1	0	1	0
Asparagine synthetase [glutamine-hydrolyzing] (EC 6.3.5.4)	Amino Acids and Derivatives	Glutamine, glutamate, aspa	0	0	0	1	0	0	0	1
Aspartate--ammonia ligase (EC 6.3.1.1); asnA	Amino Acids and Derivatives	Glutamine, glutamate, aspa	0	0	0	1	0	1	0	1
Aspartokinase (EC 2.7.2.4) / Homoserine dehydrogenase (EC 1.1.1.3)	Amino Acids and Derivatives	Lysine, threonine, methion	0	0	0	0	0	0	0	1
Biosynthetic Aromatic amino acid aminotransferase alpha (EC 2.6.1.57)	Amino Acids and Derivatives	Aromatic amino acids and	0	0	0	0	1	0	0	0
COG2907: Amine oxidase, flavin-containing	Amino Acids and Derivatives	Arginine; urea cycle, polya	0	0	0	0	0	0	0	1
Conserved hypothetical protein (perhaps related to histidine degradation)	Amino Acids and Derivatives	Histidine Metabolism	0	0	0	0	1	0	1	0
Cysteine desulfurase (EC 2.8.1.7), SufS subfamily	Amino Acids and Derivatives	Alanine, serine, and glycine	0	0	0	0	0	0	1	0
Cytosine deaminase (EC 3.5.4.1)	Amino Acids and Derivatives	Amino Acids and Derivativ	0	0	0	0	1	0	0	0
Diaminobutyrate--2-oxoglutarate aminotransferase (EC 2.6.1.76)	Amino Acids and Derivatives	Polyamines	0	0	0	0	0	1	0	1
Diaminopimelate decarboxylase (EC 4.1.1.20)	Amino Acids and Derivatives	Lysine, threonine, methion	0	0	0	0	1	0	0	0
Diaminopimelate epimerase (EC 5.1.1.7)	Amino Acids and Derivatives	Lysine, threonine, methion	0	0	0	0	0	0	0	1
D-serine deaminase (EC 4.3.1.18)	Amino Acids and Derivatives	Alanine, serine, and glycine	0	0	0	0	0	0	1	0
D-serine dehydratase (EC 4.3.1.18)	Amino Acids and Derivatives	Alanine, serine, and glycine	0	0	0	0	0	1	0	1
D-serine dehydratase transcriptional activator	Amino Acids and Derivatives	Alanine, serine, and glycine	0	0	0	0	0	0	0	1
D-serine permease DsdX	Amino Acids and Derivatives	Alanine, serine, and glycine	1	0	0	0	0	0	0	1
D-serine/D-alanine/glycine transporter	Amino Acids and Derivatives	Alanine, serine, and glycine	1	0	0	0	1	0	1	0
FIG002283: Isochorismatase family protein	Amino Acids and Derivatives	Aromatic amino acids and	0	0	0	0	0	0	1	0
Formiminoglutamase (EC 3.5.3.8)	Amino Acids and Derivatives	Histidine Metabolism	0	0	0	0	1	0	1	0
gamma-aminobutyrate (GABA) permease; GabP	Amino Acids and Derivatives	Arginine; urea cycle, polya	1	0	0	0	1	0	1	0
Gamma-aminobutyrate:alpha-ketoglutarate aminotransferase, GabT (EC 2.6.1.19)	Amino Acids and Derivatives	Arginine; urea cycle, polya	0	0	0	0	1	0	1	0
Gamma-glutamyl-aminobutyraldehyde dehydrogenase (EC 1.2.1.-); puuC/GabD	Amino Acids and Derivatives	Arginine; urea cycle, polya	1	0	1	0	1	0	1	0
Gamma-glutamyl-GABA hydrolase (EC 3.5.1.94); PuuD	Amino Acids and Derivatives	Arginine; urea cycle, polya	1	0	0	0	1	0	1	0
Gamma-glutamyl-putrescine oxidase (EC1.4.3.-); PuuB	Amino Acids and Derivatives	Arginine; urea cycle, polya	1	0	0	0	1	0	1	0
Gamma-glutamyl-putrescine synthetase (EC 6.3.1.11); PuuA	Amino Acids and Derivatives	Arginine; urea cycle, polya	1	0	0	0	1	0	1	0
Glutamate Aspartate periplasmic binding protein precursor GltI (TC 3.A.1.3.4)	Amino Acids and Derivatives	Glutamine, glutamate, aspa	1	0	0	0	1	0	1	0
Glutamate Aspartate transport ATP-binding protein GltL (TC 3.A.1.3.4)	Amino Acids and Derivatives	Glutamine, glutamate, aspa	1	0	0	0	1	0	1	0

Chromosomal Genes (Plasmid-borne genes listed separately below)	SEED CATEGORY	SEED SUBCATEGORY	BR7-2h-up	BR7-2h-down	BR21-2h-up	BR21-2h-down	BR7-8h-up	BR7-8h-down	BR21-8h-up	BR21-8h-down
Glutamate Aspartate transport system permease protein GltJ (TC 3.A.1.3.4)	Amino Acids and Derivatives	Glutamine, glutamate, aspa	1	0	0	0	1	0	1	0
Glutamate Aspartate transport system permease protein GltK (TC 3.A.1.3.4)	Amino Acids and Derivatives	Glutamine, glutamate, aspa	1	0	0	0	1	0	1	0
Glutamate racemase (EC 5.1.1.3)	Amino Acids and Derivatives	Glutamine, glutamate, aspa	0	0	0	0	0	0	0	1
Glutamate transport ATP-binding protein (aka glnQ)	Amino Acids and Derivatives	Glutamine, glutamate, aspa	0	0	0	0	1	0	0	0
Glutamine synthetase type I (EC 6.3.1.2)	Amino Acids and Derivatives	Glutamine, glutamate, aspa	0	0	0	0	0	1	0	1
Glycine cleavage system H protein	Amino Acids and Derivatives	Alanine, serine, and glycine	0	0	0	0	0	0	1	0
Glycine dehydrogenase [decarboxylating] (glycine cleavage system P protein) (EC 1	Amino Acids and Derivatives	Alanine, serine, and glycine	0	0	0	0	0	0	1	0
Histidine ABC transporter, ATP-binding protein HisP (TC 3.A.1.3.1)	Amino Acids and Derivatives	Arginine; urea cycle, polyar	0	0	0	0	1	0	0	0
Histidine ammonia-lyase (EC 4.3.1.3)	Amino Acids and Derivatives	Histidine Metabolism	1	0	1	0	1	0	1	0
Histidine transport protein (permease)	Amino Acids and Derivatives	Histidine Metabolism	1	0	0	0	1	0	1	0
Histidine utilization repressor	Amino Acids and Derivatives	Histidine Metabolism	0	0	0	0	0	0	1	0
Homoserine kinase (EC 2.7.1.39)	Amino Acids and Derivatives	Lysine, threonine, methion	0	0	0	0	0	0	0	1
Imidazolonepropionase (EC 3.5.2.7)	Amino Acids and Derivatives	Histidine Metabolism	0	0	0	0	1	0	1	0
Indole-3-glycerol phosphate synthase (EC 4.1.1.48)	Amino Acids and Derivatives	Aromatic amino acids and	0	0	0	0	0	0	1	0
Isochorismate synthase (EC 5.4.4.2) of siderophore biosynthesis; entC	Amino Acids and Derivatives	Aromatic amino acids and	0	0	1	0	0	1	1	0
Ketol-acid reductoisomerase (EC 1.1.1.86)	Amino Acids and Derivatives	Branched-chain amino acid	0	1	0	0	0	1	0	1
L-2,4-diaminobutyrate decarboxylase (EC 4.1.1.86)	Amino Acids and Derivatives	Polyamines	0	0	0	0	0	1	0	1
L-asparagine permease	Amino Acids and Derivatives	Glutamine, glutamate, aspa	0	0	0	0	0	0	1	0
L-threonine transporter, anaerobically inducible	Amino Acids and Derivatives	Lysine, threonine, methion	0	0	0	0	0	0	1	0
Lysine/cadaverine antiporter membrane protein CadB	Amino Acids and Derivatives	Lysine, threonine, methion	0	0	0	0	0	0	0	1
Lysine-arginine-ornithine-binding periplasmic protein precursor (TC 3.A.1.3.1)	Amino Acids and Derivatives	Arginine; urea cycle, polyar	1	0	0	0	1	0	1	0
Methionine synthase II (cobalamin-independent)	Amino Acids and Derivatives	Lysine, threonine, methion	0	0	0	0	0	0	1	0
Monoamine oxidase (1.4.3.4)	Amino Acids and Derivatives	Lysine, threonine, methion	0	0	0	0	0	0	1	0
N-acetyl-gamma-glutamyl-phosphate reductase (EC 1.2.1.38)	Amino Acids and Derivatives	Arginine; urea cycle, polyar	0	0	0	0	0	0	1	0
NADP/NAD-specific glutamate dehydrogenase (EC 1.4.1.2)(EC 1.4.1.4)	Amino Acids and Derivatives	Glutamine, glutamate, aspa	1	0	1	0	1	0	1	0
Predicted chaperone lipoprotein YacC, potentially involved in protein secretion	Amino Acids and Derivatives	Arginine; urea cycle, polyar	0	0	0	0	0	0	0	1
Predicted glutamine amidotransferase	Amino Acids and Derivatives	Glutamine, glutamate, aspa	0	0	0	0	0	0	0	1
Proline/sodium symporter PutP (TC 2.A.21.2.1)	Amino Acids and Derivatives	Proline and 4-hydroxyproli	1	0	0	0	1	0	1	0
Proline-specific permease proY	Amino Acids and Derivatives	Proline and 4-hydroxyproli	0	0	0	0	0	0	0	1
Putative alanine/glycine transport protein	Amino Acids and Derivatives	Alanine, serine, and glycine	0	0	0	0	0	1	0	1
Putrescine ABC transporter putrescine-binding protein PotF (TC 3.A.1.11.2)	Amino Acids and Derivatives	Arginine; urea cycle, polyar	0	0	0	0	1	0	1	0
Putrescine aminotransferase (EC 2.6.1.82); patA	Amino Acids and Derivatives	Arginine; urea cycle, polyar	0	0	0	0	1	0	1	0
Putrescine transport ATP-binding protein PotG (TC 3.A.1.11.2)	Amino Acids and Derivatives	Arginine; urea cycle, polyar	0	0	0	0	0	0	1	0
Putrescine transport system permease protein PotH (TC 3.A.1.11.2)	Amino Acids and Derivatives	Arginine; urea cycle, polyar	0	0	0	0	0	0	1	0
Putrescine utilization regulator	Amino Acids and Derivatives	Arginine; urea cycle, polyar	0	0	0	0	1	0	1	0
Pyruvate dehydrogenase E1 component (EC 1.2.4.1)	Amino Acids and Derivatives	Lysine, threonine, methion	0	0	0	0	0	1	0	1
Siroheme synthase / Precorrin-2 oxidase (EC 1.3.1.76) / Sirohydrochlorin ferrochel	Amino Acids and Derivatives	Lysine, threonine, methion	0	0	0	0	0	0	0	1
Sodium/glutamate symport protein	Amino Acids and Derivatives	Glutamine, glutamate, aspa	0	0	0	0	1	0	1	0
Spermidine N1-acetyltransferase (EC 2.3.1.57)	Amino Acids and Derivatives	Arginine; urea cycle, polyar	0	0	0	0	0	1	0	0
Succinylarginine dihydrolase (EC 3.5.3.23); AstB	Amino Acids and Derivatives	Arginine; urea cycle, polyar	1	0	0	0	1	0	1	0
Succinylglutamate desuccinylase (EC 3.5.1.96); AstE	Amino Acids and Derivatives	Arginine; urea cycle, polyar	1	0	0	0	1	0	1	0
Succinylglutamic semialdehyde dehydrogenase (EC 1.2.1.71); AstD	Amino Acids and Derivatives	Arginine; urea cycle, polyar	1	0	0	0	1	0	1	0
Succinylornithine transaminase (EC 2.6.1.81); AstC	Amino Acids and Derivatives	Arginine; urea cycle, polyar	1	0	1	0	1	0	1	0
Sulfate adenylyltransferase subunit 1 (EC 2.7.7.4)	Amino Acids and Derivatives	Lysine, threonine, methion	0	1	0	0	0	0	0	0
Sulfate and thiosulfate import ATP-binding protein CysA (EC 3.6.3.25)	Amino Acids and Derivatives	Lysine, threonine, methion	0	1	0	0	0	0	0	0
Sulfate permease	Amino Acids and Derivatives	Lysine, threonine, methion	0	0	1	0	0	0	1	0
Threonine dehydratase, catabolic (EC 4.3.1.19) TdcB	Amino Acids and Derivatives	Lysine, threonine, methion	0	0	0	0	0	0	1	0
Trans-aconitate 2-methyltransferase (EC 2.1.1.144)	Amino Acids and Derivatives	Lysine, threonine, methion	0	0	0	0	0	0	1	0
Transcriptional regulator GabR of GABA utilization (GntR family with aminotransfer	Amino Acids and Derivatives	Lysine, threonine, methion	1	0	1	0	1	0	1	0
Transcriptional repressor of PutA and PutP	Amino Acids and Derivatives	Proline and 4-hydroxyproli	1	0	0	0	1	0	1	0

Chromosomal Genes (Plasmid-borne genes listed separately below)	SEED CATEGORY	SEED SUBCATEGORY	BR7-2h-up	BR7-2h-down	BR21-2h-up	BR21-2h-down	BR7-8h-up	BR7-8h-down	BR21-8h-up	BR21-8h-down
Tryptophan synthase alpha chain (EC 4.2.1.20)	Amino Acids and Derivatives	Aromatic amino acids and	0	0	0	0	0	0	1	0
Tryptophan synthase beta chain (EC 4.2.1.20)	Amino Acids and Derivatives	Aromatic amino acids and	0	0	0	0	0	0	1	0
Urease gamma subunit (EC 3.5.1.5)	Amino Acids and Derivatives	Arginine; urea cycle, polyar	0	0	0	0	1	0	0	0
Urocanate hydratase (EC 4.2.1.49)	Amino Acids and Derivatives	Histidine Metabolism	1	0	0	0	1	0	1	0
1,4-alpha-glucan (glycogen) branching enzyme, GH-13-type (EC 2.4.1.18)	Carbohydrates	Polysaccharides	0	0	0	0	0	0	1	0
1-phosphofructokinase (EC 2.7.1.56)	Carbohydrates	Monosaccharides	0	0	1	0	0	0	1	0
2-(5''-triphosphoribosyl)-3'-dephosphocoenzyme- A synthase (EC 2.7.8.25)	Carbohydrates	Central carbohydrate meta	0	0	0	0	1	0	0	0
2,3-bisphosphoglycerate-independent phosphoglycerate mutase (EC 5.4.2.1)	Carbohydrates	Central carbohydrate meta	0	0	0	0	0	0	0	1
2,4-dienoyl-CoA reductase [NADPH] (EC 1.3.1.34)	Carbohydrates	Central carbohydrate meta	0	0	0	0	0	0	1	0
2-dehydro-3-deoxyglucarate aldolase (EC 4.1.2.20)	Carbohydrates	Monosaccharides	0	1	0	0	0	0	0	0
2-dehydro-3-deoxy-L-rhamnonate aldolase (EC 4.1.2.n3)	Carbohydrates	Carbohydrates - no subcat	0	0	0	0	0	0	1	0
2-ketogluconate 6-phosphate reductase (EC 1.1.1.43)	Carbohydrates	Monosaccharides	0	0	0	0	1	0	0	0
2-ketogluconate kinase (EC 2.7.1.13)	Carbohydrates	Monosaccharides	0	0	0	0	1	0	0	0
2-ketogluconate transporter	Carbohydrates	Monosaccharides	0	0	0	0	1	0	1	0
2-oxoglutarate dehydrogenase E1 component (EC 1.2.4.2)	Carbohydrates	Central carbohydrate meta	0	0	0	0	0	0	1	0
2-oxoglutarate/malate translocator	Carbohydrates	Central carbohydrate meta	0	0	1	0	0	0	1	0
4-alpha-glucanotransferase (amylomaltase) (EC 2.4.1.25)	Carbohydrates	Di- and oligosaccharides	0	0	0	0	0	0	0	1
5-deoxy-glucuronate isomerase (EC 5.3.1.-)	Carbohydrates	Sugar alcohols	0	0	0	0	0	0	1	0
6-phosphofructokinase (EC 2.7.1.11)	Carbohydrates	Central carbohydrate meta	0	0	0	0	0	0	0	1
6-phosphofructokinase class II (EC 2.7.1.11); pfkB (FDP)	Carbohydrates	Central carbohydrate meta	0	0	0	0	0	0	1	0
Acetaldehyde dehydrogenase (EC 1.2.1.10)	Carbohydrates	Central carbohydrate meta	0	0	0	0	0	0	0	1
Acetaldehyde dehydrogenase, ethanolamine utilization cluster (EC 1.2.1.10)	Carbohydrates	Sugar alcohols	0	0	0	0	0	0	1	0
Acetate kinase (EC 2.7.2.1); ackA	Carbohydrates	Sugar alcohols	0	0	0	0	0	1	0	1
Acetate operon repressor	Carbohydrates	Central carbohydrate meta	0	0	0	0	1	0	0	0
Acetate permease ActP (cation/acetate symporter)	Carbohydrates	Central carbohydrate meta	1	0	0	0	1	0	1	0
Acetolactate synthase, catabolic (EC 2.2.1.6)	Carbohydrates	Organic acids	0	0	0	0	0	0	0	1
Acetyl-CoA acetyltransferase (EC 2.3.1.9)	Carbohydrates	One-carbon Metabolism	0	0	0	0	0	0	1	0
Acetyl-CoA:acetoacetyl-CoA transferase, alpha subunit (EC 2.8.3.8)	Carbohydrates	Fermentation	0	0	0	0	1	0	1	0
Acetyl-coenzyme A synthetase (EC 6.2.1.1)	Carbohydrates	Central carbohydrate meta	1	0	0	0	1	0	1	0
acetyltransferase, GNAT family	Carbohydrates	Central carbohydrate meta	0	0	0	0	1	0	0	0
Aconitate hydratase (EC 4.2.1.3)	Carbohydrates	Central carbohydrate meta	0	0	0	0	1	0	1	0
Aconitate hydratase 2 (EC 4.2.1.3)	Carbohydrates	Central carbohydrate meta	0	0	0	0	0	0	1	0
Aerobic glycerol-3-phosphate dehydrogenase (EC 1.1.5.3); GlpD	Carbohydrates	Sugar alcohols	1	0	1	0	0	0	0	0
Alanine racemase (EC 5.1.1.1)##catabolic	Carbohydrates	Central carbohydrate meta	1	0	0	0	1	0	1	0
Alcohol dehydrogenase (EC 1.1.1.1)	Carbohydrates	Fermentation	0	0	0	0	1	0	1	0
Aldehyde dehydrogenase (EC 1.2.1.3); patD?	Carbohydrates	Central carbohydrate meta	1	0	0	0	0	0	1	0
Aldehyde dehydrogenase A (EC 1.2.1.22); gabD	Carbohydrates	Central carbohydrate meta	0	0	0	0	1	0	1	0
Aldehyde dehydrogenase B (EC 1.2.1.22)	Carbohydrates	Central carbohydrate meta	0	0	0	0	1	0	1	0
Aldo-keto reductase	Carbohydrates	Monosaccharides	0	0	0	0	0	0	1	0
Aldose 1-epimerase (EC 5.1.3.3)	Carbohydrates	Di- and oligosaccharides	0	1	0	0	0	1	0	1
Alpha,alpha-trehalose-phosphate synthase [UDP-forming] (EC 2.4.1.15)	Carbohydrates	Di- and oligosaccharides	0	0	0	0	0	0	1	0
Alpha-acetolactate decarboxylase (EC 4.1.1.5)	Carbohydrates	Organic acids	0	0	0	0	0	0	0	1
Alpha-galactosidase (EC 3.2.1.22)	Carbohydrates	Di- and oligosaccharides	0	0	0	0	1	0	1	0
Arabinose-proton symporter	Carbohydrates	Monosaccharides	0	1	0	0	0	1	0	1
Ascorbate-specific PTS system, EIIC component	Carbohydrates	Monosaccharides	0	0	0	0	1	0	1	0
ATP:Cob(I)alamin adenosyltransferase (EC 2.5.1.17), ethanolamine utilization	Carbohydrates	Sugar alcohols	0	0	0	0	0	0	1	0
Beta-galactosidase (EC 3.2.1.23)	Carbohydrates	Di- and oligosaccharides	0	0	0	0	0	0	0	1
Beta-glucosidase (EC 3.2.1.21)	Carbohydrates	Di- and oligosaccharides	0	0	0	0	1	0	1	0
Beta-mannosidase (EC 3.2.1.25)	Carbohydrates	Monosaccharides	0	0	0	0	0	0	0	1
Chitinase (EC 3.2.1.14)	Carbohydrates	Aminosugars	0	0	0	0	0	0	1	0

Chromosomal Genes (Plasmid-borne genes listed separately below)	SEED CATEGORY	SEED SUBCATEGORY	BR7-2h-up	BR7-2h-down	BR21-2h-up	BR21-2h-down	BR7-8h-up	BR7-8h-down	BR21-8h-up	BR21-8h-down
Citrate lyase alpha chain (EC 4.1.3.6)	Carbohydrates	Organic acids	0	0	0	0	1	0	0	0
Citrate lyase beta chain (EC 4.1.3.6)	Carbohydrates	Organic acids	0	0	0	0	1	0	0	0
Citrate synthase (si) (EC 2.3.3.1)	Carbohydrates	Central carbohydrate meta	0	0	0	0	1	0	1	0
Cytoplasmic alpha-amylase (EC 3.2.1.1)	Carbohydrates	Di- and oligosaccharides	0	0	0	0	1	0	1	0
D-amino acid dehydrogenase small subunit (EC 1.4.99.1)	Carbohydrates	Central carbohydrate meta	1	0	0	0	1	0	1	0
D-arabinitol 4-dehydrogenase (EC 1.1.1.11)	Carbohydrates	Sugar alcohols	0	0	0	0	1	0	1	0
D-galactarate dehydratase (EC 4.2.1.42)	Carbohydrates	Monosaccharides	0	1	0	0	0	1	0	0
D-glucarate permease	Carbohydrates	Monosaccharides	0	1	0	0	0	1	0	0
Dihydrolipoamide acetyltransferase component of pyruvate dehydrogenase comp	Carbohydrates	Central carbohydrate meta	0	0	0	0	0	0	0	1
Dihydrolipoamide succinyltransferase component (E2) of 2-oxoglutarate dehydrog	Carbohydrates	Central carbohydrate meta	0	0	0	0	0	0	1	0
D-lactate dehydrogenase (EC 1.1.1.28)	Carbohydrates	Fermentation	0	0	0	0	0	1	0	1
D-ribulokinase (EC 2.7.1.47)	Carbohydrates	Central carbohydrate meta	0	0	0	0	0	0	1	0
D-xylose transport ATP-binding protein XylG	Carbohydrates	Monosaccharides	0	0	0	0	1	0	1	0
Endo-1,4-beta-xylanase A precursor (EC 3.2.1.8)	Carbohydrates	Monosaccharides	0	0	0	0	1	0	1	0
Endo-b1,4-mannanase 5C	Carbohydrates	Central carbohydrate meta	0	0	0	0	0	0	1	0
Enolase (EC 4.2.1.11)	Carbohydrates	Central carbohydrate meta	0	0	0	0	0	0	0	1
Enoyl-CoA hydratase (EC 4.2.1.17)	Carbohydrates	Fermentation	1	0	0	0	1	0	1	0
Epi-inositol hydrolase (EC 3.7.1.-)	Carbohydrates	Sugar alcohols	0	0	0	0	0	0	1	0
Epimerase KguE	Carbohydrates	Monosaccharides	0	0	0	0	1	0	0	0
Ethanolamine ammonia-lyase heavy chain (EC 4.3.1.7)	Carbohydrates	Sugar alcohols	0	0	0	0	1	0	1	0
Ethanolamine ammonia-lyase light chain (EC 4.3.1.7)	Carbohydrates	Sugar alcohols	0	0	0	0	0	0	1	0
Ethanolamine operon regulatory protein	Carbohydrates	Sugar alcohols	0	0	0	0	0	0	1	0
Ethanolamine permease	Carbohydrates	Sugar alcohols	0	0	0	0	0	0	1	0
Ethanolamine utilization polyhedral-body-like protein EutL	Carbohydrates	Sugar alcohols	0	0	0	0	0	0	1	0
Formate efflux transporter (TC 2.A.44 family)	Carbohydrates	Fermentation	0	1	0	0	0	1	0	1
Fructose-bisphosphate aldolase class I (EC 4.1.2.13)	Carbohydrates	Central carbohydrate meta	0	0	0	0	1	0	1	0
Fumarate hydratase class I, aerobic (EC 4.2.1.2)	Carbohydrates	Central carbohydrate meta	0	0	0	0	1	0	1	0
Fumarate hydratase class I, anaerobic (EC 4.2.1.2)	Carbohydrates	One-carbon Metabolism	0	0	1	0	0	0	1	0
Fumarate hydratase class II (EC 4.2.1.2)	Carbohydrates	Central carbohydrate meta	0	0	0	0	1	0	1	0
Galactitol utilization operon repressor	Carbohydrates	Monosaccharides	0	0	0	0	0	0	0	1
Galactokinase (EC 2.7.1.6)	Carbohydrates	Di- and oligosaccharides	0	1	0	0	0	1	0	1
Galactose/methyl galactoside ABC transport system, ATP-binding protein MglA (EC	Carbohydrates	Di- and oligosaccharides	0	0	0	0	1	0	1	0
Galactose/methyl galactoside ABC transport system, D-galactose-binding periplasn	Carbohydrates	Di- and oligosaccharides	0	0	0	0	0	0	1	0
Galactose/methyl galactoside ABC transport system, permease protein MglC (TC 3	Carbohydrates	Di- and oligosaccharides	0	0	1	0	0	0	1	0
Galactose-1-phosphate uridylyltransferase (EC 2.7.7.10)	Carbohydrates	Di- and oligosaccharides	0	1	0	0	0	1	0	1
Glucokinase, ROK family (EC 2.7.1.2)	Carbohydrates	Di- and oligosaccharides	0	0	0	0	0	0	1	0
Gluconate 2-dehydrogenase (EC 1.1.99.3), membrane-bound, gamma subunit	Carbohydrates	Monosaccharides	0	0	0	0	1	0	0	0
Gluconate permease	Carbohydrates	Monosaccharides	0	0	0	0	0	0	1	0
Glucose-1-phosphatase (EC 3.1.3.10)	Carbohydrates	Polysaccharides	0	0	0	0	0	0	1	0
Glucose-1-phosphate adenylyltransferase (EC 2.7.7.27)	Carbohydrates	Polysaccharides	0	0	0	0	0	0	1	0
Glucose-6-phosphate isomerase (EC 5.3.1.9);pgi	Carbohydrates	Central carbohydrate meta	0	0	0	0	0	0	0	1
Glyceraldehyde-3-phosphate ketol-isomerase (EC 5.3.1.1)	Carbohydrates	Sugar alcohols	0	0	0	0	0	0	1	0
Glycerol kinase (EC 2.7.1.30); GlpK	Carbohydrates	Sugar alcohols	0	0	1	0	0	0	1	0
Glycerol-3-phosphate ABC transporter, ATP-binding protein UgpC (TC 3.A.1.1.3)	Carbohydrates	Sugar alcohols	1	0	0	0	1	0	1	0
Glycerol-3-phosphate ABC transporter, periplasmic glycerol-3-phosphate-binding p	Carbohydrates	Sugar alcohols	1	0	0	0	1	0	1	0
Glycerol-3-phosphate ABC transporter, permease protein UgpA (TC 3.A.1.1.3)	Carbohydrates	Sugar alcohols	1	0	0	0	1	0	1	0
Glycerol-3-phosphate ABC transporter, permease protein UgpE (TC 3.A.1.1.3)	Carbohydrates	Sugar alcohols	1	0	0	0	1	0	1	0
Glycerol-3-phosphate transporter; GlpT	Carbohydrates	Sugar alcohols	1	0	1	0	1	0	1	0
Glycerophosphoryl diester phosphodiesterase, periplasmic (EC 3.1.4.46); GlpQ	Carbohydrates	Sugar alcohols	1	0	1	0	1	0	1	0
Glycogen debranching enzyme (EC 3.2.1.-)	Carbohydrates	Di- and oligosaccharides	0	0	0	0	0	0	1	0

Chromosomal Genes (Plasmid-borne genes listed separately below)	SEED CATEGORY	SEED SUBCATEGORY	BR7-2h-up	BR7-2h-down	BR21-2h-up	BR21-2h-down	BR7-8h-up	BR7-8h-down	BR21-8h-up	BR21-8h-down
Glycogen phosphorylase (EC 2.4.1.1)	Carbohydrates	Di- and oligosaccharides	0	0	0	0	1	0	1	0
Glycogen synthase, ADP-glucose transglucosylase (EC 2.4.1.21)	Carbohydrates	Polysaccharides	0	0	0	0	1	0	1	0
Glycolate dehydrogenase (EC 1.1.99.14), subunit GlcD	Carbohydrates	Central carbohydrate meta	0	0	0	0	0	0	1	0
Glyoxalase family protein	Carbohydrates	Monosaccharides	1	0	0	0	1	0	1	0
Hexose phosphate uptake regulatory protein UhpC	Carbohydrates	Monosaccharides	0	0	0	0	0	1	0	0
High-affinity gluconate transporter GntT	Carbohydrates	Monosaccharides	0	0	1	0	0	0	1	0
Hydroxypyruvate isomerase (EC 5.3.1.22)	Carbohydrates	Organic acids	0	0	0	0	1	0	1	0
Inositol transport system ATP-binding protein	Carbohydrates	Sugar alcohols	1	0	0	0	1	0	1	0
Inositol transport system permease protein	Carbohydrates	Sugar alcohols	1	0	0	0	1	0	1	0
Inositol transport system sugar-binding protein	Carbohydrates	Sugar alcohols	0	0	0	0	1	0	1	0
Inosose dehydratase (EC 4.2.1.44)	Carbohydrates	Sugar alcohols	0	0	0	0	0	0	1	0
Inosose isomerase (EC 5.3.99.-)	Carbohydrates	Sugar alcohols	0	0	0	0	0	0	1	0
Isocitrate dehydrogenase phosphatase (EC 2.7.11.5)/kinase (EC 3.1.3.-)	Carbohydrates	Central carbohydrate meta	0	0	0	0	1	0	1	0
Isocitrate lyase (EC 4.1.3.1)	Carbohydrates	Central carbohydrate meta	0	0	0	0	1	0	1	0
Lactate-responsive regulator LldR in Enterobacteria, GntR family	Carbohydrates	Organic acids	1	0	0	0	1	0	1	0
Lactose permease	Carbohydrates	Di- and oligosaccharides	0	0	0	0	1	0	0	0
Lactoylglutathione lyase and related lyases	Carbohydrates	Monosaccharides	0	0	0	0	0	0	1	0
L-arabinose isomerase (EC 5.3.1.4)	Carbohydrates	Monosaccharides	0	0	0	0	0	0	1	0
L-arabinose transport ATP-binding protein AraG (TC 3.A.1.2.2)	Carbohydrates	Monosaccharides	0	0	0	0	0	0	1	0
L-arabinose transport system permease protein (TC 3.A.1.2.2)	Carbohydrates	Monosaccharides	0	0	0	0	0	0	1	0
L-arabinose-binding periplasmic protein precursor AraF (TC 3.A.1.2.2)	Carbohydrates	Monosaccharides	0	0	0	0	0	0	1	0
L-fucose isomerase (EC 5.3.1.25)	Carbohydrates	Monosaccharides	0	0	0	0	0	0	1	0
L-fucose operon activator	Carbohydrates	Monosaccharides	0	0	0	0	0	0	1	0
L-fuculokinase (EC 2.7.1.51)	Carbohydrates	Monosaccharides	0	0	0	0	0	0	1	0
L-lactate permease	Carbohydrates	Organic acids	1	0	0	0	1	0	1	0
Low-affinity gluconate/H+ symporter GntU	Carbohydrates	Monosaccharides	0	0	0	0	0	1	0	0
L-rhamnonate dehydratase (EC 4.2.1.90)	Carbohydrates	Carbohydrates - no subcat	0	0	0	0	0	0	1	0
L-rhamnonate transporter (predicted by genome context)	Carbohydrates	Carbohydrates - no subcat	0	0	0	0	1	0	1	0
Malate dehydrogenase (EC 1.1.1.37)	Carbohydrates	Central carbohydrate meta	0	0	0	0	1	0	1	0
Malate Na(+) symporter	Carbohydrates	Central carbohydrate meta	0	0	0	0	0	0	1	0
Malate synthase (EC 2.3.3.9)	Carbohydrates	Central carbohydrate meta	0	0	0	0	1	0	1	0
Malate:quinone oxidoreductase (EC 1.1.5.4)	Carbohydrates	Central carbohydrate meta	1	0	0	0	1	0	1	0
Maltodextrin glucosidase (EC 3.2.1.20)	Carbohydrates	Di- and oligosaccharides	0	0	0	0	0	0	0	1
Maltodextrin phosphorylase (EC 2.4.1.1)	Carbohydrates	Di- and oligosaccharides	0	0	0	0	0	0	0	1
Maltoporin (maltose/maltodextrin high-affinity receptor, phage lambda receptor p	Carbohydrates	Di- and oligosaccharides	0	0	0	0	1	0	0	1
Maltose operon periplasmic protein MalM	Carbohydrates	Di- and oligosaccharides	0	0	0	0	1	0	0	1
Maltose/maltodextrin ABC transporter, permease protein MalF	Carbohydrates	Di- and oligosaccharides	0	0	0	0	0	0	0	1
Maltose/maltodextrin ABC transporter, permease protein MalG	Carbohydrates	Di- and oligosaccharides	0	0	0	0	1	0	0	1
Maltose/maltodextrin ABC transporter, substrate binding periplasmic protein MalE	Carbohydrates	Di- and oligosaccharides	0	0	0	0	1	0	0	1
Maltose/maltodextrin transport ATP-binding protein MalK (EC 3.6.3.19)	Carbohydrates	Di- and oligosaccharides	0	0	0	0	1	0	0	1
Maltose-6'-phosphate glucosidase (EC 3.2.1.122)	Carbohydrates	Di- and oligosaccharides	0	0	0	0	1	0	1	0
Melibiose carrier protein, Na+/melibiose symporter	Carbohydrates	Di- and oligosaccharides	0	0	0	0	0	0	1	0
Methylglyoxal reductase, acetol producing (EC 1.1.1.-) / 2,5-diketo-D-gluconate re	Carbohydrates	Central carbohydrate meta	0	0	0	0	1	0	1	0
Methylglyoxal synthase (EC 4.2.3.3)	Carbohydrates	Central carbohydrate meta	0	0	0	0	0	0	0	1
Mgl repressor and galactose ultrainduction factor GalS, HTH-type transcriptional re	Carbohydrates	Di- and oligosaccharides	1	0	0	0	0	0	1	0
Mlc, transcriptional repressor of MalT (the transcriptional activator of maltose reg	Carbohydrates	Di- and oligosaccharides	0	0	0	0	0	0	0	1
Myo-inositol 2-dehydrogenase 1 (EC 1.1.1.18)	Carbohydrates	Sugar alcohols	0	0	0	0	0	0	1	0
NAD-dependent glyceraldehyde-3-phosphate dehydrogenase (EC 1.2.1.12)	Carbohydrates	Central carbohydrate meta	0	0	0	0	0	0	0	1
NADP-dependent malic enzyme (EC 1.1.1.40)	Carbohydrates	Central carbohydrate meta	0	0	0	0	0	0	1	0
N-hydroxyarylamine O-acetyltransferase (EC 2.3.1.118)	Carbohydrates	Central carbohydrate meta	0	0	0	0	0	0	1	0

Chromosomal Genes (Plasmid-borne genes listed separately below)	SEED CATEGORY	SEED SUBCATEGORY	BR7-2h-up	BR7-2h-down	BR21-2h-up	BR21-2h-down	BR7-8h-up	BR7-8h-down	BR21-8h-up	BR21-8h-down
Oxidoreductase	Carbohydrates	Central carbohydrate meta	0	0	0	0	0	0	1	0
Peptide methionine sulfoxide reductase MsrB (EC 1.8.4.12)	Carbohydrates	Carbohydrates - no subcat	1	0	0	0	1	0	1	0
Periplasmic alpha-amylase (EC 3.2.1.1)	Carbohydrates	Di- and oligosaccharides	0	0	0	0	1	0	0	1
Periplasmic beta-glucosidase (EC 3.2.1.21)	Carbohydrates	Di- and oligosaccharides	0	0	0	0	0	0	1	0
Phenylacetate degradation enoyl-CoA hydratase PaaB (EC 4.2.1.17)	Carbohydrates	Central carbohydrate meta	0	0	0	0	1	0	0	0
Phenylacetate-CoA oxygenase, PaaG subunit	Carbohydrates	Central carbohydrate meta	0	0	0	0	1	0	1	0
Phenylacetate-CoA oxygenase, PaaI subunit	Carbohydrates	Central carbohydrate meta	0	0	0	0	1	0	0	0
Phenylacetate-coenzyme A ligase (EC 6.2.1.30) PaaF	Carbohydrates	Central carbohydrate meta	0	0	0	0	1	0	1	0
Phosphatase NagD predicted to act in N-acetylglucosamine utilization subsystem	Carbohydrates	Aminosugars	0	0	0	0	0	0	0	1
Phosphate acetyltransferase (EC 2.3.1.8); pta	Carbohydrates	Central carbohydrate meta	0	0	0	0	0	0	0	1
Phosphoenolpyruvate carboxykinase [ATP] (EC 4.1.1.49)	Carbohydrates	Central carbohydrate meta	1	0	0	0	1	0	1	0
Phosphoenolpyruvate carboxylase (EC 4.1.1.31)	Carbohydrates	Central carbohydrate meta	0	0	0	0	0	0	0	1
Phosphoenolpyruvate synthase (EC 2.7.9.2)	Carbohydrates	Central carbohydrate meta	0	0	0	0	0	0	1	0
Phosphogluconate repressor HexR, RpiR family	Carbohydrates	Monosaccharides	0	0	0	0	0	1	0	0
Phosphoglycerate kinase (EC 2.7.2.3)	Carbohydrates	Central carbohydrate meta	0	0	0	0	0	0	0	1
Phosphosugar-binding transcriptional regulator, RpiR family	Carbohydrates	Central carbohydrate meta	0	0	0	0	0	0	0	1
Probable carboxyvinyl-carboxyphosphonate phosphorylmutase (EC 2.7.8.23)	Carbohydrates	Central carbohydrate meta	0	0	0	0	0	0	0	1
PTS system, arbutin-, cellobiose-, and salicin-specific IIBC component (EC 2.7.1.69)	Carbohydrates	Di- and oligosaccharides	0	0	0	0	1	0	1	0
PTS system, cellobiose-specific IIA component (EC 2.7.1.69)	Carbohydrates	Di- and oligosaccharides	0	0	0	0	0	0	1	0
PTS system, fructose-specific IIBC component (EC 2.7.1.69)	Carbohydrates	Monosaccharides	0	0	1	0	0	0	0	0
PTS system, fructose-specific phosphocarrier protein HPr/ IIA component (EC 2.7.1	Carbohydrates	Monosaccharides	0	0	1	0	0	0	0	0
PTS system, galactitol-specific IIA component (EC 2.7.1.69)	Carbohydrates	Monosaccharides	0	0	0	0	1	0	0	0
PTS system, glucitol/sorbitol-specific IIA component (EC 2.7.1.69)	Carbohydrates	Monosaccharides	0	0	0	0	0	0	1	0
PTS system, glucitol/sorbitol-specific IIB component and second of two IIC compor	Carbohydrates	Monosaccharides	0	0	0	0	0	0	1	0
PTS system, glucitol/sorbitol-specific IIC component (EC 2.7.1.69)	Carbohydrates	Monosaccharides	0	0	0	1	0	0	1	0
PTS system, maltose and glucose-specific IIC component (EC 2.7.1.69)	Carbohydrates	Di- and oligosaccharides	0	0	0	0	0	0	1	0
PTS system, mannitol-specific IIC component (EC 2.7.1.69)	Carbohydrates	Sugar alcohols	0	0	0	0	1	0	1	0
PTS system, mannose-specific IIB component (EC 2.7.1.69)	Carbohydrates	Monosaccharides	0	0	0	0	0	0	1	0
PTS system, mannose-specific IIC component (EC 2.7.1.69)	Carbohydrates	Monosaccharides	0	0	0	0	0	0	1	0
PTS system, mannose-specific IID component (EC 2.7.1.69)	Carbohydrates	Monosaccharides	0	0	0	0	0	0	1	0
PTS system, trehalose-specific IIB component (EC 2.7.1.69)	Carbohydrates	Di- and oligosaccharides	0	0	0	0	0	0	0	1
Putative membrane protein, clustering with ActP	Carbohydrates	Central carbohydrate meta	1	0	1	0	1	0	1	0
Putative oxidoreductase YdjL	Carbohydrates	Carbohydrates - no subcat	0	0	0	0	1	0	1	0
putative protein PaaI, possibly involved in aromatic compounds catabolism	Carbohydrates	Central carbohydrate meta	1	0	0	0	1	0	1	0
Putative rpiR-family transcriptional regulatory protein	Carbohydrates	Central carbohydrate meta	0	1	0	0	0	0	0	0
Putative sugar ABC transporter precursor	Carbohydrates	Monosaccharides	0	1	0	0	0	0	0	0
putative sugar phosphotransferase component II B	Carbohydrates	Monosaccharides	0	0	0	0	0	0	1	0
Pyruvate decarboxylase (EC 4.1.1.1)	Carbohydrates	Central carbohydrate meta	0	0	0	0	1	0	1	0
Pyruvate formate-lyase (EC 2.3.1.54)	Carbohydrates	Fermentation	0	0	0	0	0	0	0	1
Pyruvate kinase (EC 2.7.1.40)	Carbohydrates	Central carbohydrate meta	0	0	0	0	0	0	0	1
Pyruvate oxidase [ubiquinone, cytochrome] (EC 1.2.2.2); homolog poxB	Carbohydrates	Central carbohydrate meta	0	0	0	0	0	0	1	0
Rhamnogalacturonides degradation protein RhiN	Carbohydrates	Monosaccharides	0	0	0	0	0	0	1	0
Ribitol 2-dehydrogenase (EC 1.1.1.56)	Carbohydrates	Central carbohydrate meta	0	0	0	0	0	0	1	0
Ribitol/Xylitol/Arabitol transporter, MFS superfamily	Carbohydrates	Sugar alcohols	0	0	1	0	0	0	1	0
Ribose ABC transport system, periplasmic ribose-binding protein RbsB (TC 3.A.1.2.:	Carbohydrates	Monosaccharides	0	0	1	0	1	0	1	0
Ribose/xylose/arabinose/galactoside ABC-type transport systems, periplasmic suga	Carbohydrates	Monosaccharides	0	0	0	0	1	0	1	0
Ribose/xylose/arabinose/galactoside ABC-type transport systems, permease comp	Carbohydrates	Monosaccharides	0	0	0	0	1	0	0	0
ribosomal-protein-alanine N-acetyltransferase	Carbohydrates	Central carbohydrate meta	0	0	0	0	0	0	1	0
Ribulokinase (EC 2.7.1.16)	Carbohydrates	Monosaccharides	0	0	0	0	1	0	0	0
Sensor histidine protein kinase UhpB, glucose-6-phosphate specific (EC 2.7.13.3)	Carbohydrates	Monosaccharides	0	0	0	0	0	1	0	0

Chromosomal Genes (Plasmid-borne genes listed separately below)	SEED CATEGORY	SEED SUBCATEGORY	BR7-2h-up	BR7-2h-down	BR21-2h-up	BR21-2h-down	BR7-8h-up	BR7-8h-down	BR21-8h-up	BR21-8h-down
Sensor kinase CitA, DpiB (EC 2.7.3.-)	Carbohydrates	Organic acids	0	0	0	0	1	0	1	0
Serine protein kinase (prkA protein), P-loop containing	Carbohydrates	Carbohydrates - no subcat	0	0	0	0	1	0	1	0
Sodium:dicarboxylate symporter	Carbohydrates	Central carbohydrate meta	0	0	0	0	1	0	0	0
Succinate dehydrogenase iron-sulfur protein (EC 1.3.99.1)	Carbohydrates	Central carbohydrate meta	1	0	0	0	1	0	1	0
Succinyl-CoA ligase [ADP-forming] alpha chain (EC 6.2.1.5)	Carbohydrates	Central carbohydrate meta	0	0	1	0	0	0	1	0
Succinyl-CoA ligase [ADP-forming] beta chain (EC 6.2.1.5)	Carbohydrates	Central carbohydrate meta	0	0	0	0	0	0	1	0
Succinyl-CoA:3-ketoacid-coenzyme A transferase subunit A (EC 2.8.3.5)	Carbohydrates	One-carbon Metabolism	0	0	0	0	1	0	0	0
Sucrose permease, major facilitator superfamily	Carbohydrates	Di- and oligosaccharides	0	0	0	0	0	0	1	0
Sugar diacid utilization regulator SdaR	Carbohydrates	Monosaccharides	0	1	0	0	0	1	0	0
Thiamine pyrophosphate-requiring protein Saci 2281	Carbohydrates	Central carbohydrate meta	0	0	0	0	0	0	1	0
Threonine kinase in B12 biosynthesis	Carbohydrates	Sugar alcohols	0	0	0	0	0	0	0	1
transcriptional regulator, RpiR family	Carbohydrates	Sugar alcohols	0	0	0	0	0	0	0	1
Transcriptional regulatory protein CitB, DpiA	Carbohydrates	Organic acids	0	0	0	0	1	0	1	0
Transcriptional repressor for pyruvate dehydrogenase complex	Carbohydrates	Central carbohydrate meta	0	0	0	0	0	1	0	1
Transcriptional repressor of the fructose operon, DeoR family	Carbohydrates	Monosaccharides	0	0	0	0	0	0	1	0
Transcriptional repressor of the lac operon	Carbohydrates	Di- and oligosaccharides	0	0	0	0	0	0	0	1
Trehalase (EC 3.2.1.28); Periplasmic trehalase precursor (EC 3.2.1.28)	Carbohydrates	Di- and oligosaccharides	0	0	0	0	1	0	1	0
Trehalose synthase (EC 5.4.99.16)	Carbohydrates	Di- and oligosaccharides	0	0	0	0	0	0	1	0
Trehalose-6-phosphate hydrolase (EC 3.2.1.93)	Carbohydrates	Di- and oligosaccharides	0	0	0	0	0	0	0	1
Trehalose-6-phosphate phosphatase (EC 3.1.3.12)	Carbohydrates	Di- and oligosaccharides	0	0	0	0	0	0	1	0
Triosephosphate isomerase (EC 5.3.1.1)	Carbohydrates	Central carbohydrate meta	0	0	0	0	0	0	0	1
UDP-glucose 4-epimerase (EC 5.1.3.2)	Carbohydrates	Di- and oligosaccharides	0	1	0	0	0	1	0	1
UDP-sugar hydrolase (EC 3.6.1.45); 5'-nucleotidase (EC 3.1.3.5)	Carbohydrates	Central carbohydrate meta	0	0	0	0	1	0	0	0
Uncharacterized protein YeaC	Carbohydrates	Carbohydrates - no subcat	0	0	0	0	1	0	0	0
UPF0229 protein YeaH	Carbohydrates	Carbohydrates - no subcat	0	0	0	0	1	0	1	0
Xylose ABC transporter, periplasmic xylose-binding protein XylF	Carbohydrates	Monosaccharides	0	0	0	0	1	0	1	0
Xylose isomerase (EC 5.3.1.5)	Carbohydrates	Monosaccharides	0	0	0	0	1	0	1	0
Xyloside transporter XynT	Carbohydrates	Monosaccharides	0	0	0	0	0	0	1	0
Xylulose kinase (EC 2.7.1.17)	Carbohydrates	Monosaccharides	0	0	0	0	0	0	1	0
Mannitol-1-phosphate 5-dehydrogenase (EC 1.1.1.17)	Carbohydrates - stress	Sugar alcohols	0	0	0	0	1	0	1	0
Sorbitol-6-phosphate 2-dehydrogenase (EC 1.1.1.140)	Carbohydrates-stress	Monosaccharides	0	0	0	0	0	0	1	0
Carbamoyl-phosphate synthase large chain (EC 6.3.5.5)	Cell Division and Cell Cycle	Cell Division and Cell Cycle	0	0	0	0	0	0	0	1
Cell division transporter, ATP-binding protein FtsE (TC 3.A.5.1.1)	Cell Division and Cell Cycle	Cell Division and Cell Cycle	0	0	0	0	0	0	0	1
Cell filamentation protein fic	Cell Division and Cell Cycle	Cell Division and Cell Cycle	0	0	0	0	0	0	1	0
Chromosome partitioning ATPase in PFGI-1-like cluster, ParA-like	Cell Division and Cell Cycle	Cell Division and Cell Cycle	0	0	0	0	0	0	0	1
Cyclopropane-fatty-acyl-phospholipid synthase (EC 2.1.1.79), plant type	Cell Division and Cell Cycle	Cell Division and Cell Cycle	0	0	0	1	0	0	0	0
DNA gyrase inhibitory protein	Cell Division and Cell Cycle	Cell Division and Cell Cycle	0	0	0	0	0	0	1	0
GTPase (EC 3.6.1.-)	Cell Division and Cell Cycle	Cell Division and Cell Cycle	0	0	0	0	0	0	1	0
Guanosine-5'-triphosphate,3'-diphosphate pyrophosphatase (EC 3.6.1.40) @ Exopo	Cell Division and Cell Cycle	Cell Division and Cell Cycle	0	0	0	0	0	0	0	1
Lipoprotein nlpI precursor	Cell Division and Cell Cycle	Cell Division and Cell Cycle	0	0	0	1	0	0	0	1
Preprotein translocase secY subunit (TC 3.A.5.1.1)	Cell Division and Cell Cycle	Cell Division and Cell Cycle	0	0	0	0	0	0	0	1
Rod shape-determining protein MreD	Cell Division and Cell Cycle	Cell Division and Cell Cycle	0	0	0	0	0	0	0	1
3-deoxy-D-manno-octulosonic-acid transferase (EC 2.-.-.-)	Cell Wall and Capsule	Gram-Negative cell wall co	0	0	0	0	0	1	0	0
ADP-heptose--lipooligosaccharide heptosyltransferase II (EC 2.4.1.-)	Cell Wall and Capsule	Gram-Negative cell wall co	0	0	0	0	0	0	0	1
ADP-L-glycero-D-manno-heptose-6-epimerase (EC 5.1.3.20)	Cell Wall and Capsule	Gram-Negative cell wall co	0	0	0	0	0	0	0	1
alpha-dextrin endo-1,6-alpha-glucosidase precursor (pullulanase precursor)	Cell Wall and Capsule	Gram-Negative cell wall co	0	0	1	0	1	0	0	0
AmpG permease	Cell Wall and Capsule	Cell Wall and Capsule - no s	0	0	0	0	0	0	0	1
Cellulose synthase operon protein C	Cell Wall and Capsule	Gram-Negative cell wall co	0	0	0	0	0	0	1	0
Cellulose synthase, putative	Cell Wall and Capsule	Gram-Negative cell wall co	0	0	0	0	0	0	1	0
Cyclic di-GMP binding protein precursor	Cell Wall and Capsule	Capsular and extracellular	0	0	0	0	0	0	1	0

Chromosomal Genes (Plasmid-borne genes listed separately below)	SEED CATEGORY	SEED SUBCATEGORY	BR7-2h-up	BR7-2h-down	BR21-2h-up	BR21-2h-down	BR7-8h-up	BR7-8h-down	BR21-8h-up	BR21-8h-down
D-alanine--D-alanine ligase A (EC 6.3.2.4)	Cell Wall and Capsule	Cell Wall and Capsule - no s	0	0	0	0	0	0	0	1
D-glycero-D-manno-heptose 1,7-bisphosphate phosphatase (EC 3.1.1.-)	Cell Wall and Capsule	Capsular and extracellular	0	0	0	0	0	0	0	1
dTDP-4-dehydrorhamnose 3,5-epimerase (EC 5.1.3.13)	Cell Wall and Capsule	Capsular and extracellular	0	0	0	1	0	0	0	0
Endoglucanase precursor (EC 3.2.1.4)	Cell Wall and Capsule	Gram-Negative cell wall co	0	0	0	0	0	0	1	0
Fe(2+)/alpha-ketoglutarate-dependent dioxygenase LpxO	Cell Wall and Capsule	Gram-Negative cell wall co	1	0	0	0	1	0	1	0
Glycoprotein-polysaccharide metabolism	Cell Wall and Capsule	Gram-Negative cell wall co	0	0	0	0	0	0	1	0
Inner membrane protein YrbG, predicted calcium/sodium:proton antiporter	Cell Wall and Capsule	Gram-Negative cell wall co	0	0	0	0	0	0	0	1
L,D-transpeptidase ErfK	Cell Wall and Capsule	Cell Wall and Capsule - no s	0	0	0	0	1	0	1	0
L,D-transpeptidase YcfS	Cell Wall and Capsule	Cell Wall and Capsule - no s	0	0	0	0	0	0	1	0
L,D-transpeptidase YnhG	Cell Wall and Capsule	Cell Wall and Capsule - no s	0	0	0	0	1	0	1	0
Lipid A biosynthesis lauroyl acyltransferase (EC 2.3.1.-)	Cell Wall and Capsule	Gram-Negative cell wall co	0	0	0	0	0	1	0	1
Lipopolysaccharide biosynthesis protein RffA	Cell Wall and Capsule	Gram-Negative cell wall co	0	0	0	0	0	0	0	1
Lipopolysaccharide biosynthesis protein RffC	Cell Wall and Capsule	Gram-Negative cell wall co	0	0	0	0	0	0	0	1
Lipoprotein NlpD	Cell Wall and Capsule	Gram-Negative cell wall co	0	0	0	0	0	0	1	0
Lipoprotein spr precursor	Cell Wall and Capsule	Cell Wall and Capsule - no s	0	0	0	0	0	0	1	0
N-acetylglucosamine-1-phosphate uridyltransferase (EC 2.7.7.23) / Glucosamine-1	Cell Wall and Capsule	Gram-Negative cell wall co	0	0	0	0	0	0	0	1
N-acetylmuramic acid 6-phosphate etherase	Cell Wall and Capsule	Gram-Negative cell wall co	0	0	0	0	0	1	0	0
Outer membrane chaperone Skp (OmpH) precursor	Cell Wall and Capsule	Gram-Negative cell wall co	0	0	0	0	0	0	0	1
Oxidoreductase, short-chain dehydrogenase/reductase family (EC 1.1.1.-)	Cell Wall and Capsule	Capsular and extracellular	0	1	0	1	0	0	0	1
Phosphoethanolamine transferase specific for the outer Kdo residue of lipopolysac	Cell Wall and Capsule	Gram-Negative cell wall co	0	0	0	0	0	0	0	1
probable exported protein STY0357; YafK (peptidoglycan biosynthesis)	Cell Wall and Capsule	Gram-Negative cell wall co	0	0	0	0	0	0	1	0
Probable lipoprotein nlpC precursor	Cell Wall and Capsule	Cell Wall and Capsule - no s	0	0	0	0	0	0	1	0
Probable UDP-N-acetyl-D-mannosaminuronic acid transferase (EC 2.4.1.-)	Cell Wall and Capsule	Cell Wall and Capsule - no s	0	0	0	0	0	0	0	1
Putative outer membrane protein	Cell Wall and Capsule	Gram-Negative cell wall co	0	0	0	0	0	1	0	0
Succinoglycan biosynthesis protein exoV	Cell Wall and Capsule	Gram-Negative cell wall co	0	0	0	0	0	0	0	1
Transglycosylase associated protein	Cell Wall and Capsule	Gram-Negative cell wall co	0	0	0	0	0	0	1	0
UDP-4-amino-4-deoxy-L-arabinose--oxoglutarate aminotransferase (EC 2.6.1.-)	Cell Wall and Capsule	Gram-Negative cell wall co	0	0	0	0	0	0	1	0
Uncharacterized protein YhjG	Cell Wall and Capsule	Gram-Negative cell wall co	0	0	0	0	1	0	1	0
Undecaprenyl-diphosphatase (EC 3.6.1.27)	Cell Wall and Capsule	Gram-Negative cell wall co	0	0	0	0	0	0	0	1
Undecaprenyl-phosphate N-acetylglucosaminyl 1-phosphate transferase (EC 2.7.8.	Cell Wall and Capsule	Gram-Negative cell wall co	0	0	0	0	0	0	0	1
1,4-dihydroxy-2-naphthoyl-CoA hydrolase (EC 3.1.2.28) in menaquinone biosynthe	Cofactors, Vitamins, Prosthetic (	Quinone cofactors	0	0	0	0	0	0	1	0
2-succinyl-5-enolpyruvyl-6-hydroxy-3- cyclohexene-1-carboxylic-acid synthase (EC	Cofactors, Vitamins, Prosthetic (	Quinone cofactors	0	0	0	0	0	0	0	1
2-succinyl-6-hydroxy-2,4-cyclohexadiene-1- carboxylate synthase (EC 4.2.99.20)	Cofactors, Vitamins, Prosthetic (	Quinone cofactors	0	0	0	0	0	0	0	1
3-ketoacyl-CoA thiolase (EC 2.3.1.16)	Cofactors, Vitamins, Prosthetic (	Biotin	1	0	0	0	1	0	1	0
3-ketoacyl-CoA thiolase (EC 2.3.1.16)	Cofactors, Vitamins, Prosthetic (	Biotin	0	0	0	0	1	0	1	0
4-hydroxythreonine-4-phosphate dehydrogenase (EC 1.1.1.262)	Cofactors, Vitamins, Prosthetic (	Pyridoxine	0	0	0	0	0	0	0	1
8-amino-7-oxononanoate synthase (EC 2.3.1.47)	Cofactors, Vitamins, Prosthetic (	Biotin	0	0	0	0	0	1	0	1
Adenosylmethionine-8-amino-7-oxononanoate aminotransferase (EC 2.6.1.62)	Cofactors, Vitamins, Prosthetic (	Biotin	0	0	0	0	0	1	0	1
ATPase component BioM of energizing module of biotin ECF transporter	Cofactors, Vitamins, Prosthetic (	Biotin	0	0	0	0	0	0	1	0
Biotin synthase (EC 2.8.1.6)	Cofactors, Vitamins, Prosthetic (	Biotin	0	0	0	0	0	1	0	0
Biotin synthesis protein BioC	Cofactors, Vitamins, Prosthetic (	Biotin	0	0	0	0	0	1	0	0
Coenzyme PQQ synthesis protein B	Cofactors, Vitamins, Prosthetic (	Quinone cofactors	0	0	0	0	0	0	1	0
Coenzyme PQQ synthesis protein C	Cofactors, Vitamins, Prosthetic (	Quinone cofactors	0	0	0	0	0	0	1	0
Coproporphyrinogen III oxidase, aerobic (EC 1.3.3.3)	Cofactors, Vitamins, Prosthetic (	Tetrapyrroles	0	0	0	0	0	1	0	0
C-terminal domain of CinA paralog, YdeJ	Cofactors, Vitamins, Prosthetic (	NAD and NADP	0	0	0	0	0	0	1	0
D-erythrose-4-phosphate dehydrogenase (EC 1.2.1.72)	Cofactors, Vitamins, Prosthetic (	Pyridoxine	0	0	0	0	0	0	0	1
Dethiobiotin synthetase (EC 6.3.3.3)	Cofactors, Vitamins, Prosthetic (	Biotin	0	0	0	0	0	0	0	1
Glutamate-1-semialdehyde aminotransferase (EC 5.4.3.8)	Cofactors, Vitamins, Prosthetic (	Tetrapyrroles	0	0	0	0	0	0	0	1
Hydroxyethylthiazole kinase (EC 2.7.1.50)	Cofactors, Vitamins, Prosthetic (	Cofactors, Vitamins, Prostl	0	0	0	0	1	0	1	0
Hydroxymethylpyrimidine ABC transporter, substrate-binding component	Cofactors, Vitamins, Prosthetic (	Cofactors, Vitamins, Prostl	0	0	0	0	1	0	1	0

Chromosomal Genes (Plasmid-borne genes listed separately below)	**SEED CATEGORY**	**SEED SUBCATEGORY**	BR7-2h-up	BR7-2h-down	BR21-2h-up	BR21-2h-down	BR7-8h-up	BR7-8h-down	BR21-8h-up	BR21-8h-down
Hydroxymethylpyrimidine ABC transporter, transmembrane component	Cofactors, Vitamins, Prosthetic (	Cofactors, Vitamins, Prosth	0	0	0	0	1	0	1	0
Hypothetical similar to thiamin biosynthesis lipoprotein ApbE	Cofactors, Vitamins, Prosthetic (	Cofactors, Vitamins, Prosth	0	0	0	0	0	0	1	0
Hypoxanthine-guanine phosphoribosyltransferase (EC 2.4.2.8)	Cofactors, Vitamins, Prosthetic (	Folate and pterines	0	0	0	0	0	0	0	1
Long-chain-fatty-acid--CoA ligase (EC 6.2.1.3)	Cofactors, Vitamins, Prosthetic (	Biotin	0	0	0	0	1	0	1	0
Microsomal dipeptidase (EC 3.4.13.19)	Cofactors, Vitamins, Prosthetic (	Quinone cofactors	0	0	0	0	0	0	1	0
Molybdenum-binding periplasmic protein	Cofactors, Vitamins, Prosthetic (	Folate and pterines	0	0	0	1	0	0	0	0
NAD(P)H-flavin reductase (EC 1.5.1.29) (EC 1.16.1.3)	Cofactors, Vitamins, Prosthetic (	Quinone cofactors	0	0	0	0	0	0	0	1
NfuA Fe-S protein maturation	Cofactors, Vitamins, Prosthetic (	Biotin	0	0	0	0	1	0	0	0
Nicotinamidase/isochorismatase family protein	Cofactors, Vitamins, Prosthetic (	NAD and NADP	0	0	1	0	0	0	1	0
Phosphomethylpyrimidine kinase (EC 2.7.4.7)	Cofactors, Vitamins, Prosthetic (	Cofactors, Vitamins, Prosth	0	0	0	0	1	0	1	0
Porphobilinogen synthase (EC 4.2.1.24)	Cofactors, Vitamins, Prosthetic (	Tetrapyrroles	0	0	0	0	0	0	0	1
Protoporphyrinogen IX oxidase, oxygen-independent, HemG (EC 1.3.-.-)	Cofactors, Vitamins, Prosthetic (	Tetrapyrroles	0	0	0	0	0	0	0	1
Putative molybdenum transport ATP-binding protein modF	Cofactors, Vitamins, Prosthetic (	Folate and pterines	0	1	0	1	0	1	0	1
Succinyl-CoA synthetase, alpha subunit-related enzymes	Cofactors, Vitamins, Prosthetic (	Quinone cofactors	0	0	0	0	0	0	1	0
Sulfur carrier protein adenylyltransferase ThiF	Cofactors, Vitamins, Prosthetic (	Cofactors, Vitamins, Prosth	0	0	0	0	1	0	1	0
Thiamin ABC transporter, substrate-binding component	Cofactors, Vitamins, Prosthetic (	Cofactors, Vitamins, Prosth	0	0	0	0	1	0	1	0
Thiaminase II (EC 3.5.99.2)	Cofactors, Vitamins, Prosthetic (	Cofactors, Vitamins, Prosth	0	0	0	0	1	0	1	0
Thiamin-phosphate pyrophosphorylase (EC 2.5.1.3)	Cofactors, Vitamins, Prosthetic (	Cofactors, Vitamins, Prosth	0	0	0	0	1	0	1	0
Thiazole biosynthesis protein ThiG	Cofactors, Vitamins, Prosthetic (	Cofactors, Vitamins, Prosth	0	0	0	0	1	0	1	0
Thiazole biosynthesis protein ThiH	Cofactors, Vitamins, Prosthetic (	Cofactors, Vitamins, Prosth	0	0	0	0	1	0	1	0
tRNA (5-methylaminomethyl-2-thiouridylate)-methyltransferase (EC 2.1.1.61)	Cofactors, Vitamins, Prosthetic (	Riboflavin, FMN, FAD	0	0	0	0	0	0	0	1
tRNA pseudouridine synthase B (EC 4.2.1.70)	Cofactors, Vitamins, Prosthetic (	Riboflavin, FMN, FAD	0	0	0	0	0	1	0	1
Ubiquinone/menaquinone biosynthesis methyltransferase UbiE (EC 2.1.1.-) @ 2-he	Cofactors, Vitamins, Prosthetic (	Quinone cofactors	0	0	0	0	0	0	0	1
Uroporphyrinogen-III synthase (EC 4.2.1.75)	Cofactors, Vitamins, Prosthetic (	Tetrapyrroles	0	0	0	0	0	0	0	1
Vitamin B12 ABC transporter, ATPase component BtuD	Cofactors, Vitamins, Prosthetic (	Tetrapyrroles	0	0	0	0	0	0	1	0
Vitamin B12 ABC transporter, B12-binding component BtuF	Cofactors, Vitamins, Prosthetic (	Tetrapyrroles	0	0	0	0	0	0	0	1
ATP-dependent helicase DinG/Rad3	DNA Metabolism	DNA repair	0	0	0	0	0	1	0	0
Deoxyribonuclease TatD	DNA Metabolism	DNA Metabolism - no subc	0	0	0	0	0	0	0	1
DNA polymerase II (EC 2.7.7.7)	DNA Metabolism	DNA repair	0	0	0	0	0	1	0	0
DNA polymerase III psi subunit (EC 2.7.7.7)	DNA Metabolism	DNA uptake, competence	0	0	0	0	0	0	0	1
DNA repair protein RadC	DNA Metabolism	DNA repair	0	0	0	1	0	0	0	0
DNA repair protein RecN	DNA Metabolism	DNA repair	0	0	0	0	0	0	1	0
DNA-3-methyladenine glycosylase II (EC 3.2.2.21)	DNA Metabolism	DNA repair	0	0	0	0	0	0	1	0
DNA-binding protein Fis	DNA Metabolism	DNA Metabolism - no subc	0	0	0	0	0	1	0	0
DNA-damage-inducible protein D	DNA Metabolism	DNA repair	0	0	0	0	1	0	0	0
DNA-damage-inducible protein F	DNA Metabolism	DNA repair	0	0	0	0	0	1	0	0
DNA-damage-inducible protein I	DNA Metabolism	DNA repair	0	0	0	0	0	0	1	0
Endonuclease I precursor (EC 3.1.21.1) @ Extracellular deoxyribonuclease Dns (EC	DNA Metabolism	DNA repair	0	0	0	0	0	1	0	1
Endonuclease III (EC 4.2.99.18)	DNA Metabolism	DNA repair	0	0	0	0	0	1	0	0
Exodeoxyribonuclease VII large subunit (EC 3.1.11.6)	DNA Metabolism	DNA repair	0	0	0	0	0	1	0	0
Exonuclease SbcD	DNA Metabolism	DNA repair	0	0	0	0	0	0	0	1
Protein involved in catabolism of external DNA	DNA Metabolism	DNA uptake, competence	0	0	0	0	0	0	0	1
Single-stranded-DNA-specific exonuclease RecJ (EC 3.1.-.-)	DNA Metabolism	DNA repair	0	0	0	0	0	0	0	1
Uracil-DNA glycosylase, family 1	DNA Metabolism	DNA repair	0	0	0	0	0	0	0	1
Cell division inhibitor - sulA	Dormancy and Sporulation	Dormancy and Sporulatior	0	0	0	0	0	0	1	0
Conidiation-specific protein 10	Dormancy and Sporulation	Dormancy and Sporulatior	0	0	0	0	0	0	1	0
FIG004684: SpoVR-like protein	Dormancy and Sporulation	Dormancy and Sporulatior	0	0	0	0	1	0	1	0
YaaH protein	Dormancy and Sporulation	Dormancy and Sporulatior	0	0	0	0	0	0	0	1
3-hydroxyacyl-[acyl-carrier-protein] dehydratase, FabA form (EC 4.2.1.59)	Fatty Acids, Lipids, and Isopreno	Fatty acids, biosynthesis	0	0	0	0	0	0	0	1
3-hydroxyacyl-[acyl-carrier-protein] dehydratase, FabZ form (EC 4.2.1.59)	Fatty Acids, Lipids, and Isopreno	Fatty acids, biosynthesis	0	0	0	0	0	0	0	1

Chromosomal Genes (Plasmid-borne genes listed separately below)	**SEED CATEGORY**	**SEED SUBCATEGORY**	BR7-2h-up	BR7-2h-down	BR21-2h-up	BR21-2h-down	BR7-8h-up	BR7-8h-down	BR21-8h-up	BR21-8h-down
3-hydroxybutyryl-CoA dehydrogenase (EC 1.1.1.157)	Fatty Acids, Lipids, and Isopreno	Fatty acids	0	0	0	0	1	0	1	0
3-oxoacyl-[acyl-carrier protein] reductase (EC 1.1.1.100)	Fatty Acids, Lipids, and Isopreno	Fatty acids, biosynthesis	0	0	0	0	0	0	0	1
Acyl carrier protein	Fatty Acids, Lipids, and Isopreno	Phospholipids	0	0	0	0	0	0	0	1
Acyl-CoA dehydrogenase (EC 1.3.8.7)	Fatty Acids, Lipids, and Isopreno	Fatty acids	0	0	0	0	1	0	1	0
Acyl-CoA dehydrogenase; probable dibenzothiophene desulfurization enzyme	Fatty Acids, Lipids, and Isopreno	Fatty acids	0	0	0	0	1	0	1	0
Butyryl-CoA dehydrogenase (EC 1.3.99.2)	Fatty Acids, Lipids, and Isopreno	Fatty acids	0	0	0	0	1	0	1	0
D-beta-hydroxybutyrate dehydrogenase (EC 1.1.1.30)	Fatty Acids, Lipids, and Isopreno	Fatty Acids, Lipids, and Isoр	0	0	0	0	0	0	1	0
D-beta-hydroxybutyrate permease	Fatty Acids, Lipids, and Isopreno	Fatty Acids, Lipids, and Isoр	0	0	0	0	1	0	0	0
Enoyl-[acyl-carrier-protein] reductase [NADH] (EC 1.3.1.9)	Fatty Acids, Lipids, and Isopreno	Fatty acids	0	0	0	0	0	0	0	1
Enoyl-CoA hydratase (EC 4.2.1.17) / Delta(3)-cis-delta(2)-trans-enoyl-CoA isomeras	Fatty Acids, Lipids, and Isopreno	Fatty acids	0	0	0	0	1	0	1	0
Fatty acid desaturase	Fatty Acids, Lipids, and Isopreno	Fatty acids	0	0	0	0	0	0	1	0
Malonyl CoA-acyl carrier protein transacylase (EC 2.3.1.39)	Fatty Acids, Lipids, and Isopreno	Fatty acids, biosynthesis	0	0	0	0	0	0	0	1
Medium-chain-fatty-acid--CoA ligase (EC 6.2.1.-)	Fatty Acids, Lipids, and Isopreno	Fatty acids	0	0	0	0	0	0	1	0
Phosphate:acyl-ACP acyltransferase PlsX	Fatty Acids, Lipids, and Isopreno	Phospholipids	0	0	0	0	0	0	0	1
Putative phosphatidylglycerophosphate synthase	Fatty Acids, Lipids, and Isopreno	Phospholipids	0	0	0	0	0	0	0	1
2,3-dihydro-2,3-dihydroxybenzoate dehydrogenase (EC 1.3.1.28) [enterobactin] si	Iron acquisition and metabolism	Siderophores	0	0	0	0	0	0	1	0
2,3-dihydroxybenzoate-AMP ligase (EC 2.7.7.58) of siderophore biosynthesis	Iron acquisition and metabolism	Siderophores	0	0	1	0	0	1	1	0
ABC transporter (iron.B12.siderophore.hemin) , ATP-binding component	Iron acquisition and metabolism	Iron acquisition and metab	0	0	0	0	0	0	1	0
ABC transporter (iron.B12.siderophore.hemin) , periplasmic substrate-binding com	Iron acquisition and metabolism	Iron acquisition and metab	1	0	0	0	0	1	1	0
ABC transporter (iron.B12.siderophore.hemin) , permease component	Iron acquisition and metabolism	Iron acquisition and metab	0	0	0	0	0	1	1	0
ABC-type Fe3+-siderophore transport system, ATPase component	Iron acquisition and metabolism	Siderophores	0	0	1	0	0	1	1	0
Aerobactin siderophore receptor IutA	Iron acquisition and metabolism	Siderophores	0	0	0	0	0	1	0	0
Bacterioferritin	Iron acquisition and metabolism	Iron acquisition and metab	0	0	0	0	1	0	1	0
Enterobactin esterase	Iron acquisition and metabolism	Siderophores	0	0	1	0	0	0	1	0
Enterobactin exporter EntS	Iron acquisition and metabolism	Siderophores	0	0	1	0	0	0	1	0
Enterobactin synthetase component F, serine activating enzyme (EC 2.7.7.-); entE	Iron acquisition and metabolism	Siderophores	0	0	1	0	0	1	1	0
Ferric enterobactin transport system permease protein FepD (TC 3.A.1.14.2)	Iron acquisition and metabolism	Siderophores	0	0	0	0	0	1	1	0
Ferric enterobactin transport system permease protein FepG (TC 3.A.1.14.2)	Iron acquisition and metabolism	Siderophores	0	0	1	0	0	1	1	0
Ferric enterobactin-binding periplasmic protein FepB (TC 3.A.1.14.2)	Iron acquisition and metabolism	Siderophores	0	0	0	0	0	0	1	0
Ferric hydroxamate ABC transporter (TC 3.A.1.14.3), ATP-binding protein FhuC	Iron acquisition and metabolism	Siderophores	0	0	1	0	0	1	1	0
Ferric hydroxamate ABC transporter (TC 3.A.1.14.3), periplasmic substrate binding	Iron acquisition and metabolism	Siderophores	0	0	1	0	0	1	0	0
Ferric hydroxamate ABC transporter (TC 3.A.1.14.3), permease component FhuB	Iron acquisition and metabolism	Siderophores	0	0	1	0	0	1	0	0
Ferric hydroxamate outer membrane receptor FhuA	Iron acquisition and metabolism	Siderophores	0	0	1	0	0	0	1	0
Ferric iron ABC transporter, iron-binding protein	Iron acquisition and metabolism	Iron acquisition and metab	0	0	0	0	0	0	1	0
Ferric iron ABC transporter, permease protein	Iron acquisition and metabolism	Iron acquisition and metab	1	0	0	0	1	0	1	0
Ferric reductase (1.6.99.14); fhuF	Iron acquisition and metabolism	Iron acquisition and metab	0	0	0	0	0	0	1	0
Ferric siderophore transport system, periplasmic binding protein TonB	Iron acquisition and metabolism	Iron acquisition and metab	0	0	0	0	0	1	0	0
Ferrichrome-iron receptor	Iron acquisition and metabolism	Iron acquisition and metab	0	0	1	0	0	1	1	0
Ferritin-like protein 2	Iron acquisition and metabolism	Iron acquisition and metab	0	0	0	0	0	0	0	1
Ferrous iron transport periplasmic protein EfeO, contains peptidase-M75 domain a	Iron acquisition and metabolism	Iron acquisition and metab	0	0	0	0	0	1	0	0
Ferrous iron transport permease EfeU	Iron acquisition and metabolism	Iron acquisition and metab	0	0	0	0	0	1	1	0
Ferrous iron transport peroxidase EfeB	Iron acquisition and metabolism	Iron acquisition and metab	0	0	0	0	0	1	0	0
Ferrous iron transport protein A	Iron acquisition and metabolism	Iron acquisition and metab	0	1	0	0	0	1	0	0
Ferrous iron transport protein B	Iron acquisition and metabolism	Iron acquisition and metab	0	1	0	0	0	1	0	0
Iron binding protein SufA for iron-sulfur cluster assembly	Iron acquisition and metabolism	Iron acquisition and metab	0	0	0	0	0	0	1	0
Isochorismatase (EC 3.3.2.1) [enterobactin] siderophore	Iron acquisition and metabolism	Siderophores	0	0	0	0	0	1	1	0
Periplasmic esterase IroE	Iron acquisition and metabolism	Iron acquisition and metab	0	0	0	0	0	0	1	0
Proofreading thioesterase in enterobactin biosynthesis EntH	Iron acquisition and metabolism	Siderophores	0	0	0	0	0	1	1	0
Putative ABC transporter, periplasmic iron binding protein precursor	Iron acquisition and metabolism	Siderophores	0	0	0	0	1	0	1	0
Putative iron compound permease protein of ABC transporter family	Iron acquisition and metabolism	Iron acquisition and metab	0	0	1	0	0	1	1	0

Chromosomal Genes (Plasmid-borne genes listed separately below)	SEED CATEGORY	SEED SUBCATEGORY	BR7-2h-up	BR7-2h-down	BR21-2h-up	BR21-2h-down	BR7-8h-up	BR7-8h-down	BR21-8h-up	BR21-8h-down
putative permease of ferrichrome ABC transporter	Iron acquisition and metabolism	Iron acquisition and metab	0	0	0	0	0	0	1	0
Sulfur acceptor protein SufE for iron-sulfur cluster assembly	Iron acquisition and metabolism	Iron acquisition and metab	0	0	0	0	0	0	1	0
TonB-dependent receptor; Outer membrane receptor for ferric enterobactin and c	Iron acquisition and metabolism	Iron acquisition and metab	0	0	1	0	0	1	1	0
Non-specific DNA-binding protein Dps / Iron-binding ferritin-like antioxidant protei	Iron acquisition and metabolism	Iron acquisition and metab	0	0	0	0	1	0	1	0
ABC sugar transporter, ATP-binding subunit	Membrane Transport	ABC transporters	0	0	0	0	0	0	1	0
ABC transporter ATP-binding protein	Membrane Transport	ABC transporters	0	0	0	0	0	0	1	0
ABC transporter, permease protein YnjC	Membrane Transport	ABC transporters	0	0	0	0	0	1	0	0
amino acid ABC transporter, permease protein	Membrane Transport	ABC transporters	0	0	0	0	0	1	0	0
Biopolymer transport protein ExbD/TolR	Membrane Transport	Membrane Transport - no	0	0	0	0	0	1	0	0
Branched-chain amino acid ABC transporter, amino acid-binding protein (TC 3.A.1.	Membrane Transport	ABC transporters	0	0	0	0	1	0	0	0
Calcium/proton antiporter	Membrane Transport	Cation transporters	0	0	0	0	0	0	1	0
Cation transport protein chaC	Membrane Transport	Cation transporters	0	0	0	0	0	0	1	0
Cation transport regulator chaB	Membrane Transport	Cation transporters	0	0	0	0	1	0	1	0
Chaperone protein fimC precursor	Membrane Transport	Protein secretion system, T	0	0	0	0	0	0	0	1
Colicin I receptor precursor	Membrane Transport	Membrane Transport - no	0	0	0	0	0	1	1	0
Di/tripeptide permease DtpA	Membrane Transport	Uni- Sym- and Antiporters	0	0	0	0	0	0	1	0
Di/tripeptide permease YbgH	Membrane Transport	Uni- Sym- and Antiporters	0	0	0	0	0	0	0	1
Di-/tripeptide transporter	Membrane Transport	Uni- Sym- and Antiporters	0	0	0	0	1	0	1	0
Dipeptide transport ATP-binding protein DppD (TC 3.A.1.5.2)	Membrane Transport	ABC transporters	0	0	0	0	0	0	1	0
Dipeptide transport ATP-binding protein DppF (TC 3.A.1.5.2)	Membrane Transport	ABC transporters	0	0	0	0	0	0	1	0
Dipeptide transport system permease protein DppB (TC 3.A.1.5.2)	Membrane Transport	ABC transporters	0	0	0	0	1	0	1	0
Dipeptide transport system permease protein DppC (TC 3.A.1.5.2)	Membrane Transport	ABC transporters	0	0	0	0	0	0	1	0
Dipeptide-binding ABC transporter, periplasmic substrate-binding component (TC 3	Membrane Transport	ABC transporters	0	0	0	0	0	0	1	0
FIGfam014588: Predicted regulator of CFA/I fimbriae	Membrane Transport	Protein secretion system, T	0	0	0	0	0	0	1	0
Fimbriae usher protein StcC	Membrane Transport	Protein secretion system, T	0	0	0	0	0	0	0	1
General secretion pathway protein D	Membrane Transport	Protein secretion system, T	0	0	0	0	1	0	0	1
General secretion pathway protein E	Membrane Transport	Protein secretion system, T	0	0	0	0	1	0	0	1
General secretion pathway protein F	Membrane Transport	Protein secretion system, T	0	0	0	0	0	0	0	1
General secretion pathway protein G	Membrane Transport	Protein secretion system, T	0	0	0	0	0	0	0	1
General secretion pathway protein J	Membrane Transport	Protein secretion system, T	0	0	0	0	1	0	0	0
General secretion pathway protein K	Membrane Transport	Protein secretion system, T	0	0	0	0	0	0	0	1
General secretion pathway protein L	Membrane Transport	Protein secretion system, T	0	0	0	0	1	0	0	1
General secretion pathway protein M	Membrane Transport	Protein secretion system, T	0	0	0	0	0	0	0	1
Glutamine ABC transporter, periplasmic glutamine-binding protein (TC 3.A.1.3.2)	Membrane Transport	ABC transporters	0	0	0	0	1	0	1	0
H(+)/Cl(-) exchange transporter ClcA	Membrane Transport	Cation transporters	0	0	0	0	0	1	0	0
Hypothetical MFS-type transporter protein YcaD	Membrane Transport	MFS transporter	0	0	0	0	0	0	1	0
Leader peptidase (Prepilin peptidase) (EC 3.4.23.43)	Membrane Transport	Protein and nucleoprotein	0	0	0	0	0	0	0	1
Magnesium and cobalt efflux protein CorC	Membrane Transport	Cation transporters	0	0	0	0	0	0	0	1
Manganese ABC transporter, ATP-binding protein SitB	Membrane Transport	ABC transporters	0	0	1	0	0	0	1	0
Manganese ABC transporter, inner membrane permease protein SitC	Membrane Transport	ABC transporters	0	0	0	0	0	0	1	0
Manganese ABC transporter, inner membrane permease protein SitD	Membrane Transport	ABC transporters	0	0	0	0	0	0	1	0
Manganese ABC transporter, periplasmic-binding protein SitA	Membrane Transport	ABC transporters	0	0	0	0	0	0	1	0
Manganese transport protein MntH	Membrane Transport	Membrane Transport - no	0	0	0	0	0	0	1	0
Mg(2+) transport ATPase protein C	Membrane Transport	Cation transporters	0	0	0	0	0	0	1	0
Mg/Co/Ni transporter MgtE / CBS domain	Membrane Transport	Membrane Transport - no	0	0	0	0	0	0	1	0
Na+/H+ antiporter NhaA type	Membrane Transport	Uni- Sym- and Antiporters	0	0	0	0	0	1	0	1
Nickel ABC transporter, periplasmic nickel-binding protein NikA (TC 3.A.1.5.3)	Membrane Transport	Cation transporters	0	1	0	0	0	1	0	1
Nickel transport ATP-binding protein NikD (TC 3.A.1.5.3)	Membrane Transport	Cation transporters	0	0	0	0	0	1	0	0
Nickel transport ATP-binding protein NikE (TC 3.A.1.5.3)	Membrane Transport	Cation transporters	0	0	0	0	0	0	0	1
Nickel transport system permease protein NikB (TC 3.A.1.5.3)	Membrane Transport	Cation transporters	0	0	0	0	0	0	0	1

Chromosomal Genes (Plasmid-borne genes listed separately below)	**SEED CATEGORY**	**SEED SUBCATEGORY**	BR7-2h-up	BR7-2h-down	BR21-2h-up	BR21-2h-down	BR7-8h-up	BR7-8h-down	BR21-8h-up	BR21-8h-down
Nickel transport system permease protein NikC (TC 3.A.1.5.3)	Membrane Transport	Cation transporters	0	0	0	0	0	0	0	1
Nucleoside permease NupC	Membrane Transport	Membrane Transport - no	0	0	0	0	0	0	0	1
Nucleoside permease NupG	Membrane Transport	Membrane Transport - no	0	0	0	0	0	0	1	0
Nucleoside-specific channel-forming protein Tsx precursor	Membrane Transport	nucleoside transport	0	0	0	0	0	0	0	1
Oligopeptide ABC transporter, periplasmic oligopeptide-binding protein OppA (TC 3	Membrane Transport	ABC transporters	0	0	0	0	1	0	1	0
Oligopeptide transport ATP-binding protein OppD (TC 3.A.1.5.1)	Membrane Transport	ABC transporters	0	0	0	0	1	0	0	0
Oligopeptide transport system permease protein OppB (TC 3.A.1.5.1)	Membrane Transport	ABC transporters	0	0	0	0	1	0	1	0
Oligopeptide transport system permease protein OppC (TC 3.A.1.5.1)	Membrane Transport	ABC transporters	0	0	0	0	0	0	1	0
Outer membrane protein C precursor	Membrane Transport	Cation transporters	0	0	0	0	0	0	0	1
Outer membrane protein X precursor	Membrane Transport	Cation transporters	0	0	0	0	0	0	0	1
PfkB family carbohydrate kinase Z5686, in cluster with transporter and aldolase	Membrane Transport	Membrane Transport - no	0	0	0	0	0	0	1	0
Phosphoglycerate transport system sensor protein PgtB (EC 2.7.3.-)	Membrane Transport	Membrane Transport - no	0	0	0	0	0	0	1	0
Phosphoglycerate transport system transcriptional regulatory protein PgtA	Membrane Transport	Membrane Transport - no	0	0	0	0	0	0	1	0
Phosphoglycerate transporter protein PgtP	Membrane Transport	Membrane Transport - no	1	0	0	0	0	0	1	0
Phosphonate ABC transporter ATP-binding protein (TC 3.A.1.9.1)	Membrane Transport	ABC transporters	0	0	0	0	0	0	1	0
Phosphonate ABC transporter phosphate-binding periplasmic component (TC 3.A.1	Membrane Transport	ABC transporters	0	0	0	0	0	0	1	0
Predicted membrane fusion protein (MFP) component of efflux pump, membrane	Membrane Transport	ABC transporters	0	0	0	0	0	0	1	0
Probable glucarate transporter	Membrane Transport	ABC transporters	0	0	0	0	1	0	1	0
Probable MFS transporter	Membrane Transport	MFS transporter	0	0	0	0	0	0	0	1
Putative ATPase component of ABC transporter with duplicated ATPase domain	Membrane Transport	ABC transporters	0	0	0	0	0	0	0	1
Putative Na(+)/H(+) exchanger protein, CPA1 family precursor	Membrane Transport	Cation transporters	0	0	0	0	0	0	0	1
Putative PerM family permease	Membrane Transport	Membrane Transport - no	0	0	0	0	0	0	1	0
Putative sugar ABC transport system, ATP-binding protein YtfR (EC 3.6.3.17)	Membrane Transport	ABC transporters	0	0	0	0	0	0	1	0
Putative sugar ABC transport system, periplasmic binding protein YtfQ precursor	Membrane Transport	ABC transporters	0	0	0	0	0	0	1	0
Putative sugar ABC transport system, permease protein YjfF	Membrane Transport	ABC transporters	0	0	0	0	0	0	1	0
Putative sugar ABC transport system, permease protein YtfT	Membrane Transport	ABC transporters	0	0	0	0	0	0	1	0
Putative sugar ABC transporter	Membrane Transport	ABC transporters	0	0	0	0	0	0	1	0
Shikimate transporter	Membrane Transport	MFS transporter	0	0	0	0	0	0	1	0
sodium-solute symporter, putative	Membrane Transport	Cation transporters	0	0	0	0	0	0	1	0
Sugar efflux transporter SotB	Membrane Transport	Membrane Transport - no	0	0	0	0	0	0	0	1
Sugar-binding protein precursor	Membrane Transport	Membrane Transport - no	0	0	0	0	0	0	1	0
sugar-proton symporter	Membrane Transport	Membrane Transport - no	0	0	0	0	0	0	1	0
Transcriptional activator NhaR	Membrane Transport	Uni- Sym- and Antiporters	0	0	0	0	0	0	0	1
Transporter, MFS superfamily	Membrane Transport	MFS transporter	0	0	0	0	0	0	1	0
Tryptophan-specific transport protein	Membrane Transport	ABC transporters	0	0	0	0	0	0	1	0
type 1 fimbriae major subunit FimA	Membrane Transport	Protein secretion system, T	0	0	0	0	1	0	0	0
Type-1 fimbrial protein, A chain precursor	Membrane Transport	Protein secretion system, T	0	0	0	0	0	1	0	1
Uncharacterized protein ImpC	Membrane Transport	Protein secretion system, T	0	0	0	0	0	0	1	0
Uncharacterized protein similar to VCA0109	Membrane Transport	Protein secretion system, T	0	0	0	0	0	0	1	0
2-keto-3-deoxy-D-arabino-heptulosonate-7- phosphate synthase I alpha (EC 2.5.1.5	Metabolism of Aromatic Compo	Peripheral pathways for ca	0	0	0	0	0	0	1	0
2-oxo-hepta-3-ene-1,7-dioic acid hydratase (EC 4.2.-.-)	Metabolism of Aromatic Compo	Metabolism of central arom	0	0	0	0	0	0	1	0
3-carboxy-cis,cis-muconate cycloisomerase (EC 5.5.1.2)	Metabolism of Aromatic Compo	Metabolism of central arom	0	0	0	0	0	0	1	0
3-hydroxyacyl-CoA dehydrogenase PaaC (EC 1.1.1.-)	Metabolism of Aromatic Compo	Metabolism of Aromatic C	0	0	0	0	0	0	1	0
3-oxoadipate CoA-transferase subunit A (EC 2.8.3.6)	Metabolism of Aromatic Compo	Peripheral pathways for ca	0	0	0	0	1	0	1	0
4-hydroxyphenylacetate 3-monooxygenase (EC 1.14.13.3)	Metabolism of Aromatic Compo	Metabolism of central arom	0	0	0	0	1	0	0	0
4-hydroxyphenylacetate symporter, major facilitator superfamily (MFS)	Metabolism of Aromatic Compo	Metabolism of central arom	0	0	0	0	1	0	1	0
Aldehyde dehydrogenase (EC 1.2.1.3), PaaZ	Metabolism of Aromatic Compo	Metabolism of Aromatic C	0	0	0	0	1	0	1	0
Beta-ketoadipate enol-lactone hydrolase (EC 3.1.1.24)	Metabolism of Aromatic Compo	Peripheral pathways for ca	0	0	0	0	0	0	1	0
Beta-ketoadipyl CoA thiolase (EC 2.3.1.-)	Metabolism of Aromatic Compo	Peripheral pathways for ca	0	0	0	0	1	0	1	0
Mhp operon transcriptional activator	Metabolism of Aromatic Compo	Peripheral pathways for ca	0	0	0	0	1	0	1	0

Chromosomal Genes (Plasmid-borne genes listed separately below)	SEED CATEGORY	SEED SUBCATEGORY	BR7-2h-up	BR7-2h-down	BR21-2h-up	BR21-2h-down	BR7-8h-up	BR7-8h-down	BR21-8h-up	BR21-8h-down
Muconate cycloisomerase (EC 5.5.1.1)	Metabolism of Aromatic Compo	Metabolism of central aro	0	0	0	0	1	0	1	0
Pca regulon regulatory protein PcaR	Metabolism of Aromatic Compo	Metabolism of central aro	0	0	0	0	0	0	1	0
Phenolic acid decarboxylase (EC 4.1.1.-)	Metabolism of Aromatic Compo	Metabolism of central aro	0	0	0	0	1	0	1	0
Phenylacetaldehyde dehydrogenase (EC 1.2.1.39)	Metabolism of Aromatic Compo	Metabolism of Aromatic C	0	0	0	0	1	0	1	0
Phenylacetic acid degradation operon negative regulatory protein PaaX	Metabolism of Aromatic Compo	Metabolism of Aromatic C	0	0	0	0	0	0	1	0
Phenylacetic acid degradation protein PaaY	Metabolism of Aromatic Compo	Metabolism of Aromatic C	0	0	0	0	0	0	1	0
P-hydroxybenzoate hydroxylase (EC 1.14.13.2)	Metabolism of Aromatic Compo	Peripheral pathways for ca	1	0	0	0	1	0	1	0
Protocatechuate 3,4-dioxygenase alpha chain (EC 1.13.11.3)	Metabolism of Aromatic Compo	Metabolism of central aro	0	0	0	0	0	0	1	0
Protocatechuate 3,4-dioxygenase beta chain (EC 1.13.11.3)	Metabolism of Aromatic Compo	Metabolism of central aro	0	0	0	0	1	0	1	0
Putative carboxymethylenebutenolidase (EC 3.1.1.45)	Metabolism of Aromatic Compo	Peripheral pathways for ca	0	0	0	0	1	0	1	0
Putative oxidoreductase YncB	Miscellaneous	Miscellaneous - no subcate	0	0	0	0	0	0	1	0
UPF0028 protein YchK aka rssA (rssB stab RpoS)	Miscellaneous	Miscellaneous - no subcate	0	0	0	0	0	0	1	0
UPF0265 protein YeeX	Miscellaneous	Miscellaneous - no subcate	0	0	0	0	0	0	0	1
YbbM seven transmembrane helix protein	Miscellaneous	Miscellaneous - no subcate	0	0	0	0	0	0	0	1
[Protein-PII] uridylyltransferase (EC 2.7.7.59)	Nitrogen Metabolism	Nitrogen Metabolism - no	0	0	0	0	0	0	0	1
Ammonium transporter	Nitrogen Metabolism	Nitrogen Metabolism - no	0	1	0	0	0	1	0	0
Hydroxylamine reductase (EC 1.7.-.-)	Nitrogen Metabolism	Nitrogen Metabolism - no	0	1	0	0	0	0	0	0
Nitrate/nitrite response regulator protein	Nitrogen Metabolism	Nitrogen Metabolism - no	0	1	0	0	0	1	0	1
Nitrate/nitrite sensor protein (EC 2.7.3.-)	Nitrogen Metabolism	Nitrogen Metabolism - no	0	1	0	0	0	1	0	1
Nitrate/nitrite transporter	Nitrogen Metabolism	Nitrogen Metabolism - no	0	1	0	0	0	1	0	1
Nitrate/nitrite transporter NarK	Nitrogen Metabolism	Denitrification	0	0	0	0	1	0	1	0
Nitrite reductase [NAD(P)H] large subunit (EC 1.7.1.4)	Nitrogen Metabolism	Nitrogen Metabolism - no	0	1	0	0	0	1	0	1
Nitrite reductase [NAD(P)H] small subunit (EC 1.7.1.4)	Nitrogen Metabolism	Nitrogen Metabolism - no	0	1	0	0	0	1	0	1
Nitrogen regulatory protein P-II, glnK	Nitrogen Metabolism	Nitrogen Metabolism - no	0	0	0	0	0	1	0	0
Respiratory nitrate reductase alpha chain, narG (EC 1.7.99.4)	Nitrogen Metabolism	Nitrogen Metabolism - no	0	1	0	0	0	1	0	1
Respiratory nitrate reductase beta chain (EC 1.7.99.4)	Nitrogen Metabolism	Nitrogen Metabolism - no	0	1	0	1	0	1	0	1
Respiratory nitrate reductase delta chain (EC 1.7.99.4)	Nitrogen Metabolism	Nitrogen Metabolism - no	0	0	0	1	0	1	0	1
Respiratory nitrate reductase gamma chain (EC 1.7.99.4)	Nitrogen Metabolism	Nitrogen Metabolism - no	0	0	0	1	0	0	0	1
2',3'-cyclic-nucleotide 2'-phosphodiesterase (EC 3.1.4.16)	Nucleosides and Nucleotides	Pyrimidines-Purines	0	0	0	0	0	0	0	1
5'-nucleotidase YjjG (EC 3.1.3.5)	Nucleosides and Nucleotides	Detoxification	0	0	0	0	0	0	0	1
Adenine deaminase (EC 3.5.4.2)	Nucleosides and Nucleotides	Purines	0	0	0	0	0	0	1	0
Cytidine deaminase (EC 3.5.4.5)	Nucleosides and Nucleotides	Pyrimidines	0	1	0	1	0	1	0	1
Cytosine/purine/uracil/thiamine/allantoin permease family protein	Nucleosides and Nucleotides	Purines	0	0	0	0	1	0	1	0
Deacetylases, including yeast histone deacetylase and acetoin utilization protein	Nucleosides and Nucleotides	Nucleosides and Nucleotid	0	0	1	0	1	0	1	0
Guanine-hypoxanthine permease	Nucleosides and Nucleotides	Purines	0	0	1	0	0	0	0	0
Guanylate kinase (EC 2.7.4.8)	Nucleosides and Nucleotides	Purines	0	0	0	0	0	0	0	1
Hydroxymethylpyrimidine phosphate synthase ThiC (EC 4.1.99.17)	Nucleosides and Nucleotides	Purines	0	0	0	0	1	0	1	0
Ribonucleotide reductase of class Ia (aerobic), alpha subunit (EC 1.17.4.1)	Nucleosides and Nucleotides	Nucleosides and Nucleotid	0	0	1	0	0	0	0	0
Ribonucleotide reductase of class III (anaerobic), activating protein (EC 1.97.1.4)	Nucleosides and Nucleotides	Nucleosides and Nucleotid	0	0	1	0	0	0	0	0
Xanthine permease	Nucleosides and Nucleotides	Purines	1	0	0	0	0	0	0	0
11 CDS (phage-related)	Phages, Prophages, Transposabl	Phages, Prophages	0	0	1	0	0	0	1	0
12 CDS (phage-related)	Phages, Prophages, Transposabl	Phages, Prophages	0	0	1	0	0	0	1	0
13 CDS (phage-related, hypothetical)	Phages, Prophages, Transposabl	Phages, Prophages	0	0	1	0	0	0	1	0
14 CDS (phage-related, hypothetical)	Phages, Prophages, Transposabl	Phages, Prophages	0	0	1	0	0	0	1	0
16 CDS (phage-related)	Phages, Prophages, Transposabl	Phages, Prophages	0	0	1	0	0	0	1	0
Adenine DNA methyltransferase, phage-associated	Phages, Prophages, Transposabl	Phages, Prophages	0	0	1	0	0	0	1	0
Baseplate assembly protein J	Phages, Prophages, Transposabl	Phages, Prophages	0	0	0	0	0	0	1	0
Baseplate assembly protein V	Phages, Prophages, Transposabl	Phages, Prophages	0	0	0	0	0	0	1	0
COG4834: Uncharacterized protein conserved in bacteria (putative bacteriophage)	Phages, Prophages, Transposabl	Phages, Prophages	1	0	1	0	0	0	1	0
corresponds to STY3665 from Accession AL513382: Salmonella typhi CT18	Phages, Prophages, Transposabl	Phages, Prophages	0	0	0	0	0	0	1	0

Chromosomal Genes (Plasmid-borne genes listed separately below)	**SEED CATEGORY**	**SEED SUBCATEGORY**	BR7-2h-up	BR7-2h-down	BR21-2h-up	BR21-2h-down	BR7-8h-up	BR7-8h-down	BR21-8h-up	BR21-8h-down
corresponds to STY4603 from Accession AL513382: Salmonella typhi CT18	Phages, Prophages, Transposabl	Phages, Prophages	0	0	0	0	0	0	1	0
Cro	Phages, Prophages, Transposabl	Phages, Prophages	0	0	1	0	0	0	1	0
DNA polymerase III alpha subunit (EC 2.7.7.7)	Phages, Prophages, Transposabl	Phages, Prophages	0	0	0	0	0	0	0	1
FIG00626372: hypothetical protein (cell division blocking protein, phage)	Phages, Prophages, Transposabl	Phages, Prophages	1	0	0	0	0	0	1	0
FIG00639045: hypothetical protein	Phages, Prophages, Transposabl	Phages, Prophages	0	0	1	0	0	0	1	0
FIG00639626: hypothetical protein	Phages, Prophages, Transposabl	Phages, Prophages	0	0	1	0	0	0	1	0
FIG00639781: hypothetical protein	Phages, Prophages, Transposabl	Phages, Prophages	0	0	1	0	0	0	1	0
FIG00640468: hypothetical protein	Phages, Prophages, Transposabl	Phages, Prophages	0	0	1	0	0	0	1	0
FIG00640622: hypothetical protein	Phages, Prophages, Transposabl	Phages, Prophages	0	0	1	0	0	0	1	0
FIG00640949: hypothetical protein	Phages, Prophages, Transposabl	Phages, Prophages	0	0	1	0	0	0	1	0
FIG00641226: hypothetical protein (SOS induction modulator, phage)	Phages, Prophages, Transposabl	Phages, Prophages	0	0	0	0	0	0	1	0
FIG00641614: hypothetical protein	Phages, Prophages, Transposabl	Phages, Prophages	0	0	1	0	0	0	1	0
FIG00641850: hypothetical protein	Phages, Prophages, Transposabl	Phages, Prophages	0	0	1	0	0	0	1	0
FIG00643583: hypothetical protein	Phages, Prophages, Transposabl	Phages, Prophages	0	0	1	0	0	0	1	0
FIG00949334: hypothetical protein	Phages, Prophages, Transposabl	Phages, Prophages	0	0	1	0	0	0	1	0
FIG01046174: hypothetical protein	Phages, Prophages, Transposabl	Phages, Prophages	0	0	1	0	0	0	1	0
FIG01070919: hypothetical protein	Phages, Prophages, Transposabl	Phages, Prophages	0	0	1	0	0	0	1	0
FIG01078329: hypothetical protein (phage recombination Bet)	Phages, Prophages, Transposabl	Phages, Prophages	1	0	1	0	0	0	1	0
FIG01221322: hypothetical protein	Phages, Prophages, Transposabl	Phages, Prophages	0	0	1	0	0	0	1	0
FIG070121: Phage capsid and scaffold protein	Phages, Prophages, Transposabl	Phages, Prophages	0	0	0	0	0	0	1	0
IS, phage, Tn; Transposon-related functions	Phages, Prophages, Transposabl	Phages, Prophages	1	0	1	0	0	0	1	0
Ner-like regulatory protein	Phages, Prophages, Transposabl	Phages, Prophages	0	0	0	0	0	0	0	1
Origin specific replication initiation factor	Phages, Prophages, Transposabl	Phages, Prophages	0	0	1	0	0	0	1	0
Phage capsid and scaffold	Phages, Prophages, Transposabl	Phages, Prophages	0	0	1	0	0	0	1	0
Phage capsid scaffolding protein	Phages, Prophages, Transposabl	Phages, Prophages	1	0	1	0	0	0	1	0
Phage endopeptidase (EC 3.4.-.-) Rz	Phages, Prophages, Transposabl	Phages, Prophages	0	0	1	0	0	0	1	0
Phage exonuclease (EC 3.1.11.3); Putative phage-encoded enzyme involved in inte	Phages, Prophages, Transposabl	Phages, Prophages	1	0	1	0	0	0	1	0
Phage head completion-stabilization protein	Phages, Prophages, Transposabl	Phages, Prophages	1	0	1	0	0	0	1	0
Phage major capsid protein	Phages, Prophages, Transposabl	Phages, Prophages	0	0	1	0	0	0	1	0
Phage major tail tube protein	Phages, Prophages, Transposabl	Phages, Prophages	0	0	1	0	0	0	1	0
Phage protein	Phages, Prophages, Transposabl	Phages, Prophages	0	0	1	0	0	0	1	0
Phage regulatory protein	Phages, Prophages, Transposabl	Phages, Prophages	0	0	0	0	0	0	1	0
Phage replication protein	Phages, Prophages, Transposabl	Phages, Prophages	0	0	0	0	0	0	1	0
Phage spanin Rz	Phages, Prophages, Transposabl	Phages, Prophages	0	0	0	0	0	0	1	0
Phage tail protein	Phages, Prophages, Transposabl	Phages, Prophages	0	0	0	0	0	0	1	0
Phage tail sheath monomer	Phages, Prophages, Transposabl	Phages, Prophages	0	0	0	0	0	0	1	0
Phage terminase small subunit	Phages, Prophages, Transposabl	Phages, Prophages	0	0	1	0	0	0	1	0
Phage terminase, ATPase subunit	Phages, Prophages, Transposabl	Phages, Prophages	0	0	0	0	0	0	1	0
Phage terminase, endonuclease subunit	Phages, Prophages, Transposabl	Phages, Prophages	0	0	1	0	0	0	1	0
Phage terminase, large subunit	Phages, Prophages, Transposabl	Phages, Prophages	0	0	1	0	0	0	1	0
Phage-related capsid packaging protein	Phages, Prophages, Transposabl	Phages, Prophages	0	0	0	0	0	0	1	0
Putative bacteriophage protein	Phages, Prophages, Transposabl	Phages, Prophages	0	0	1	0	0	0	1	0
putative tail protein	Phages, Prophages, Transposabl	Phages, Prophages	0	0	1	0	0	0	1	0
Regulatory protein CII	Phages, Prophages, Transposabl	Phages, Prophages	0	0	0	0	0	0	1	0
Ren protein	Phages, Prophages, Transposabl	Phages, Prophages	0	0	0	0	0	0	1	0
Tail fiber protein	Phages, Prophages, Transposabl	Phages, Prophages	0	0	1	0	0	0	1	0
Tail protein	Phages, Prophages, Transposabl	Phages, Prophages	0	0	0	0	0	0	1	0
Tail protein I	Phages, Prophages, Transposabl	Phages, Prophages	0	0	0	0	0	0	1	0
terminase B protein (see terminase B protein, putative for duplicate)	Phages, Prophages, Transposabl	Phages, Prophages	0	0	1	0	0	0	0	0
terminase B protein, putative	Phages, Prophages, Transposabl	Phages, Prophages	0	0	1	0	0	0	1	0

Chromosomal Genes (Plasmid-borne genes listed separately below)	SEED CATEGORY	SEED SUBCATEGORY	BR7-2h-up	BR7-2h-down	BR21-2h-up	BR21-2h-down	BR7-8h-up	BR7-8h-down	BR21-8h-up	BR21-8h-down
acid phosphatase	Phosphorus Metabolism	Phosphorus Metabolism - r	0	0	0	0	0	0	1	0
Alkaline phosphatase (EC 3.1.3.1); PhoA	Phosphorus Metabolism	Phosphorus Metabolism - r	0	0	0	0	1	0	1	0
Alkaline phosphatase isozyme conversion protein precursor (EC 3.4.11.-)	Phosphorus Metabolism	Phosphorus Metabolism - r	0	0	0	0	0	1	0	0
Alkylphosphonate utilization operon protein PhnA	Phosphorus Metabolism	Phosphorus Metabolism - r	0	0	0	0	0	0	0	1
Endonuclease/Exonuclease/phosphatase family protein	Phosphorus Metabolism	Phosphorus Metabolism - r	0	0	0	0	0	0	1	0
Inorganic pyrophosphatase (EC 3.6.1.1)	Phosphorus Metabolism	Phosphorus Metabolism - r	0	0	0	0	0	0	0	1
Inositol-1-monophosphatase (EC 3.1.3.25)	Phosphorus Metabolism	Phosphorus Metabolism - r	0	0	0	0	0	1	0	0
Phosphate regulon sensor protein PhoR (SphS) (EC 2.7.13.3)	Phosphorus Metabolism	Phosphorus Metabolism - r	0	0	0	0	0	0	1	0
Phosphate regulon transcriptional regulatory protein PhoB (SphR)	Phosphorus Metabolism	Phosphorus Metabolism - r	0	0	0	0	1	0	1	0
Promiscuous sugar phosphatase YidA, haloacid dehalogenase-like phosphatase fan	Phosphorus Metabolism	Phosphorus Metabolism - r	0	0	0	0	0	1	0	1
Soluble pyridine nucleotide transhydrogenase (EC 1.6.1.1)	Phosphorus Metabolism	Phosphorus Metabolism - r	1	0	0	0	1	0	1	0
FKBP-type peptidyl-prolyl cis-trans isomerase FkpA precursor (EC 5.2.1.8)	Potassium metabolism	Potassium metabolism - nc	0	0	0	0	0	0	0	1
FKBP-type peptidyl-prolyl cis-trans isomerase SlyD (EC 5.2.1.8)	Potassium metabolism	Potassium metabolism - nc	0	0	0	0	0	0	0	1
Osmosensitive K+ channel histidine kinase KdpD (EC 2.7.3.-)	Potassium metabolism	Potassium metabolism - nc	0	0	0	0	0	1	0	1
Potassium uptake protein TrkH	Potassium metabolism	Potassium metabolism - nc	0	0	0	0	0	0	0	1
Potassium-transporting ATPase A chain (EC 3.6.3.12) (TC 3.A.3.7.1)	Potassium metabolism	Potassium metabolism - nc	0	0	0	0	0	1	0	1
Potassium-transporting ATPase B chain (EC 3.6.3.12) (TC 3.A.3.7.1)	Potassium metabolism	Potassium metabolism - nc	0	0	0	0	0	0	0	1
Potassium-transporting ATPase C chain (EC 3.6.3.12) (TC 3.A.3.7.1)	Potassium metabolism	Potassium metabolism - nc	0	0	0	0	0	0	0	1
16S rRNA processing protein RimM	Protein Metabolism	Protein biosynthesis	0	0	0	0	0	0	0	1
6-carboxytetrahydropterin synthase (EC 4.1.2.50) @ Queuosine biosynthesis QueD	Protein Metabolism	Protein biosynthesis	0	0	0	0	0	1	0	0
Acidic protein msyB	Protein Metabolism	Protein folding	0	0	0	0	1	0	1	0
ATP-dependent Clp protease adaptor protein ClpS	Protein Metabolism	Protein degradation	0	0	0	0	0	0	0	1
ATP-dependent Clp protease ATP-binding subunit ClpA	Protein Metabolism	Protein degradation	0	0	0	0	1	0	0	0
Chaperone protein DnaJ	Protein Metabolism	Protein folding	0	0	0	0	0	0	0	1
Chaperone protein DnaK	Protein Metabolism	Protein folding	0	0	0	0	0	0	0	1
Chaperone protein HtpG	Protein Metabolism	Protein folding	0	0	0	0	0	0	0	1
Chaperone-modulator protein CbpM	Protein Metabolism	Protein folding	0	0	0	0	0	0	1	0
Cys-tRNA(Pro) deacylase YbaK	Protein Metabolism	Protein biosynthesis	0	0	0	0	0	0	0	1
Cytochrome c-type biogenesis protein DsbD, protein-disulfide reductase (EC 1.8.1.	Protein Metabolism	Protein folding	0	0	0	0	0	0	0	1
Epoxyqueuosine (oQ) reductase QueG	Protein Metabolism	Protein biosynthesis	0	0	0	0	0	1	0	1
FIG00731416: hypothetical protein	Protein Metabolism	Selenoproteins	0	1	0	0	0	1	0	1
FKBP-type peptidyl-prolyl cis-trans isomerase FklB (EC 5.2.1.8)	Protein Metabolism	Protein folding	0	0	0	0	0	0	0	1
Glutaminyl-tRNA synthetase (EC 6.1.1.18)	Protein Metabolism	Protein biosynthesis	0	0	0	0	0	0	0	1
Glycyl-tRNA synthetase beta chain (EC 6.1.1.14)	Protein Metabolism	Protein biosynthesis	0	0	0	0	0	0	0	1
GTP-binding protein EngB	Protein Metabolism	Protein biosynthesis	0	0	0	0	0	0	0	1
GTP-binding protein Obg	Protein Metabolism	Protein biosynthesis	0	0	0	0	0	1	0	0
GTP-binding protein TypA/BipA	Protein Metabolism	Protein biosynthesis	0	0	0	0	0	0	0	1
Heat shock protein 60 family co-chaperone GroES	Protein Metabolism	Protein folding	0	0	0	0	0	0	0	1
Lipoprotein signal peptidase (EC 3.4.23.36)	Protein Metabolism	Protein processing and mo	0	0	0	0	0	0	0	1
LSU ribosomal protein L10p (P0)	Protein Metabolism	Protein biosynthesis	0	0	0	0	0	0	0	1
LSU ribosomal protein L11p (L12e)	Protein Metabolism	Protein biosynthesis	0	0	0	0	0	0	0	1
LSU ribosomal protein L13p (L13Ae)	Protein Metabolism	Protein biosynthesis	0	0	0	0	0	0	0	1
LSU ribosomal protein L14p (L23e)	Protein Metabolism	Protein biosynthesis	0	0	0	0	0	0	0	1
LSU ribosomal protein L15p (L27Ae)	Protein Metabolism	Protein biosynthesis	0	0	0	0	0	1	0	1
LSU ribosomal protein L16p (L10e)	Protein Metabolism	Protein biosynthesis	0	0	0	0	0	0	0	1
LSU ribosomal protein L17p	Protein Metabolism	Protein biosynthesis	0	0	0	0	0	0	0	1
LSU ribosomal protein L18p (L5e)	Protein Metabolism	Protein biosynthesis	0	0	0	0	0	1	0	1
LSU ribosomal protein L19p	Protein Metabolism	Protein biosynthesis	0	0	0	0	0	0	0	1
LSU ribosomal protein L1p (L10Ae)	Protein Metabolism	Protein biosynthesis	0	0	0	0	0	0	0	1
LSU ribosomal protein L20p	Protein Metabolism	Protein biosynthesis	0	0	0	1	0	0	0	1

Chromosomal Genes (Plasmid-borne genes listed separately below)	**SEED CATEGORY**	**SEED SUBCATEGORY**	BR7-2h-up	BR7-2h-down	BR21-2h-up	BR21-2h-down	BR7-8h-up	BR7-8h-down	BR21-8h-up	BR21-8h-down
LSU ribosomal protein L22p (L17e)	Protein Metabolism	Protein biosynthesis	0	0	0	0	0	1	0	1
LSU ribosomal protein L23p (L23Ae)	Protein Metabolism	Protein biosynthesis	0	0	0	0	0	0	0	1
LSU ribosomal protein L24p (L26e)	Protein Metabolism	Protein biosynthesis	0	0	0	0	0	1	0	1
LSU ribosomal protein L28p	Protein Metabolism	Protein biosynthesis	0	0	0	0	0	0	0	1
LSU ribosomal protein L2p (L8e)	Protein Metabolism	Protein biosynthesis	0	0	0	0	0	0	0	1
LSU ribosomal protein L30p (L7e)	Protein Metabolism	Protein biosynthesis	0	0	0	0	0	0	0	1
LSU ribosomal protein L31p @ LSU ribosomal protein L31p, zinc-dependent	Protein Metabolism	Protein biosynthesis	0	0	0	0	0	0	0	1
LSU ribosomal protein L33p	Protein Metabolism	Protein biosynthesis	0	0	0	0	0	1	0	0
LSU ribosomal protein L33p @ LSU ribosomal protein L33p, zinc-independent	Protein Metabolism	Protein biosynthesis	0	0	0	0	0	0	0	1
LSU ribosomal protein L34p	Protein Metabolism	Protein biosynthesis	0	0	0	0	0	0	0	1
LSU ribosomal protein L3p (L3e)	Protein Metabolism	Protein biosynthesis	0	0	0	0	0	0	0	1
LSU ribosomal protein L4p (L1e)	Protein Metabolism	Protein biosynthesis	0	0	0	0	0	0	0	1
LSU ribosomal protein L5p (L11e)	Protein Metabolism	Protein biosynthesis	0	0	0	0	0	0	0	1
LSU ribosomal protein L6p (L9e)	Protein Metabolism	Protein biosynthesis	0	0	0	0	0	1	0	1
LSU ribosomal protein L7/L12 (P1/P2)	Protein Metabolism	Protein biosynthesis	0	0	0	0	0	1	0	1
Outer membrane usher protein	Protein Metabolism	Protein folding	0	0	0	0	0	0	1	0
Peptide chain release factor 3	Protein Metabolism	Protein biosynthesis	0	0	0	0	0	0	0	1
Phenylalanyl-tRNA synthetase alpha chain (EC 6.1.1.20)	Protein Metabolism	Protein biosynthesis	0	0	0	0	0	0	0	1
Phenylalanyl-tRNA synthetase beta chain (EC 6.1.1.20)	Protein Metabolism	Protein biosynthesis	0	0	0	0	0	0	0	1
Protease II (EC 3.4.21.83)	Protein Metabolism	Protein degradation	0	0	0	0	0	0	1	0
Putative SPFH domain protein (serine protease, homology to *hflc*)	Protein Metabolism	Protein degradation	1	0	1	0	0	0	1	0
Ribosomal large subunit pseudouridine synthase A (EC 4.2.1.70)	Protein Metabolism	Protein biosynthesis	0	0	0	0	0	0	0	1
Ribosome-binding factor A	Protein Metabolism	Protein biosynthesis	0	0	0	0	0	0	0	1
Ribulosamine/erythrulosamine 3-kinase potentially involved in protein deglycation	Protein Metabolism	Protein processing and mo	0	0	0	0	0	0	1	0
SSU ribosomal protein S10p (S20e)	Protein Metabolism	Protein biosynthesis	0	0	0	0	0	0	0	1
SSU ribosomal protein S14p (S29e)	Protein Metabolism	Protein biosynthesis	0	0	0	0	0	1	0	1
SSU ribosomal protein S15p (S13e)	Protein Metabolism	Protein biosynthesis	0	0	0	0	0	1	0	1
SSU ribosomal protein S16p	Protein Metabolism	Protein biosynthesis	0	0	0	0	0	1	0	0
SSU ribosomal protein S17p (S11e)	Protein Metabolism	Protein biosynthesis	0	0	0	0	0	1	0	1
SSU ribosomal protein S19p (S15e)	Protein Metabolism	Protein biosynthesis	0	0	0	0	0	0	0	1
SSU ribosomal protein S20p	Protein Metabolism	Protein biosynthesis	0	0	0	0	0	0	0	1
SSU ribosomal protein S2p (SAe)	Protein Metabolism	Protein biosynthesis	0	0	0	0	0	0	0	1
SSU ribosomal protein S3p (S3e)	Protein Metabolism	Protein biosynthesis	0	0	0	0	0	0	0	1
SSU ribosomal protein S5p (S2e)	Protein Metabolism	Protein biosynthesis	0	0	0	0	0	0	0	1
SSU ribosomal protein S6p	Protein Metabolism	Protein biosynthesis	0	0	0	0	0	0	0	1
SSU ribosomal protein S7p (S5e)	Protein Metabolism	Protein biosynthesis	0	0	0	0	0	0	0	1
SSU ribosomal protein S8p (S15Ae)	Protein Metabolism	Protein biosynthesis	0	0	0	0	0	0	0	1
SSU ribosomal protein S9p (S16e)	Protein Metabolism	Protein biosynthesis	0	0	0	0	0	0	0	1
Translation elongation factor G	Protein Metabolism	Protein biosynthesis	0	0	0	0	0	0	0	1
Translation elongation factor P	Protein Metabolism	Protein biosynthesis	0	0	0	0	0	0	0	1
Translation elongation factor Ts	Protein Metabolism	Protein biosynthesis	0	0	0	0	0	0	0	1
Translation elongation factor Tu	Protein Metabolism	Protein biosynthesis	0	0	0	0	0	0	0	1
Translation initiation factor 1	Protein Metabolism	Protein biosynthesis	0	0	0	0	0	1	0	1
Translation initiation factor 2	Protein Metabolism	Protein biosynthesis	0	0	0	0	0	0	0	1
Tripeptide aminopeptidase (EC 3.4.11.4)	Protein Metabolism	Protein degradation	0	0	0	0	0	0	0	1
tRNA (Guanine37-N1) -methyltransferase (EC 2.1.1.31)	Protein Metabolism	Protein biosynthesis	0	0	0	0	0	0	0	1
Antiholin-like protein LrgA	Regulation and Cell signaling	Programmed Cell Death ar	0	1	0	1	0	1	0	1
Autoinducer 2 (AI-2) ABC transport system, fused AI2 transporter subunits and ATP	Regulation and Cell signaling	Quorum sensing and biofil	0	0	0	0	1	0	1	0
Autoinducer 2 (AI-2) ABC transport system, membrane channel protein LsrC	Regulation and Cell signaling	Quorum sensing and biofil	0	0	0	0	1	0	1	0
Autoinducer 2 (AI-2) ABC transport system, membrane channel protein LsrD	Regulation and Cell signaling	Quorum sensing and biofil	0	0	0	0	1	0	1	0

Chromosomal Genes (Plasmid-borne genes listed separately below)	**SEED CATEGORY**	**SEED SUBCATEGORY**	BR7-2h-up	BR7-2h-down	BR21-2h-up	BR21-2h-down	BR7-8h-up	BR7-8h-down	BR21-8h-up	BR21-8h-down
Autoinducer 2 (AI-2) ABC transport system, periplasmic AI-2 binding protein LsrB	Regulation and Cell signaling	Quorum sensing and biofil	0	0	0	0	1	0	1	0
Autoinducer 2 (AI-2) aldolase LsrF (EC 4.2.1.-)	Regulation and Cell signaling	Quorum sensing and biofil	0	0	0	0	1	0	1	0
Autoinducer 2 (AI-2) kinase LsrK (EC 2.7.1.-)	Regulation and Cell signaling	Quorum sensing and biofil	0	0	0	0	1	0	1	0
Autoinducer 2 (AI-2) modifying protein LsrG	Regulation and Cell signaling	Quorum sensing and biofil	0	0	0	0	0	0	1	0
BAX protein	Regulation and Cell signaling	Programmed Cell Death ar	0	0	0	0	0	0	1	0
Biofilm PGA outer membrane secretin PgaA	Regulation and Cell signaling	Quorum sensing and biofil	0	0	0	0	0	0	1	0
biofilm regulator BssR	Regulation and Cell signaling	Quorum sensing and biofil	0	0	0	0	0	0	1	0
CidA-associated membrane protein CidB	Regulation and Cell signaling	Programmed Cell Death ar	0	1	0	1	0	1	0	1
Death on curing protein, Doc toxin	Regulation and Cell signaling	Programmed Cell Death ar	1	0	0	0	0	0	0	0
DNA transformation protein TfoX	Regulation and Cell signaling	Regulation and Cell signalir	0	0	0	0	1	0	1	0
DNA-binding capsular synthesis response regulator RcsB	Regulation and Cell signaling	Regulation and Cell signalir	0	0	0	0	0	0	1	0
Holin-like protein CidA	Regulation and Cell signaling	Programmed Cell Death ar	0	1	0	1	0	1	0	1
Hybrid sensory histidine kinase in two-component regulatory system with EvgA	Regulation and Cell signaling	Regulation and Cell signalir	0	0	0	0	0	1	0	1
LrgA-associated membrane protein LrgB	Regulation and Cell signaling	Programmed Cell Death ar	0	1	0	1	0	1	0	1
LsrR, transcriptional repressor of lsr operon	Regulation and Cell signaling	Quorum sensing and biofil	0	0	0	0	1	0	1	0
LysR family transcriptional regulator near succinyl-CoA:3-ketoacid-coenzyme A tra	Regulation and Cell signaling	Regulation and Cell signalir	1	0	0	0	1	0	1	0
LysR family transcriptional regulator STM3121	Regulation and Cell signaling	Regulation and Cell signalir	0	0	0	0	0	0	0	1
LysR family transcriptional regulator YdcI	Regulation and Cell signaling	Regulation and Cell signalir	1	0	0	0	1	0	1	0
LysR family transcriptional regulator YfiE	Regulation and Cell signaling	Regulation and Cell signalir	0	0	0	0	0	0	0	1
Phosphohistidine phosphatase SixA	Regulation and Cell signaling	Regulation and Cell signalir	0	0	0	0	0	0	1	0
Positive transcription regulator EvgA	Regulation and Cell signaling	Regulation and Cell signalir	0	0	0	0	0	1	0	0
putative sensory transduction regulator	Regulation and Cell signaling	Regulation and Cell signalir	0	0	0	0	0	1	0	0
putative two-component response regulator transcriptional regulatory protein	Regulation and Cell signaling	Regulation and Cell signalir	0	0	0	0	0	0	1	0
sensor histidine kinase	Regulation and Cell signaling	Regulation and Cell signalir	0	0	0	0	0	1	0	0
Sensory histidine kinase in two-component regulatory system with RstA	Regulation and Cell signaling	Regulation and Cell signalir	0	0	0	0	0	0	0	1
Threonine catabolic operon transcriptional activator TdcA	Regulation and Cell signaling	Regulation and Cell signalir	0	0	0	0	0	0	1	0
Transcriptional activator protein LysR	Regulation and Cell signaling	Regulation and Cell signalir	0	0	0	0	1	0	1	0
Type IV secretory pathway, VirB4 components	Regulation and Cell signaling	Regulation and Cell signalir	0	0	0	0	0	0	1	0
Zinc uptake regulation protein ZUR	Regulation and Cell signaling	Regulation and Cell signalir	0	0	0	0	0	0	0	1
[NiFe] hydrogenase metallocenter assembly protein HypD	Respiration	Electron donating reaction	0	1	0	0	0	0	0	0
Aerobic C4-dicarboxylate transporter for fumarate, L-malate, D-malate, succunate	Respiration	Electron accepting reactior	0	0	0	0	1	0	1	0
ATP synthase protein I	Respiration	Respiration - no subcatego	0	0	0	1	0	0	0	0
ATPase domain protein	Respiration	Respiration - no subcatego	0	0	0	0	0	0	0	1
Benzoate 1,2-dioxygenase (EC 1.14.12.10)	Respiration	Electron donating reaction	0	0	0	0	1	0	1	0
benzyl alcohol dehydrogenase	Respiration	Electron donating reaction	0	0	0	0	1	0	1	0
C4-dicarboxylate transport protein	Respiration	Electron accepting reactior	0	0	0	0	0	1	0	1
C4-dicarboxylate transporter DcuB	Respiration	Electron accepting reactior	1	0	0	0	0	0	0	0
Cytochrome b(561)	Respiration	Respiration - no subcatego	0	0	0	0	0	0	1	0
Electron transport complex protein RnfA	Respiration	Electron donating reaction	0	0	0	0	0	1	0	0
Electron transport protein HydN	Respiration	Electron donating reaction	0	0	0	0	0	0	0	1
Ferredoxin reductase	Respiration	Electron accepting reactior	0	0	0	0	1	0	1	0
FMN oxidoreductase	Respiration	Respiration - no subcatego	0	0	0	0	0	0	1	0
Formate dehydrogenase N alpha subunit (EC 1.2.1.2) @ selenocysteine-containing	Respiration	Electron donating reaction	0	0	0	1	0	1	0	1
Formate dehydrogenase N gamma subunit (EC 1.2.1.2)	Respiration	Electron donating reaction	0	0	0	1	0	1	0	1
Formate hydrogenlyase subunit 7	Respiration	Respiration - no subcatego	0	0	0	0	0	0	0	1
Formate hydrogenlyase transcriptional activator	Respiration	Respiration - no subcatego	0	0	0	0	1	0	0	0
Fumarate reductase flavoprotein subunit (EC 1.3.99.1)	Respiration	Electron donating reaction	0	0	1	0	0	0	1	0
fumarate reductase/succinate dehydrogenase flavoprotein domain protein	Respiration	Electron donating reaction	0	0	0	0	0	0	1	0
Hydrogenase 3 maturation protease (EC 3.4.-.-)	Respiration	Electron donating reaction	0	0	0	0	0	0	0	1
L-2-hydroxyglutarate oxidase (EC 1.1.3.15) (gaba operon, ygaF)	Respiration	Electron donating reaction	1	0	0	0	1	0	1	0

Chromosomal Genes (Plasmid-borne genes listed separately below)	SEED CATEGORY	SEED SUBCATEGORY	BR7-2h-up	BR7-2h-down	BR21-2h-up	BR21-2h-down	BR7-8h-up	BR7-8h-down	BR21-8h-up	BR21-8h-down
NAD(P)H-flavin oxidoreductase	Respiration	Electron donating reaction	0	0	0	0	0	0	1	0
NAD(P)HX epimerase / NAD(P)HX dehydratase	Respiration	Electron donating reaction	0	0	0	0	0	0	1	0
NADH dehydrogenase (EC 1.6.99.3)	Respiration	Electron donating reaction	0	0	0	0	0	1	0	1
NADH-ubiquinone oxidoreductase chain E (EC 1.6.5.3)	Respiration	Electron donating reaction	0	0	0	0	0	0	1	0
NADH-ubiquinone oxidoreductase chain K (EC 1.6.5.3)	Respiration	Electron donating reaction	0	0	0	0	0	0	1	0
NADH-ubiquinone oxidoreductase chain M (EC 1.6.5.3)	Respiration	Electron donating reaction	0	0	0	0	0	0	1	0
NADPH-dependent broad range aldehyde dehydrogenase YqhD	Respiration	Electron donating reaction	0	0	0	0	0	0	0	1
Putative cytochrome C-type biogenesis protein	Respiration	Respiration - no subcatego	0	0	0	0	0	0	0	1
Putative ion-channel protein	Respiration	Electron donating reaction	0	1	0	0	0	1	0	0
Rieske (2Fe-2S) protein (Vanillate-O-demethylase oxygenase)	Respiration	Electron donating reaction	1	0	0	0	1	0	1	0
short chain dehydrogenase	Respiration	Electron donating reaction	0	0	1	0	0	0	1	0
Short-chain dehydrogenase/reductase SDR	Respiration	Electron donating reaction	0	0	0	0	1	0	1	0
Soluble aldose sugar dehydrogenase, PQQ-dependent (EC 1.1.5.-)	Respiration	Electron donating reaction	0	0	0	0	0	0	1	0
Succinate dehydrogenase cytochrome b-556 subunit; SdhC	Respiration	Electron donating reaction	1	0	0	0	1	0	1	0
Succinate dehydrogenase hydrophobic membrane anchor protein	Respiration	Electron donating reaction	1	0	0	0	1	0	1	0
Succinate-semialdehyde dehydrogenase [NAD] (EC 1.2.1.24); Succinate-semialdeh	Respiration	Electron donating reaction	0	0	0	0	0	0	1	0
Vanillate O-demethylase oxidoreductase (EC 1.14.13.-)	Respiration	Electron accepting reactior	0	0	0	0	1	0	1	0
Vanillate O-demethylase oxygenase subunit (EC 1.14.13.82)	Respiration	Electron donating reaction	1	0	0	0	1	0	1	0
16S rRNA (guanine(966)-N(2))-methyltransferase (EC 2.1.1.171)	RNA Metabolism	RNA processing and modifi	0	0	0	0	0	0	0	1
3'-to-5' oligoribonuclease (orn)	RNA Metabolism	RNA processing and modifi	0	0	0	0	0	0	0	1
ATP-dependent 23S rRNA helicase DbpA	RNA Metabolism	RNA processing and modifi	0	0	0	0	0	0	1	0
ATP-dependent helicase HrpB	RNA Metabolism	RNA processing and modifi	0	0	0	0	0	0	0	1
ATP-dependent RNA helicase RhlE	RNA Metabolism	RNA processing and modifi	0	0	0	0	0	1	0	1
COG2197: Response regulator containing a CheY-like receiver domain and an HTH	RNA Metabolism	Transcription	0	0	0	0	0	1	0	0
DEAD-box ATP-dependent RNA helicase CshA (EC 3.6.4.13)	RNA Metabolism	RNA processing and modifi	0	0	0	1	0	1	0	1
DNA-directed RNA polymerase alpha subunit (EC 2.7.7.6)	RNA Metabolism	Transcription	0	0	0	0	0	0	0	1
FIG004454: RNA binding protein	RNA Metabolism	RNA processing and modifi	0	0	0	0	0	0	0	1
FIG136845: Rhodanese-related sulfurtransferase	RNA Metabolism	RNA processing and modifi	0	0	0	0	0	0	0	1
Iron-sulfur cluster assembly ATPase protein SufC	RNA Metabolism	RNA processing and modifi	0	0	0	0	0	0	1	0
Iron-sulfur cluster assembly protein SufB	RNA Metabolism	RNA processing and modifi	0	0	0	0	0	0	1	0
Iron-sulfur cluster assembly protein SufD	RNA Metabolism	RNA processing and modifi	0	0	0	0	0	0	1	0
O-methyltransferase	RNA Metabolism	RNA processing and modifi	0	0	0	0	0	0	1	0
Polyribonucleotide nucleotidyltransferase (EC 2.7.7.8)	RNA Metabolism	RNA processing and modifi	0	0	0	0	0	0	0	1
probable ribonuclease inhibitor YPO3690	RNA Metabolism	RNA processing and modifi	0	0	0	0	1	0	1	0
Protein export cytoplasm protein SecA ATPase RNA helicase (TC 3.A.5.1.1)	RNA Metabolism	RNA processing and modifi	0	0	0	0	0	0	0	1
Putative ATP-dependent helicase	RNA Metabolism	RNA processing and modifi	0	0	1	0	0	0	1	0
Putative preQ0 transporter	RNA Metabolism	RNA processing and modifi	0	0	0	0	0	0	1	0
Regulator of sigma D	RNA Metabolism	Transcription	0	0	0	0	1	0	1	0
Ribonuclease HI (EC 3.1.26.4)	RNA Metabolism	RNA processing and modifi	0	0	0	0	0	0	0	1
RNA polymerase sigma factor RpoS	RNA Metabolism	Transcription	0	0	0	0	1	0	1	0
rRNA small subunit methyltransferase J	RNA Metabolism	RNA processing and modifi	0	0	0	0	0	0	0	1
Transcription termination protein NusA	RNA Metabolism	Transcription	0	0	0	0	0	1	0	1
tRNA (guanosine(18)-2'-O)-methyltransferase (EC 2.1.1.34)	RNA Metabolism	RNA processing and modifi	0	0	0	0	0	0	0	1
tRNA dihydrouridine synthase B (EC 1.-.-.-)	RNA Metabolism	RNA processing and modifi	0	0	0	0	0	1	0	0
tRNA/RNA cytosine-C5-methylase (EC 2.1.1.-)	RNA Metabolism	RNA processing and modifi	0	0	0	0	0	1	0	0
Carbon starvation induced protein CsiD	Stress Response	Stress Response - no subca	1	0	0	0	1	0	1	0
Catalase (EC 1.11.1.6)	Stress Response	Oxidative stress	0	0	0	0	1	0	1	0
Cell wall endopeptidase, family M23/M37	Stress Response	Oxidative stress	0	0	0	0	0	0	1	0
Choline-glycine betaine transporter	Stress Response	Osmotic stress	0	0	0	0	1	0	1	0
Cold shock protein CspA	Stress Response	Cold shock	0	0	0	1	0	1	0	1

Chromosomal Genes (Plasmid-borne genes listed separately below)	SEED CATEGORY	SEED SUBCATEGORY	BR7-2h-up	BR7-2h-down	BR21-2h-up	BR21-2h-down	BR7-8h-up	BR7-8h-down	BR21-8h-up	BR21-8h-down
Cold shock protein CspD	Stress Response	Cold shock	1	0	0	0	1	0	1	0
DedA family inner membrane protein YdjX	Stress Response	Osmotic stress	0	0	0	0	0	1	0	1
DedA family inner membrane protein YghB	Stress Response	Osmotic stress	0	0	0	0	0	0	0	1
Entericidin B precursor; entB	Stress Response	Osmotic stress	0	0	0	0	0	0	1	0
FIG005189: putative transferase clustered with tellurite resistance proteins TehA/T	Stress Response	Detoxification	0	0	0	0	1	0	1	0
FOG: GGDEF domain	Stress Response	Stress Response - no subca	0	0	0	0	0	0	1	0
Gamma-glutamyltranspeptidase (EC 2.3.2.2)	Stress Response	Oxidative stress	0	0	0	0	1	0	1	0
General stress protein 18	Stress Response	Stress Response - no subca	0	0	0	0	1	0	1	0
GGDEF family protein	Stress Response	Stress Response - no subca	0	0	0	0	0	0	1	0
Glutathione peroxidase family protein	Stress Response	Oxidative stress	0	0	0	0	0	0	1	0
Glutathione reductase (EC 1.8.1.7)	Stress Response	Oxidative stress	0	0	0	0	0	0	0	1
Glutathione S-transferase (EC 2.5.1.18)	Stress Response	Oxidative stress	0	0	0	0	0	0	1	0
Glutathione S-transferase, omega (EC 2.5.1.18)	Stress Response	Oxidative stress	0	0	0	0	1	0	1	0
Glutathione S-transferase, theta (EC 2.5.1.18)	Stress Response	Oxidative stress	0	0	0	0	1	0	1	0
Heat shock protein hslJ	Stress Response	Heat shock	0	0	0	0	0	1	0	1
Hnr protein; rssB homolog	Stress Response	Stress Response - no subca	0	0	0	0	0	0	1	0
L-proline glycine betaine ABC transport system permease protein ProV (TC 3.A.1.1	Stress Response	Osmotic stress	0	0	0	0	0	0	1	0
L-proline glycine betaine binding ABC transporter protein ProX (TC 3.A.1.12.1)	Stress Response	Osmotic stress	0	0	0	0	0	0	1	0
Manganese catalase (EC 1.11.1.6)	Stress Response	Oxidative stress	0	0	0	0	0	0	1	0
Membrane-bound metal-dependent hydrolase YdjM, induced during SOS response	Stress Response	Stress Response - no subca	0	0	0	0	0	1	0	0
Nitrogen regulation protein NtrB (EC 2.7.13.3) (aka glnL)	Stress Response	Stress Response - no subca	0	0	0	0	0	1	0	1
Nitrogen regulation protein NtrC (aka glnG)	Stress Response	Stress Response - no subca	0	0	0	0	0	0	0	1
Non-heme chloroperoxidase (EC 1.11.1.10)	Stress Response	Oxidative stress	0	0	0	0	1	0	1	0
NtrC family Transcriptional regulator, ATPase domain	Stress Response	Stress Response - no subca	0	0	0	0	1	0	0	0
Osmoprotectant ABC transporter ATP-binding subunit YehX	Stress Response	Osmotic stress	0	0	0	0	0	0	1	0
Osmoprotectant ABC transporter binding protein YehZ	Stress Response	Osmotic stress	0	0	0	0	1	0	1	0
Osmoprotectant ABC transporter inner membrane protein YehW	Stress Response	Osmotic stress	0	0	0	0	0	0	1	0
Osmoprotectant ABC transporter permease protein YehY	Stress Response	Osmotic stress	0	0	0	0	0	0	1	0
Osmotically inducible lipoprotein B precursor: OsmB	Stress Response	Osmotic stress	0	0	0	0	0	0	1	0
Osmotically inducible lipoprotein E precursor OsmE	Stress Response	Osmotic stress	0	0	0	0	1	0	1	0
Osmotically inducible protein C OsmC	Stress Response	Osmotic stress	0	0	0	0	1	0	1	0
Osmotically inducible protein OsmY	Stress Response	Osmotic stress	0	0	0	0	1	0	1	0
Outer membrane lipoprotein blc precursor	Stress Response	Osmotic stress	0	0	0	0	1	0	1	0
Phosphate starvation-inducible protein PsiF	Stress Response	Stress Response - no subca	0	0	0	0	1	0	1	0
Probable glutathione S-transferase (EC 2.5.1.18), YfcG homolog	Stress Response	Oxidative stress	0	0	0	0	0	0	1	0
Probable protease HtpX (EC 3.4.24.-)	Stress Response	Stress Response - no subca	0	0	0	0	0	0	1	0
Protein involved in stability of MscS mechanosensitive channel	Stress Response	Osmotic stress	0	0	0	0	0	0	0	1
protein iraP	Stress Response	Stress Response - no subca	0	0	0	0	1	0	1	0
Protein sprT	Stress Response	Stress Response - no subca	0	0	0	0	0	1	0	1
Putative ThuR, regulatory protein for trehalosemaltose transport	Stress Response	Stress Response - no subca	0	0	0	0	0	0	0	1
Regulatory protein SoxS	Stress Response	Oxidative stress	0	0	1	0	0	0	0	0
Regulatory protein soxS	Stress Response	Oxidative stress	0	1	0	1	0	1	0	1
Rtn protein	Stress Response	Stress Response - no subca	0	0	0	0	0	0	1	0
Senescence marker protein-30	Stress Response	Oxidative stress	0	0	0	0	0	0	1	0
Sensory box/GGDEF family protein	Stress Response	Stress Response - no subca	0	0	0	0	0	0	1	0
Small heat shock protein	Stress Response	Heat shock	0	0	0	0	1	0	1	0
Starvation lipoprotein Slp paralog	Stress Response	Stress Response - no subca	0	0	0	0	0	0	0	1
Stationary phase inducible protein CsiE	Stress Response	Stress Response - no subca	0	0	0	0	1	0	1	0
Superoxide dismutase [Cu-Zn] precursor (EC 1.15.1.1)	Stress Response	Oxidative stress	0	0	0	0	0	0	1	0
Superoxide dismutase [Fe] (EC 1.15.1.1)	Stress Response	Oxidative stress	0	0	0	0	0	0	1	0

Chromosomal Genes (Plasmid-borne genes listed separately below)	**SEED CATEGORY**	**SEED SUBCATEGORY**	BR7-2h-up	BR7-2h-down	BR21-2h-up	BR21-2h-down	BR7-8h-up	BR7-8h-down	BR21-8h-up	BR21-8h-down
Thioredoxin 2 (EC 1.8.1.8); TrxC	Stress Response	Oxidative stress	1	0	0	0	1	0	1	0
Transcriptional activator RamA	Stress Response	Stress Response - no subca	0	0	0	0	0	0	1	0
Transcriptional regulator, IclR family	Stress Response	Stress Response - no subca	1	0	0	0	1	0	1	0
TsgA protein homolog	Stress Response	Detoxification	0	0	0	0	0	0	0	1
Uncharacterized membrane lipoprotein clustered with tellurite resistance proteins	Stress Response	Detoxification	0	0	0	0	0	0	1	0
Uncharacterized protein YqjB	Stress Response	Stress Response - no subca	0	0	0	0	0	1	0	1
Universal stress protein B	Stress Response	Stress Response - no subca	0	0	0	0	1	0	1	0
Universal stress protein F	Stress Response	Stress Response - no subca	0	0	0	0	0	0	1	0
UPF0337 protein - YjbJ-unchar general stress protein	Stress Response	Stress Response - no subca	0	0	0	0	1	0	1	0
Arylsulfatase (EC 3.1.6.1)	Sulfur Metabolism	Sulfur Metabolism - no sub	0	0	0	0	0	0	0	1
Bacterial luciferase family protein YtmO, in cluster with L-cystine ABC transporter	Sulfur Metabolism	Organic sulfur assimilation	0	0	0	0	0	0	1	0
L-cystine uptake protein TcyP	Sulfur Metabolism	Organic sulfur assimilation	0	0	0	0	0	1	0	1
Putative thiosulfate sulfurtransferase ynjE (EC 2.8.1.1)	Sulfur Metabolism	Sulfur Metabolism - no sub	0	0	0	0	0	0	0	1
ATP-dependent DNA helicase RecG (EC 3.6.1.-)	Uncharacterized	Uncharacterized	0	0	0	0	0	0	0	1
Bacterioferritin-associated ferredoxin	Uncharacterized	Uncharacterized	0	0	0	0	0	0	1	0
COG1683: Uncharacterized conserved protein / FIG143828: Hypothetical protein Y	Uncharacterized	Uncharacterized	0	0	0	1	0	0	0	0
corresponds to STY2919 from Accession AL513382: Salmonella typhi CT18	Uncharacterized	Uncharacterized	0	0	0	0	1	0	1	0
corresponds to STY3950 from Accession AL513382: Salmonella typhi CT18	Uncharacterized	Uncharacterized	0	0	0	0	0	0	1	0
Exoenzymes regulatory protein AepA precursor	Uncharacterized	uncharacterized regulatory	0	0	0	0	0	0	1	0
FIG002095: hypothetical protein	Uncharacterized	Uncharacterized	0	0	0	0	0	0	1	0
FIG002337: predicted inner membrane protein	Uncharacterized	Uncharacterized	0	0	0	0	0	0	1	0
FIG002958: hypothetical protein	Uncharacterized	Uncharacterized	0	0	0	0	0	0	0	1
FIG002994: Putative transcriptional regulator	Uncharacterized	uncharacterized regulatory	0	0	0	1	0	0	0	1
FIG004088: inner membrane protein YebE	Uncharacterized	Uncharacterized	0	0	0	0	0	1	0	0
FIG004405: Putative cytoplasmic protein	Uncharacterized	Uncharacterized	0	0	0	0	0	0	1	0
FIG00509904: hypothetical protein	Uncharacterized	Uncharacterized	0	0	0	0	0	1	0	0
FIG00510911: hypothetical protein	Uncharacterized	Uncharacterized	0	0	0	0	1	0	1	0
FIG005119: putative inner membrane protein	Uncharacterized	Uncharacterized	0	0	0	0	1	0	0	0
FIG005274: hypothetical protein	Uncharacterized	Uncharacterized	0	0	0	0	0	0	1	0
FIG00543870: hypothetical protein	Uncharacterized	Uncharacterized	0	0	0	0	1	0	1	0
FIG00554739: membrane protein YchH	Uncharacterized	Uncharacterized	0	0	0	0	0	0	1	0
FIG00613201: hypothetical protein	Uncharacterized	Uncharacterized	0	0	0	0	0	0	0	1
FIG00626754: hypothetical protein	Uncharacterized	Uncharacterized	1	0	0	0	1	0	1	0
FIG00637864: hypothetical protein	Uncharacterized	Uncharacterized	0	0	0	0	0	0	1	0
FIG00638451: hypothetical protein yiaG putative transcriptional regulator	Uncharacterized	Uncharacterized	0	0	0	0	1	0	1	0
FIG00638667: hypothetical protein	Uncharacterized	Uncharacterized	1	0	0	0	1	0	1	0
FIG00639224: hypothetical protein	Uncharacterized	Uncharacterized	0	0	0	0	0	0	1	0
FIG00641944: hypothetical protein	Uncharacterized	Uncharacterized	0	0	0	0	0	0	1	0
FIG00644304: hypothetical protein	Uncharacterized	Uncharacterized	0	0	0	1	0	0	0	0
FIG00731334: hypothetical protein	Uncharacterized	Uncharacterized	0	0	0	0	0	0	1	0
FIG00731341: hypothetical protein	Uncharacterized	Uncharacterized	0	0	0	0	0	1	1	0
FIG00731353: hypothetical protein	Uncharacterized	Uncharacterized	0	0	0	0	0	0	1	0
FIG00731379: hypothetical protein	Uncharacterized	Uncharacterized	0	0	0	0	0	0	1	0
FIG00731387: hypothetical protein-YgaU	Uncharacterized	Uncharacterized	0	0	0	0	1	0	1	0
FIG00731389: hypothetical protein	Uncharacterized	Uncharacterized	0	0	0	0	0	0	0	1
FIG00731406: hypothetical protein	Uncharacterized	Uncharacterized	0	0	0	0	0	0	1	0
FIG00731415: hypothetical protein	Uncharacterized	Uncharacterized	0	0	1	0	0	0	1	0
FIG00731458: hypothetical protein	Uncharacterized	Uncharacterized	0	0	0	0	0	0	0	1
FIG00731504: hypothetical protein	Uncharacterized	Uncharacterized	0	0	0	0	1	0	0	0
FIG00731529: hypothetical protein	Uncharacterized	Uncharacterized	0	0	0	0	0	1	0	0

Chromosomal Genes (Plasmid-borne genes listed separately below)	**SEED CATEGORY**	**SEED SUBCATEGORY**	BR7-2h-up	BR7-2h-down	BR21-2h-up	BR21-2h-down	BR7-8h-up	BR7-8h-down	BR21-8h-up	BR21-8h-down
FIG00731538: hypothetical protein	Uncharacterized	Uncharacterized	0	0	0	0	1	0	1	0
FIG00731566: hypothetical protein	Uncharacterized	Uncharacterized	0	0	0	0	0	0	1	0
FIG00731571: hypothetical protein	Uncharacterized	Uncharacterized	0	0	0	0	1	0	1	0
FIG00731668: hypothetical protein	Uncharacterized	Uncharacterized	0	0	0	0	0	0	1	0
FIG00731698: hypothetical protein	Uncharacterized	Uncharacterized	0	0	0	0	0	0	0	1
FIG00731703: hypothetical protein	Uncharacterized	Uncharacterized	0	0	0	0	0	1	0	0
FIG00731712: hypothetical protein	Uncharacterized	Uncharacterized	0	0	0	0	0	0	0	1
FIG00731716: hypothetical protein	Uncharacterized	Uncharacterized	0	0	0	0	0	0	0	1
FIG00731791: hypothetical protein	Uncharacterized	Uncharacterized	0	0	0	0	0	1	0	0
FIG00731810: hypothetical protein	Uncharacterized	Uncharacterized	0	0	0	0	0	0	0	1
FIG00731846: hypothetical protein	Uncharacterized	Uncharacterized	0	0	0	0	1	0	1	0
FIG00731878: hypothetical protein	Uncharacterized	Uncharacterized	0	0	0	0	0	0	1	0
FIG00731881: hypothetical protein	Uncharacterized	Uncharacterized	0	0	1	0	0	0	1	0
FIG00731925: hypothetical protein-Two-component-system connector protein Ari	Uncharacterized	Uncharacterized	0	0	0	0	0	0	1	0
FIG00731973: hypothetical protein	Uncharacterized	Uncharacterized	0	0	0	0	0	1	0	1
FIG00732023: hypothetical protein	Uncharacterized	Uncharacterized	0	0	0	0	0	0	0	1
FIG00732033: hypothetical protein	Uncharacterized	Uncharacterized	0	0	0	0	0	0	1	0
FIG00732054: hypothetical protein	Uncharacterized	Uncharacterized	0	0	0	0	0	0	0	1
FIG00732056: hypothetical protein	Uncharacterized	Uncharacterized	0	0	0	0	0	1	0	0
FIG00732091: hypothetical protein	Uncharacterized	Uncharacterized	0	0	0	0	0	0	0	1
FIG00732127: hypothetical protein	Uncharacterized	Uncharacterized	0	0	0	0	0	0	1	0
FIG00732133: hypothetical protein	Uncharacterized	Uncharacterized	0	0	0	0	0	0	1	0
FIG00732166: hypothetical protein	Uncharacterized	Uncharacterized	0	0	0	0	1	0	1	0
FIG00732195: hypothetical protein	Uncharacterized	Uncharacterized	0	0	0	0	0	0	1	0
FIG00732247: hypothetical protein	Uncharacterized	Uncharacterized	1	0	0	0	1	0	1	0
FIG00732255: hypothetical protein	Uncharacterized	Uncharacterized	0	0	0	0	1	0	1	0
FIG00732265: hypothetical protein	Uncharacterized	Uncharacterized	0	0	0	0	1	0	1	0
FIG00732296: hypothetical protein	Uncharacterized	Uncharacterized	0	0	0	0	0	0	1	0
FIG00732313: hypothetical protein	Uncharacterized	Uncharacterized	0	0	0	0	0	0	1	0
FIG00732337: hypothetical protein	Uncharacterized	Uncharacterized	0	0	0	1	0	0	0	0
FIG00732338: hypothetical protein - YgaM	Uncharacterized	Uncharacterized	0	0	0	0	1	0	1	0
FIG00732344: hypothetical protein	Uncharacterized	Uncharacterized	0	0	0	0	0	0	0	1
FIG00732364: hypothetical protein	Uncharacterized	Uncharacterized	0	0	0	0	0	0	1	0
FIG00732374: hypothetical protein	Uncharacterized	Uncharacterized	0	0	0	0	0	0	1	0
FIG00732415: hypothetical protein	Uncharacterized	Uncharacterized	0	0	0	0	0	0	1	0
FIG00732462: hypothetical protein	Uncharacterized	Uncharacterized	0	0	0	0	0	1	0	0
FIG00732463: hypothetical protein	Uncharacterized	Uncharacterized	0	0	1	0	0	0	1	0
FIG00732473: hypothetical protein	Uncharacterized	Uncharacterized	0	0	0	0	0	0	0	1
FIG00732486: hypothetical protein	Uncharacterized	Uncharacterized	0	0	0	0	0	0	1	0
FIG00732487: hypothetical protein	Uncharacterized	Uncharacterized	0	0	0	0	0	0	0	1
FIG00732545: hypothetical protein	Uncharacterized	Uncharacterized	0	0	0	0	1	0	1	0
FIG00732562: hypothetical protein	Uncharacterized	Uncharacterized	0	1	0	0	0	1	0	0
FIG00732604: hypothetical protein	Uncharacterized	Uncharacterized	0	0	0	0	1	0	1	0
FIG00732653: hypothetical protein	Uncharacterized	Uncharacterized	0	0	0	0	0	0	1	0
FIG00732736: hypothetical protein	Uncharacterized	Uncharacterized	0	0	0	0	1	0	1	0
FIG00732740: hypothetical protein	Uncharacterized	Uncharacterized	0	0	0	0	0	0	0	1
FIG00732763: hypothetical protein	Uncharacterized	Uncharacterized	0	0	0	0	0	0	1	0
FIG00732790: hypothetical protein	Uncharacterized	Uncharacterized	0	0	0	0	0	0	1	0
FIG00732796: hypothetical protein	Uncharacterized	Uncharacterized	0	0	0	0	0	0	0	1
FIG00732835: hypothetical protein	Uncharacterized	Uncharacterized	0	0	0	0	1	0	1	0

Chromosomal Genes (Plasmid-borne genes listed separately below)	**SEED CATEGORY**	**SEED SUBCATEGORY**	BR7-2h-up	BR7-2h-down	BR21-2h-up	BR21-2h-down	BR7-8h-up	BR7-8h-down	BR21-8h-up	BR21-8h-down
FIG00732856: hypothetical protein	Uncharacterized	Uncharacterized	0	0	0	0	0	1	0	0
FIG00732920: hypothetical protein	Uncharacterized	Uncharacterized	0	0	0	0	1	0	1	0
FIG00732925: hypothetical protein	Uncharacterized	Uncharacterized	0	0	0	0	0	0	0	1
FIG00732927: hypothetical protein	Uncharacterized	Uncharacterized	0	0	0	0	0	0	1	0
FIG00809136: hypothetical protein	Uncharacterized	Uncharacterized	0	0	0	0	0	0	1	0
FIG00919961: hypothetical protein	Uncharacterized	Uncharacterized	0	0	0	0	1	0	1	0
FIG00921183: possible ligase	Uncharacterized	Uncharacterized	0	0	0	0	0	0	0	1
FIG00922551: hypothetical protein	Uncharacterized	Uncharacterized	0	0	0	0	0	0	0	1
FIG01199806: hypothetical protein	Uncharacterized	Uncharacterized	0	0	0	0	0	0	1	0
FIG01200701: possible membrane protein	Uncharacterized	Uncharacterized	0	0	0	0	1	0	1	0
FIG01219827: hypothetical protein - ygaC	Uncharacterized	Uncharacterized	0	0	0	0	1	0	1	0
FIG01220476: hypothetical protein	Uncharacterized	Uncharacterized	0	0	0	0	1	0	1	0
FIG137360: hypothetical protein	Uncharacterized	Uncharacterized	0	0	0	0	0	0	1	0
FIG139552: Putative protease	Uncharacterized	Uncharacterized	0	0	0	0	0	0	0	1
FIG139928: Putative protease	Uncharacterized	Uncharacterized	0	0	0	0	0	0	0	1
FxsA protein	Uncharacterized	Uncharacterized	0	0	0	0	0	0	0	1
Glycosaminoglycan attachment site	Uncharacterized	uncharacterized	0	0	0	0	1	0	0	0
Hypothetical protein COG3496	Uncharacterized	Uncharacterized	0	0	0	0	0	0	0	1
Hypothetical protein ycgF	Uncharacterized	uncharacterized	0	0	0	0	1	0	1	0
Hypothetical protein YqcC (clustered with tRNA pseudouridine synthase C)	Uncharacterized	Uncharacterized	0	0	0	0	0	1	0	1
Hypothetical Zinc-finger containing protein	Uncharacterized	Uncharacterized	0	0	0	0	0	0	1	0
Inner membrane protein YbhQ	Uncharacterized	uncharacterized	0	0	0	0	1	0	1	0
Inner membrane protein YccF	Uncharacterized	Uncharacterized	0	0	0	0	0	0	0	1
Inner membrane protein YfiN	Uncharacterized	Uncharacterized	0	0	0	0	0	0	0	1
Inner membrane protein YphA	Uncharacterized	Uncharacterized	0	0	0	0	0	0	1	0
Inner membrane protein YqjE	Uncharacterized	Uncharacterized	0	0	0	0	0	0	1	0
Inner membrane protein YqjK	Uncharacterized	Uncharacterized	0	0	0	0	1	0	1	0
LysR-family transcriptional regulator	Uncharacterized	uncharacterized regulatory	0	0	0	0	0	0	0	1
membrane protein, putative	Uncharacterized	Uncharacterized	0	0	0	0	0	0	1	0
MG(2+) CHELATASE FAMILY PROTEIN / ComM-related protein	Uncharacterized	uncharacterized	0	0	0	0	1	0	1	0
Possible membrane transport protein	Uncharacterized	Uncharacterized	0	0	0	0	0	0	1	0
Probable 3-phenylpropionic acid transporter	Uncharacterized	uncharacterized	0	0	0	0	0	1	0	1
Probable ABC transporter, ATP-binding subunit	Uncharacterized	Uncharacterized	1	0	0	0	1	0	1	0
probable exported protein YPO0130	Uncharacterized	uncharacterized	0	0	0	0	1	0	1	0
probable exported protein YPO4040	Uncharacterized	Uncharacterized	0	0	0	1	0	0	0	0
probable extracellular solute-binding protein	Uncharacterized	uncharacterized	0	0	0	0	0	0	1	0
probable lipoprotein- ybjP	Uncharacterized	Uncharacterized	0	0	0	0	0	0	1	0
probable membrane protein yjeI	Uncharacterized	Uncharacterized	0	0	0	0	1	0	0	0
probable regulatory protein STY1856	Uncharacterized	Uncharacterized	0	0	0	0	0	0	1	0
Probable secreted protein	Uncharacterized	uncharacterized	0	0	0	0	1	0	1	0
protein	Uncharacterized	uncharacterized	0	0	0	0	1	0	1	0
Protein YaiA	Uncharacterized	Uncharacterized	0	0	0	0	0	0	1	0
Protein YciE	Uncharacterized	Uncharacterized	0	0	0	0	1	0	1	0
Protein YciF	Uncharacterized	Uncharacterized	0	0	0	0	0	0	1	0
Protein yhjK	Uncharacterized	Uncharacterized	0	0	0	0	0	0	0	1
putative cytoplasmic protein	Uncharacterized	Uncharacterized	0	0	0	0	0	0	1	0
Putative ECA polymerase (may be wzy, regulator of mucoid capsule)	Uncharacterized	Uncharacterized	0	0	0	0	0	0	0	1
Putative esterase	Uncharacterized	Uncharacterized	0	0	0	0	1	0	1	0
Putative exported protein	Uncharacterized	Uncharacterized	0	0	0	0	0	0	0	1
Putative exported protein precursor	Uncharacterized	Uncharacterized	0	0	0	0	0	0	1	0

Chromosomal Genes (Plasmid-borne genes listed separately below)	SEED CATEGORY	SEED SUBCATEGORY	BR7-2h-up	BR7-2h-down	BR21-2h-up	BR21-2h-down	BR7-8h-up	BR7-8h-down	BR21-8h-up	BR21-8h-down
putative fimbrial-like protein	Uncharacterized	Uncharacterized	0	0	0	0	0	0	0	1
putative glutamine amidotransferase class-I	Uncharacterized	Uncharacterized	0	0	0	0	0	0	1	0
Putative glycoporin	Uncharacterized	Uncharacterized	0	0	0	0	0	0	1	0
Putative glycoprotein/receptor	Uncharacterized	Uncharacterized	0	0	0	0	0	0	0	1
putative inner membrane protein	Uncharacterized	Uncharacterized	0	0	0	0	0	0	1	0
putative integral membrane transport protein	Uncharacterized	Uncharacterized	1	0	0	0	1	0	1	0
putative lipoprotein	Uncharacterized	Uncharacterized	0	0	0	0	0	0	1	0
Putative membrane protein YeiH	Uncharacterized	uncharacterized	0	0	0	0	0	1	0	1
Putative protease	Uncharacterized	Uncharacterized	0	0	0	0	0	1	0	0
Putative regulator	Uncharacterized	uncharacterized regulatory	0	0	0	0	1	0	1	0
putative transport	Uncharacterized	Uncharacterized	0	0	0	0	0	0	1	0
regulatory protein, LuxR	Uncharacterized	uncharacterized regulatory	0	0	0	0	0	0	1	0
Response regulator protein Z5684	Uncharacterized	uncharacterized	0	0	0	0	1	0	0	0
Transcriptional regulator PobR, AraC family	Uncharacterized	Uncharacterized	1	0	0	0	1	0	1	0
Uncharacterised protein family UPF0157 (COG2320)	Uncharacterized	Uncharacterized	0	0	0	0	0	0	1	0
Uncharacterized lipoprotein yaeF precursor	Uncharacterized	Uncharacterized	0	0	0	0	0	1	0	1
Uncharacterized lipoprotein YsaB precursor	Uncharacterized	Uncharacterized	0	0	0	0	0	0	1	0
Uncharacterized membrane protein YqjD	Uncharacterized	uncharacterized	0	0	0	0	1	0	1	0
Uncharacterized oxidoreductase ydgJ (EC 1.-.-.-)	Uncharacterized	Uncharacterized	0	0	0	0	0	0	1	0
Uncharacterized PLP-dependent aminotransferase YfdZ	Uncharacterized	Uncharacterized	0	0	0	0	1	0	0	0
Uncharacterized protein conserved in bacteria	Uncharacterized	Uncharacterized	0	0	0	0	0	0	1	0
Uncharacterized protein ybfE	Uncharacterized	uncharacterized	0	0	0	0	0	1	0	0
Uncharacterized protein YhaL	Uncharacterized	Uncharacterized	0	0	0	0	0	0	0	1
UPF0098 protein ybhB	Uncharacterized	Uncharacterized	0	0	0	0	0	0	1	0
YbjC protein, clustered with oxygen-insensitive NADPH nitroreductase	Uncharacterized	uncharacterized	0	0	0	0	0	1	0	0
Albicidin resistance protein	Virulence, Disease and Defense	Resistance to antibiotics ar	0	0	0	0	0	0	1	0
Antibiotic biosynthesis monooxygenase	Virulence, Disease and Defense	Resistance to antibiotics ar	0	0	0	0	0	0	1	0
Arsenic efflux pump protein	Virulence, Disease and Defense	Resistance to antibiotics ar	0	0	0	0	0	0	1	0
Gifsy-2 prophage protein	Virulence, Disease and Defense	Resistance to antibiotics ar	0	0	0	0	1	0	1	0
HTH-type transcriptional regulator YidP	Virulence, Disease and Defense	Adhesion	0	0	0	0	0	0	0	1
Metal-dependent hydrolases of the beta-lactamase superfamily III	Virulence, Disease and Defense	Resistance to antibiotics ar	0	0	0	0	0	0	1	0
Methyl viologen resistance protein smvA	Virulence, Disease and Defense	Resistance to antibiotics ar	0	0	0	1	0	0	1	0
Multidrug and toxin extrusion (MATE) family efflux pump YdhE/NorM	Virulence, Disease and Defense	Resistance to antibiotics ar	0	0	0	0	0	0	1	0
Multidrug-efflux transporter, major facilitator superfamily (MFS) (TC 2.A.1)	Virulence, Disease and Defense	Resistance to antibiotics ar	0	0	0	0	0	0	0	1
Outer membrane component of tripartite multidrug resistance system	Virulence, Disease and Defense	Resistance to antibiotics ar	0	0	0	0	0	0	0	1
Probable Co/Zn/Cd efflux system membrane fusion protein	Virulence, Disease and Defense	Resistance to antibiotics ar	0	0	0	0	0	1	0	0
Putative resistance protein	Virulence, Disease and Defense	Resistance to antibiotics ar	0	0	0	0	0	1	0	1
Quaternary ammonium compound-resistance protein SugE	Virulence, Disease and Defense	Resistance to antibiotics ar	0	0	0	0	0	0	1	0
RND efflux system, membrane fusion protein CmeA (oqxA)	Virulence, Disease and Defense	Resistance to antibiotics ar	0	0	0	0	0	1	0	0
RND multidrug efflux transporter; Acriflavin resistance protein; oqxB	Virulence, Disease and Defense	Resistance to antibiotics ar	0	0	0	0	0	1	0	1
SbmA protein	Virulence, Disease and Defense	Resistance to antibiotics ar	0	0	0	0	0	0	0	1
Sensor protein of zinc sigma-54-dependent two-component system	Virulence, Disease and Defense	Resistance to antibiotics ar	0	0	0	0	0	0	0	1
Spermidine export protein MdtI	Virulence, Disease and Defense	Resistance to antibiotics ar	0	0	0	1	0	0	0	0
Spermidine export protein MdtJ	Virulence, Disease and Defense	Resistance to antibiotics ar	0	0	0	1	0	0	0	0
Uncharacterized protein YidR	Virulence, Disease and Defense	Adhesion	0	0	0	0	0	0	0	1
virulence protein	Virulence, Disease and Defense	Virulence - no subcategory	0	0	1	0	0	0	0	0
Zinc ABC transporter, inner membrane permease protein ZnuB	Virulence, Disease and Defense	Resistance to antibiotics ar	0	0	0	0	0	1	0	1
Zinc transport protein ZntB	Virulence, Disease and Defense	Resistance to antibiotics ar	0	0	0	0	1	0	1	0
Zinc transporter ZitB	Virulence, Disease and Defense	Resistance to antibiotics ar	0	0	0	0	0	0	1	0
unknown gene - what happened in sort?			0	0	0	0	0	0	1	0

Chromosomal Genes (Plasmid-borne genes listed separately below)	**SEED CATEGORY**	**SEED SUBCATEGORY**	BR7-2h-up	BR7-2h-down	BR21-2h-up	BR21-2h-down	BR7-8h-up	BR7-8h-down	BR21-8h-up	BR21-8h-down
Total Differentially expressed chromosomal genes			87	40	93	33	300	159	662	347
				127		126		459		1009

Plasmid-borne DE genes	**Plasmid**	BR7-2h-up	BR7-2h-down	BR21-2h-up	BR21-2h-down	BR7-8h-up	BR7-8h-down	BR21-8h-up	BR21-8h-down
Gifsy-2 prophage protein	pQIL/N		1						
Dihydrofolate reductase (EC 1.5.1.3)	IncN				1				
Error-prone repair protein UmuD	IncN			1				1	
Error-prone, lesion bypass DNA polymerase V (UmuC)	IncN			1				1	
ATPase provides energy for both assembly of type IV secretion complex and secretion of T-DNA complex (VirB4)	IncN							1	
Chloramphenicol acetyltransferase (EC 2.3.1.28)	IncN								1
DNA-cytosine methyltransferase (EC 2.1.1.37)	IncN								1
Forms the bulk of type IV secretion complex that spans outer membrane and perip	IncN							1	
Inner membrane protein forms channel for type IV secretion of T-DNA complex, Vi	IncN							1	
Minor pilin of type IV secretion complex, VirB5	IncN							1	
Peptidoglycan hydrolase VirB1, involved in T-DNA transfer	IncN							1	
TniB NTP-binding protein	IncN							1	
Type I restriction-modification system, DNA-methyltransferase subunit M (EC 2.1.1	IncN								1
Type I restriction-modification system, restriction subunit R (EC 3.1.21.3)	IncN								1
Type I restriction-modification system, specificity subunit S (EC 3.1.21.3)	IncN								1
Cation efflux system protein CusC precursor	IncF							1	
Fe2+-dicitrate sensor, membrane component	IncF							1	
FIG00732614: hypothetical protein	IncF							1	
FIG074102: hypothetical protein	IncF							1	
Iron(III) dicitrate transmembrane sensor protein FecR	IncF							1	
Lead, cadmium, zinc and mercury transporting ATPase (EC 3.6.3.3) (EC 3.6.3.5); Copper-translocating P-type ATPase (EC 3.6.3.4)	IncF							1	
PsiA protein	IncF							1	
Single-stranded DNA-binding protein	IncF							1	
Transcriptional repressor of the lac operon	IncF								1
Total Differentially expressed plasmid-borne genes		0	1	2	1	0	0	16	6

**Total raw reads, HiSeq 4000, for biological replicates A, B, C**										
	control (unexposed)			imipenem exposure, hours (h):						
	A	B	C	2h A	2h B	2h B	8h A	8h B	8h C	Total
BR7 total reads	21,553,184	24,832,896	17,320,804	18,987,301	16,851,132	16,843,007	15,163,954	19,088,601	22,445,491	173,086,370
BR21 total reads	14,710,337	19,457,058	15,829,704	16,089,259	18,349,660	19,761,198	21,244,274	18,113,567	27,445,468	171,000,525
										344,086,895

Generally Applicable Gene-set Enrichment (GAGE) analysis output

**K. pneumoniae BR7**	stat.mean	set.size	p.up	p.dn	p.val	q.val (< 0.2)
ko00330 Arginine and proline metabolism	3.161341	12	3.21E-05	0.999968	6.42E-05	0.000706
ko02010 ABC transporters	2.204952	36	0.001135	0.998865	0.00227	0.012483
ko01100 Metabolic pathways	1.412618	95	0.023452	0.976548	0.046904	0.155349
ko01200 Carbon metabolism	1.383861	18	0.028245	0.971755	0.056491	0.155349
ko02020 Two-component system	-1.28143	28	0.962678	0.037322	0.074645	0.164219

ko00330 Arginine and proline metabolism			logFC	logFC
all.mapped	all.mappec	type	BR7_2h	BR7_8h
K00137	**prr**	ortholog	0	1.541642
K00657	**speG**	ortholog	0	-1.11859
K00673	**astA**	ortholog	3.501754	4.784275
K00840	**astC**	ortholog	3.965324	5.250075
K01484	NA	ortholog	3.5502	4.249296
K01485	NA	ortholog	0	1.278882
K05526	**astE**	ortholog	3.409243	4.016197
K06447	**astD**	ortholog	3.721411	4.545581
K09251	**patA**	ortholog	0	1.551894
K09470	**puuA**	ortholog	4.079344	5.754031
K09471	**puuB**	ortholog	2.677286	3.58963
K09473	**puuD**	ortholog	3.843377	4.921032

ko02010 ABC transporters			logFC	logFC
all.mapped	all.mappec	type	BR7_2h	BR7_8h
K01999	NA	ortholog	0	1.123255
K02010	NA	ortholog	3.571517	6.108331
K02011	NA	ortholog	4.163489	7.083557
K02013	NA	ortholog	0	-2.6009
K02015	NA	ortholog	0	-1.78687
K02016	NA	ortholog	3.588766	-2.39753
K02045	NA	ortholog	-1.3046	0
K02064	NA	ortholog	0	1.187239
K05776	modF	ortholog	-1.82278	-1.15664

K05813	**ugpB**	ortholog	2.843076	4.211995
K05814	**ugpA**	ortholog	3.474596	4.651362
K05815	**ugpE**	ortholog	3.775425	4.643997
K05816	**ugpC**	ortholog	2.162543	2.905471
K05845	opuC	ortholog	0	1.620222
K10001	**gltI**	ortholog	2.713762	4.291698
K10002	**gltK**	ortholog	1.777406	2.832066
K10004	**gltL**	ortholog	1.769924	3.23687
K10013	ABC.ARG.S	ortholog	2.337383	2.983401
K10017	**hisP**	ortholog	0	1.225855
K10017	**hisP**	ortholog	0	1.225855
K10108	malE	ortholog	0	2.233418
K10110	malG	ortholog	0	1.848861
K10111	malK	ortholog	0	2.216643
K10111	malK	ortholog	0	2.216643
K10111	malK	ortholog	0	2.216643
K10111	malK	ortholog	0	2.216643
K10439	rbsB	ortholog	0	1.383187
K10542	mglA	ortholog	0	1.44957
K10543	xylF	ortholog	0	2.202788
K10545	xylG	ortholog	0	2.545314
K10555	lsrB	ortholog	0	1.98796
K10556	lsrC	ortholog	0	1.995339
K10557	lsrD	ortholog	0	1.875174
K10558	lsrA	ortholog	0	2.53526
K11073	NA	ortholog	0	1.619942
K15584	NA	ortholog	-1.74586	-1.62718
K15587	NA	ortholog	0	-1.43573
K17202	NA	ortholog	0	2.707032
K17214	NA	ortholog	2.412615	3.206202
K17215	NA	ortholog	3.052927	3.962222

ko02020 Two-component system			logFC	logFC
all.mapped	all.mappec	type	BR7_2h	BR7_8h

K00370	narG	ortholog	-3.01301	-3.93945
K00370	narG	ortholog	-3.01301	-3.93945
K00371	narH	ortholog	-2.83566	-3.73085
K00371	narH	ortholog	-2.83566	-3.73085
K00373	narJ	ortholog	0	-2.60582
K00373	narJ	ortholog	0	-2.60582
K01077	NA	ortholog	0	1.542812
K01077	NA	ortholog	0	1.542812
K01546	NA	ortholog	0	-2.17518
K01643	NA	ortholog	0	1.684622
K01644	NA	ortholog	0	2.13491
K01915	NA	ortholog	0	-1.50586
K07644	cusS	ortholog	0	-1.33631
K07657	phoB	ortholog	0	1.028626
K07667	kdpE	ortholog	0	-1.0523
K07673	narX	ortholog	-1.87461	-2.9336
K07675	uhpB	ortholog	0	-1.3759
K07679	evgS	ortholog	0	-1.47879
K07684	narL	ortholog	-2.06362	-2.69339
K07690	evgA	ortholog	0	-1.92905
K07700	dpiB	ortholog	0	1.496685
K07702	dpiA	ortholog	0	2.125148
K07708	glnL	ortholog	0	-1.21303
K07783	uhpC	ortholog	0	-1.32807
K07792	dcuB	ortholog	2.352333	0
K08348	fdnG	ortholog	0	-3.11328
K08350	fdnI	ortholog	0	-3.4788
K10001	**gltI**	ortholog	2.713762	4.291698
K10002	**gltK**	ortholog	1.777406	2.832066
K10004	**gltL**	ortholog	1.769924	3.23687
K11103	NA	ortholog	0	3.144914
K11103	NA	ortholog	0	3.144914
K19611	NA	ortholog	0	-3.40718

**K. pneumoniae BR21**	stat.mean	set.size	p.up	p.dn	p.val	q.val (< 0.2)
ko00330 Arginine and proline metabolism	2.77726	10	0.00116	0.99884	0.00232	0.025516
ko02010 ABC transporters	1.887481	72	0.004374	0.995626	0.008749	0.064156
ko00020 Citrate cycle (TCA cycle)	1.630363	17	0.012855	0.987145	0.025709	0.099563
ko00720 Carbon fixation pathways in prokaryotes	1.626157	14	0.013577	0.986423	0.027154	0.099563
ko01220 Degradation of aromatic compounds	1.53334	11	0.029983	0.970017	0.059967	0.164909
ko01130 Biosynthesis of antibiotics	1.503717	50	0.017729	0.982271	0.035458	0.11144
ko01100 Metabolic pathways	1.268082	173	0.036784	0.963216	0.073568	0.179832
ko02020 Two-component system	-1.65247	35	0.989187	0.010813	0.021627	0.099563
ko03010 Ribosome	-3.08061	35	0.999905	9.47E-05	0.000189	0.004167

ko00330 Arginine and proline metabolism			logFC	logFC
all.mapped	all.mappec	type	BR21_2h	BR21_8h
K00137	prr	ortholog	0	2.41256
K00673	**astA**	ortholog	1.497134	5.387309
K00840	**astC**	ortholog	1.624573	5.891437
K01484	NA	ortholog	0	4.163549
K05526	**astE**	ortholog	0	4.547433
K06447	**astD**	ortholog	0	4.99648
K09251	**patA**	ortholog	0	3.777509
K09470	**puuA**	ortholog	0	4.850527
K09471	**puuB**	ortholog	0	4.694586
K09473	**puuD**	ortholog	0	4.153217

ko02010 ABC transporters			logFC	logFC
all.mapped	all.mappec	type	BR21_2h	BR21_8h
K02000	NA	ortholog	0	1.137761
K02002	NA	ortholog	0	1.447073
K02010	NA	ortholog	0	4.727739
K02011	NA	ortholog	0	4.43488
K02012	NA	ortholog	0	1.68602
K02013	NA	ortholog	2.734153	2.300644
K02015	NA	ortholog	2.054811	2.906352

K02016	NA	ortholog	0	1.937694
K02020	NA	ortholog	-1.75349	0
K02041	NA	ortholog	0	1.758778
K02044	NA	ortholog	0	2.137632
K02064	NA	ortholog	0	1.155641
K05776	modF	ortholog	-1.46995	-1.78261
K05813	**ugpB**	ortholog	0	4.77258
K05814	**ugpA**	ortholog	0	4.936252
K05815	**ugpE**	ortholog	0	3.400297
K05816	**ugpC**	ortholog	0	3.713979
K05845	opuC	ortholog	0	2.903994
K05846	opuBD	ortholog	0	2.654145
K05847	opuA	ortholog	0	2.629667
K06074	ABC.VB12.	ortholog	0	0.887823
K06858	ABC.VB12.	ortholog	0	-1.0694
K09812	ftsE	ortholog	0	-1.06225
K10000	artP	ortholog	0	1.204521
K10001	**gltI**	ortholog	0	4.905064
K10002	**gltK**	ortholog	0	3.193442
K10004	**gltL**	ortholog	0	3.177999
K10013	**ABC.ARG.S**	ortholog	0	3.087819
K10036	glnH	ortholog	0	1.71233
K10108	malE	ortholog	0	-2.85976
K10109	malF	ortholog	0	-2.71372
K10110	malG	ortholog	0	-2.819
K10111	malK	ortholog	0	-3.17516
K10111	malK	ortholog	0	-3.17516
K10111	malK	ortholog	0	-3.17516
K10111	malK	ortholog	0	-3.17516
K10192	togB	ortholog	0	1.832778
K10194	togN	ortholog	0	1.508102
K10195	togA	ortholog	0	1.640071
K10439	rbsB	ortholog	1.777289	2.392902
K10537	araF	ortholog	0	2.066787

K10538	araH	ortholog	0	1.959347
K10539	araG	ortholog	0	2.288733
K10540	mglB	ortholog	0	4.988405
K10541	mglC	ortholog	1.882046	4.877401
K10542	mglA	ortholog	0	4.792101
K10543	xylF	ortholog	0	3.112318
K10545	xylG	ortholog	0	2.502781
K10555	lsrB	ortholog	0	4.873514
K10556	lsrC	ortholog	0	4.263863
K10557	lsrD	ortholog	0	4.728144
K10558	lsrA	ortholog	0	4.337619
K10824	NA	ortholog	0	-1.93188
K11073	NA	ortholog	0	2.188393
K11075	NA	ortholog	0	1.588352
K11076	NA	ortholog	0	1.527297
K11604	NA	ortholog	0	3.227928
K11605	NA	ortholog	0	2.795945
K11606	NA	ortholog	0	2.851927
K11607	NA	ortholog	2.078981	3.318698
K12368	NA	ortholog	0	2.018915
K12369	NA	ortholog	0	3.362986
K12370	NA	ortholog	0	1.391521
K12371	NA	ortholog	0	1.343125
K12372	NA	ortholog	0	1.849447
K13890	NA	ortholog	0	1.510861
K15580	NA	ortholog	0	1.774647
K15584	NA	ortholog	0	-1.98358
K15585	NA	ortholog	0	-1.90451
K15586	NA	ortholog	0	-2.44308
K15598	NA	ortholog	0	2.371422
K15599	NA	ortholog	0	3.045289
K17213	NA	ortholog	0	4.360152
K17214	NA	ortholog	0	3.005826
K17215	NA	ortholog	0	4.480829

ko00020 Citrate cycle (TCA cycle)			logFC	logFC
all.mapped	all.mappec	type	BR21_2h	BR21_8h
K00024	mdh	ortholog	0	3.784824
K00116	**mqo**	ortholog	0	2.648283
K00163	aceE	ortholog	0	-1.95903
K00163	aceE	ortholog	0	-1.95903
K00164	**OGDH**	ortholog	0	1.93609
K00164	**OGDH**	ortholog	0	1.93609
K00241,K00242	**sdhC,sdhD**	ortholog	0	4.652144
K00241,K00242,K00244,K00245	**sdhC,sdhD**	ortholog	4.957782	10.99067
K00627	DLAT	ortholog	0	-2.02198
K00658	DLST	ortholog	0	1.850095
K01610	NA	ortholog	0	3.582135
K01647	NA	ortholog	0	2.555326
K01676,K01679	NA,NA	ortholog	3.674221	7.217046
K01681	NA	ortholog	0	2.174151
K01681	NA	ortholog	0	2.174151
K01902,K01903	NA,NA	ortholog	1.510958	3.698619

ko00720 Carbon fixation pathways in prokaryotes			logFC	logFC
all.mapped	all.mappec	type	BR21_2h	BR21_8h
K00024	**mdh**	ortholog	0	3.784824
K00241,K00242	**sdhC,sdhD**	ortholog	0	4.652144
K00241,K00242,K00244,K00245	**sdhC,sdhD**	ortholog	4.957782	10.99067
K01007	NA	ortholog	0	2.333941
K01595	NA	ortholog	0	-1.32565
K01676,K01679	NA,NA	ortholog	3.674221	7.217046
K01681	NA	ortholog	0	2.174151
K01681	NA	ortholog	0	2.174151
K01895	NA	ortholog	0	5.179662
K01902,K01903	NA,NA	ortholog	1.510958	3.698619
K13788	NA	ortholog	0	-1.92215

ko02020 Two-component system			logFC	logFC
all.mapped	all.mappec	type	BR21_2h	BR21_8h
K00244	frdA	ortholog	4.957782	5.218396
K00245	frdB	ortholog	0	1.120126
K00370	narG	ortholog	0	-3.83767
K00370	narG	ortholog	0	-3.83767
K00371	narH	ortholog	-3.0195	-3.77185
K00371	narH	ortholog	-3.0195	-3.77185
K00373	narJ	ortholog	-4.17323	-3.23095
K00373	narJ	ortholog	-4.17323	-3.23095
K00374	narI	ortholog	-4.72475	-3.20749
K00374	narI	ortholog	-4.72475	-3.20749
K00990	NA	ortholog	0	-1.19319
K01077	NA	ortholog	0	1.823924
K01077	NA	ortholog	0	1.823924
K01546	NA	ortholog	0	-2.99733
K01547	NA	ortholog	0	-2.76471
K01548	NA	ortholog	0	-2.68725
K01915	NA	ortholog	0	-1.68281
K07636	phoR	ortholog	0	1.184281
K07639	rstB	ortholog	0	-0.96957
K07644	cusS	ortholog	0	-1.95267
K07657	phoB	ortholog	0	1.436191
K07673	narX	ortholog	0	-1.62945
K07679	evgS	ortholog	0	-1.50811
K07684	narL	ortholog	0	-1.87468
K07687	rcsB	ortholog	0	0.828397
K07700	dpiB	ortholog	0	1.109582
K07702	dpiA	ortholog	0	1.70362
K07708	glnL	ortholog	0	-1.45435
K07709	zraS	ortholog	0	-2.18901
K07712	glnG	ortholog	0	-1.18655
K07806	arnB	ortholog	0	1.159767
K08348	fdnG	ortholog	-3.49741	-4.59413

K08350	fdnI	ortholog	-4.71559	-5.84957
K09474	phoN	ortholog	0	1.912999
K09475	ompC	ortholog	0	-1.96918
K10001	gltI	ortholog	0	4.905064
K10002	gltK	ortholog	0	3.193442
K10004	gltL	ortholog	0	3.177999
K11103	NA	ortholog	0	3.696715
K11103	NA	ortholog	0	3.696715
K19611	NA	ortholog	2.309351	2.716038

ko03010 Ribosome			logFC	logFC
all.mapped	all.mappec	type	BR21_2h	BR21_8h
K02863	NA	ortholog	0	-1.35383
K02864	NA	ortholog	0	-1.23678
K02867	NA	ortholog	0	-1.55841
K02871	NA	ortholog	0	-1.19324
K02876	NA	ortholog	0	-1.30244
K02878	NA	ortholog	0	-1.62668
K02879	NA	ortholog	0	-1.29986
K02881	NA	ortholog	0	-1.457
K02884	NA	ortholog	0	-1.26276
K02886	NA	ortholog	0	-1.31292
K02887	NA	ortholog	-1.33299	-1.22686
K02890	NA	ortholog	0	-1.36872
K02892	NA	ortholog	0	-1.63607
K02895	NA	ortholog	0	-1.42844
K02902	NA	ortholog	0	-1.02687
K02906	NA	ortholog	0	-1.6212
K02907	NA	ortholog	0	-1.40118
K02909	NA	ortholog	0	-1.3848
K02913	NA	ortholog	0	-1.26932
K02914	NA	ortholog	0	-1.09276
K02926	NA	ortholog	0	-1.28923
K02931	NA	ortholog	0	-1.20961

K02933	NA	ortholog	0	-1.46308
K02935	NA	ortholog	0	-1.9964
K02946	NA	ortholog	0	-1.36444
K02956	NA	ortholog	0	-1.11325
K02965	NA	ortholog	0	-1.22578
K02967	NA	ortholog	0	-1.21102
K02968	NA	ortholog	0	-1.62282
K02982	NA	ortholog	0	-1.24032
K02988	NA	ortholog	0	-1.43383
K02990	NA	ortholog	0	-1.29113
K02992	NA	ortholog	0	-0.90355
K02994	NA	ortholog	0	-1.25582
K02996	NA	ortholog	0	-1.15761

Table S4. Differentially expressed genes induced by stress regulons as a response to lethal imipenem exposure of heteroresistant KPC-producing *K. pneumoniae* study strains.

GENE	SEED CATEGORY	SEED SUBCATEGC	count	RpoS	CRP	Fur	CpxR	rcs	other	osmo-ind**	BR7-2h-up	BR7-2h-down	BR21-2h-up	BR21-2h-down	BR7-8h-up	BR7-8h-down	BR21-8h-up	BR21-8h-down
Arginine N-succinyltransferase (EC 2.3.1.109); AstA	Amino Acids and Derivati	Arginine; urea cyc	1	1							1	0	1	0	1	0	1	0
Succinylornithine transaminase (EC 2.6.1.81); AstC	Amino Acids and Derivati	Arginine; urea cyc	1	1							1	0	1	0	1	0	1	0
Arginine ABC transporter, ATP-binding protein ArtP; up B21 8h only	Amino Acids and Derivati	Arginine; urea cyc	1	1						1						0	1	
Arginine ABC transporter, periplasmic arginine-binding protein ArtJ	Amino Acids and Derivati	Arginine; urea cyc	1	1							0	0	0	0	0	0	1	0
gamma-aminobutyrate (GABA) permease, GabP; BLAST, uniprot confirr	Amino Acids and Derivati	Arginine; urea cyc	1	1	1						1				1		1	
Putrescine ABC transporter putrescine-binding protein PotF (TC 3.A.1.1	Amino Acids and Derivati	Arginine; urea cyc	1	1						1					1	0	1	
Putrescine aminotransferase (EC 2.6.1.82); Putrescine:2-oxoglutaric aci	Amino Acids and Derivati	Arginine; urea cyc	1	1					putrescine	1					1	0	1	
Gamma-glutamyl-aminobutyraldehyde dehydrogenase (EC 1.2.1.-); pu	Amino Acids and Derivati	Arginine; urea cyc	1	1	1					1	1	0	1	0	1	0	1	0
Gamma-aminobutyrate:alpha-ketoglutarate aminotransferase (EC 2.6.1	Amino Acids and Derivati	Arginine; urea cyc	1	1	1						0	0	0	0	1	0	1	0
4-aminobutyraldehyde dehydrogenase (EC 1.2.1.19), PatD	Amino Acids and Derivati	Arginine; urea cyc	1	1							0	0	0	0	1	0	1	0
Isochorismate synthase (EC 5.4.4.2) of siderophore biosynthesis; entC	Amino Acids and Derivati	Aromatic amino a	1	0	1	1					0	0	1	0	0	1	1	0
Aspartate--ammonia ligase (EC 6.3.1.1); asnA	Amino Acids and Derivati	Glutamine, glutar	1	0			1				0	0	0	1	0	1	0	1
Threonine dehydratase, catabolic (EC 4.3.1.19) TdcB	Amino Acids and Derivati	Lysine, threonine,	1	0	1	1				1						0	1	
Trans-aconitate 2-methyltransferase (EC 2.1.1.144); up B21 8h	Amino Acids and Derivati	Lysine, threonine,	1	1							0	0	0	0	0	0	1	0
Proline/sodium symporter PutP (TC 2.A.21.2.1)	Amino Acids and Derivati	Proline and 4-hyd	1		1						1	0	0	0	1	0	1	0
**SUBTOTAL**			15	11	6	2	1	0		5	5	0	4	1	9	2	14	1
UPF0229 protein YeaH; up B7 B21 8h	Carbohydrates	Carbohydrates - n	1	1						1					1	0	1	
2,5-diketo-D-gluconate reductase A (EC 1.1.1.274) up B7 B21 8h	Carbohydrates	Central carbohydr	1	1						1					1	0	1	
Aconitate hydratase (EC 4.2.1.3); acnA (also listed metab tab)	Carbohydrates	Central carbohydr	1	1							0	0	0	0	1	0	1	0
Fructose-bisphosphate aldolase class I (EC 4.1.2.13) up B7 B21 8h	Carbohydrates	Central carbohydr	1	1						1					1	0	1	
Glucose-6-phosphate isomerase (EC 5.3.1.9)	Carbohydrates	Central carbohydr	1	0				1			0	0	0	0	0	0	0	1
Pyruvate oxidase [ubiquinone, cytochrome] (EC 1.2.2.2); up B21 8h onl	Carbohydrates	Central carbohydr	1	1						1						0	1	
Transcriptional repressor for pyruvate dehydrogenase complex (poxB);	Carbohydrates	Central carbohydr	1	1	1											1	0	1
Alpha,alpha-trehalose-phosphate synthase [UDP-forming] (EC 2.4.1.15	Carbohydrates	Di- and oligosacch	1	1						1						0	1	
cytoplasmic alpha-amylase up B7 B21 8h	Carbohydrates	Di- and oligosacch	1	1											1	0	1	
Maltose/maltodextrin ABC transporter, permease protein MalF	Carbohydrates	Di- and oligosacch	1	0	1				BaeRS		0	0	0	0	0	0	0	1
Maltose/maltodextrin ABC transporter, permease protein MalG	Carbohydrates	Di- and oligosacch	1	0	1				BaeRS		0	0	0	0	1	0	0	1
Trehalase (EC 3.2.1.28); Periplasmic trehalase precursor (EC 3.2.1.28); u	Carbohydrates	Di- and oligosacch	1	1						1					1	0	1	
Trehalose-6-phosphate phosphatase (EC 3.1.3.12); up B21 8h only	Carbohydrates	Di- and oligosacch	1	1						1						0	1	
Alcohol dehydrogenase (EC 1.1.1.1) up B7 B21 8h; caution: 4 copies of	Carbohydrates	Fermentation	1	1						1					1	0	1	
ethanolamine permease; up B21 8H only; tBLASTn confirmed specific g	Carbohydrates	Sugar alcohols	1	1												0	1	
Glycerol-3-phosphate ABC transporter, ATP-binding protein UgpC (TC 3	Carbohydrates	Sugar alcohols	1	1	1				PhoB		1				1		1	
Glycerol-3-phosphate ABC transporter, periplasmic glycerol-3-phosphat	Carbohydrates	Sugar alcohols	1	1	1				PhoB	1	1				1		1	
Glycerol-3-phosphate ABC transporter, permease protein UgpA (TC 3.A	Carbohydrates	Sugar alcohols	1	1	1				PhoB		1				1		1	
Glycerol-3-phosphate ABC transporter, permease protein UgpE (TC 3.A.	Carbohydrates	Sugar alcohols	1	1	1				PhoB		1				1		1	
Glycerol-3-phosphate transporter; GlpT	Carbohydrates	Sugar alcohols	1	0	1						1		1		1		1	
Glycerophosphoryl diester phosphodiesterase, periplasmic; GlpQ (EC 3.	Carbohydrates	Sugar alcohols	1	0	1						1		1		1	0	1	
Mannitol-1-phosphate 5-dehydrogenase (EC 1.1.1.17)	Carbohydrates - stress	Sugar alcohols	1								0	0	0	0	1	0	1	0
Sorbitol-6-phosphate 2-dehydrogenase (EC 1.1.1.140)	Carbohydrates-stress	Monosaccharides	1								0	0	0	0	0	0	1	0
**SUBTOTAL**			23	16	9	0	0	1		9	6	0	2	0	15	1	19	4
Lipoprotein nlpI precursor	Cell Division and Cell Cycl	Cell Division and C	1	1							0	0	0	1	0	0	0	1
Cyclopropane-fatty-acyl-phospholipid synthase (EC 2.1.1.79), plant type	Cell Division and Cell Cycl	Cell Division and C	1	1							0	0	0	1	0	0	0	0
Cell filamentation protein fic; up B21 8h only; tBLASTn found homolog	Cell Division and Cell Cycl	Cell Division and C	1	1						1						0	1	
**SUBTOTAL**			3	3	0	0	0	0		1	0	0	0	2	0	0	1	1
L,D-transpeptidase ErfK linking Lpp to murein; up B7, B21 8h; osmotic s	Cell Wall and Capsule	Cell Wall and Caps	1	1						1					1	0	1	
L,D-transpeptidase YcfS	Cell Wall and Capsule	Cell Wall and Caps	1	0			1				0	0	0	0	0	0	1	0
L,D-transpeptidase YnhG; up B7 B21 8h; cell wall	Cell Wall and Capsule	Cell Wall and Caps	1	1						1					1	0	1	
Lipid A biosynthesis lauroyl acyltransferase (EC 2.3.1.-)	Cell Wall and Capsule	Gram-Negative ce	1	0				1			0	0	0	0	0	1	0	1
Lipoprotein NlpD	Cell Wall and Capsule	Gram-Negative ce	1	1							0	0	0	0	0	0	1	0
Outer membrane chaperone Skp (OmpH) precursor	Cell Wall and Capsule	Gram-Negative ce	1						rpoE		0	0	0	0	0	0	0	1
Uncharacterized protein YhjG; up B7 B21 8h	Cell Wall and Capsule	Gram-Negative ce	1	1						1					1	0	1	
**SUBTOTAL**			7	4	0	0	1	1		3	0	0	0	0	3	1	5	2
DNA repair protein RecN	DNA Metabolism	DNA repair	1								0	0	0	0	0	0	1	0
Cold shock protein CspD	Stress Response	Cold shock	1	0	1						1	0	0	0	1	0	1	0
**SUBTOTAL**			2	0	1	0	0	0	0	0	1	0	0	0	1	0	2	0
Conidiation-specific protein 10; up B21 8h	Dormancy and Sporulatic	Dormancy and Sp	1	1						1						0	1	
FIG004684: SpoVR-like protein; up B7 B21 8h	Dormancy and Sporulatic	Dormancy and Sp	1	1					rpoE	1					1	0	1	
**SUBTOTAL**			2	2	0	0	0	0		2	0	0	0	0	1	0	2	0
Acyl-CoA dehydrogenase (EC 1.3.8.7); up B7 B21 8h; tBLASTn confirmec	Fatty Acids, Lipids, and Is	Fatty acids	1	1											1	0	1	
**SUBTOTAL**			1	1	0	0	0	0		0	0	0	0	0	1	0	1	0
GENE	SEED CATEGORY	SEED SUBCATEGC	count	RpoS	CRP	Fur	CpxR	rcs	other	osmo-ind**	BR7-2h-up	BR7-2h-down	BR21-2h-up	BR21-2h-down	BR7-8h-up	BR7-8h-down	BR21-8h-up	BR21-8h-down
Bacterioferritin; up B7 B21 8h	Iron acquisition and meta	Iron acquisition ar	1	1											1	0	1	
Ferric reductase (1.6.99.14); fhuF	Iron acquisition and meta	Iron acquisition ar	1	0		1			OxyR		0	0	0	0	0	0	1	0
TonB-dependent receptor; Outer membrane receptor for ferric enterob	Iron acquisition and meta	Iron acquisition ar	1	0	1	1					0	0	1	0	0	1	1	0
2,3-dihydro-2,3-dihydroxybenzoate dehydrogenase (EC 1.3.1.28) [enter	Iron acquisition and meta	Siderophores	1	0	1	1					0	0	0	0	0	0	1	0
Enterobactin synthetase component F, serine activating enzyme (EC 2.7	Iron acquisition and meta	Siderophores	1	0	1	1					0	0	1	0	0	1	1	0
Proofreading thioesterase in enterobactin biosynthesis EntH	Iron acquisition and meta	Siderophores	1	0	1	1					0	0	0	0	0	1	1	0

Non-specific DNA-binding protein Dps / Iron-binding ferritin-like antioxi	Iron acquisition and meta	Iron acquisition a	1	1					OxyR	1	0	0	0	0	1	0	1	0
**SUBTOTAL**			7	2	4	5	0	0		1	0	0	2	0	2	3	7	0
ABC transporter ATP-binding protein (Tn5 mutant)	Membrane Transport	ABC transporters	1								0	0	0	0	0	0	1	0
Glutamine ABC transporter, periplasmic glutamine-binding protein (TC	Membrane Transport	ABC transporters	1								0	0	0	0	1	0	1	0
cation transport regulator chaB; up B7 B21 8h	Membrane Transport	Cation transporte	1	1						1					1	0	1	
Outer membrane protein X precursor	Membrane Transport	Cation transporte	1	1			1				0	0	0	0	0	0	0	1
Putative PerM family permease; up B21 8h only	Membrane Transport	Membrane Trans	1	1						1						0	1	
Transcriptional activator NhaR	Membrane Transport	Uni- Sym- and Ant	1	0				1			0	0	0	0	0	0	0	1
**SUBTOTAL**			6	3	0	0	1	1		2	0	0	0	0	2	0	4	2
Nitrate/nitrite transporter NarK; up B7 B21 8h; tBLASTn 75% homology	Nitrogen Metabolism	Denitrification	1	1											1	0	1	
Respiratory nitrate reductase alpha chain, narG (EC 1.7.99.4)	Nitrogen Metabolism	Nitrogen Metabol	1	0			1		Fnr		0	1	0	0	0	1	0	1
Respiratory nitrate reductase beta chain, narY(EC 1.7.99.4); down all sa	Nitrogen Metabolism	Nitrogen Metabol	1	1						1		1	0	1	0	1	0	1
**SUBTOTAL**			3	2	0	0	1	0		1	0	2	0	1	1	2	1	2
ATP-dependent Clp protease ATP-binding subunit ClpA	Protein Metabolism	Protein degradati	1								0	0	0	0	1	0	0	0
FKBP-type peptidyl-prolyl cis-trans isomerase FkpA precursor (EC 5.2.1.	Protein Metabolism	Protein folding	1						RpoE									1
Acidic protein msyB; up B7 B21 8h	Protein Metabolism	Protein folding	1	1											1	0	1	
Chaperone protein DnaJ	Protein Metabolism	Protein folding	1	0				1			0	0	0	0	0	0	0	1
Chaperone protein DnaK	Protein Metabolism	Protein folding	1	0				1			0	0	0	0	0	0	0	1
MicA, non-coding RNA	Protein Metabolism	Protein folding	1						RpoE						1	0	1	
Chaperone protein HtpG	Protein Metabolism	Protein folding	1	0					RpoH		0	0	0	0	0	0	0	1
**SUBTOTAL**			7	1	0	0	0	2		0	0	0	0	0	3	0	2	4
Biofilm PGA outer membrane secretin PgaA	Regulation and Cell signa	Quorum sensing a	1	0				1			0	0	0	0	0	0	1	0
DNA-binding capsular synthesis response regulator RcsB	Regulation and Cell signa	Regulation and Ce	1	0				1			0	0	0	0	0	0	1	0
**SUBTOTAL**			2	0	0	0	0	2		0	0	0	0	0	0	0	2	0
Electron transport complex protein RnfA	Respiration	Electron donating	1	0			1				0	0	0	0	0	1	0	0
Succinate-semialdehyde dehydrogenase [NAD] (EC 1.2.1.24); Succinate-	Respiration	Electron donating	1	1												0	1	
**SUBTOTAL**			2	1	0	0	1	0		0	0	0	0	0	0	1	1	0
micL, non coding RNA (Lipid A precursor)	RNA Metabolism	RNA processing a	1						RpoE								1	
RNA polymerase sigma factor RpoS	RNA Metabolism	Transcription	1	1							0	0	0	0	1	0	1	0
Iron-sulfur cluster assembly protein SufB	RNA Metabolism	RNA processing a	1	1		1			OxyR		0	0	0	0	0	0	1	0
**SUBTOTAL**			3	2	0	1	0	0	0	0	0	0	0	0	1	0	3	0
Choline-glycine betaine transporter	Stress Response	Osmotic stress	1	0							0	0	0	0	1	0	1	0
DedA family inner membrane protein YdjX	Stress Response	Osmotic stress	1	0							0	0	0	0	0	1	0	1
DedA family inner membrane protein YghB	Stress Response	Osmotic stress	1	0							0	0	0	0	0	0	0	1
Entericidin B precursor; entB	Stress Response	Osmotic stress	1	0	1	1					0	0	0	0	0	0	1	0
General stress protein 18	Stress Response	Osmotic stress	1	1							0	0	0	0	1	0	1	0
Glutathione S-transferase, omega (EC 2.5.1.18)	Stress Response	Osmotic stress	1	1							0	0	0	0	1	0	1	0
Glutathione S-transferase, theta (EC 2.5.1.18)	Stress Response	Osmotic stress	1	1							0	0	0	0	1	0	1	0
Hnr protein; response regulator, protein turnover, controls stability of r	Stress Response	Osmotic stress	1	1							0	0	0	0	0	0	1	0
L-proline glycine betaine ABC transport system permease protein ProV (	Stress Response	Osmotic stress	1	0							0	0	0	0	0	0	1	0
L-proline glycine betaine binding ABC transporter protein ProX (TC 3.A.1	Stress Response	Osmotic stress	1	0							0	0	0	0	0	0	1	0
Osmoprotectant ABC transporter ATP-binding subunit YehX	Stress Response	Osmotic stress	1	0							0	0	0	0	0	0	1	0
Osmoprotectant ABC transporter binding protein YehZ	Stress Response	Osmotic stress	1	0							0	0	0	0	1	0	1	0
Osmoprotectant ABC transporter inner membrane protein YehW	Stress Response	Osmotic stress	1	0							0	0	0	0	0	0	1	0
Osmoprotectant ABC transporter permease protein YehY	Stress Response	Osmotic stress	1	0							0	0	0	0	0	0	1	0
Osmotically inducible lipoprotein B precursor: OsmB	Stress Response	Osmotic stress	1	1			1	1			0	0	0	0	0	0	1	0
Osmotically inducible lipoprotein E precursor; OsmE	Stress Response	Osmotic stress	1	1							0	0	0	0	1	0	1	0
Osmotically inducible protein C OsmC	Stress Response	Osmotic stress	1	1	1			1			0	0	0	0	1	0	1	0
Osmotically inducible protein OsmY	Stress Response	Osmotic stress	1	1	1						0	0	0	0	1	0	1	0
Outer membrane lipoprotein blc precursor	Stress Response	Osmotic stress	1	1							0	0	0	0	1	0	1	0
Phosphate starvation-inducible protein PsiF	Stress Response	Osmotic stress	1	1							0	0	0	0	1	0	1	0
Protein involved in stability of MscS mechanosensitive channel	Stress Response	Osmotic stress	1	0							0	0	0	0	0	0	0	1
Universal stress protein B; cell death in stat phase when overexpressed,	Stress Response	Osmotic stress	1	1	1					1	0	0	0	0	1	0	1	0
COG1683: Uncharacterized conserved protein / FIG143828: Hypothetic	Uncharacterized	Osmotic stress	1	1										1				
FIG00638451: hypothetical protein yiaG putative transcriptional regulat	Uncharacterized	Osmotic stress	1	1											1	0	1	

GENE	SEED CATEGORY	SEED SUBCATEGC	count	RpoS	CRP	Fur	CpxR	rcs	other	osmo-ind**	BR7-2h-up	BR7-2h-down	BR21-2h-up	BR21-2h-down	BR7-8h-up	BR7-8h-down	BR21-8h-up	BR21-8h-down
Inner membrane protein YphA; up B21 8h	Uncharacterized	Osmotic stress	1	1													1	
Probable secreted protein; up B7 B21 8h	Uncharacterized	Osmotic stress	1	1											1	0	1	
Protein YaiA; up B21 8h only	Uncharacterized	Osmotic stress	1	1												0	1	
Protein YciF; up B21 8h only	Uncharacterized	Osmotic stress	1	1												0	1	
Putative oxidoreductase YncB; up B21 8h only	Uncharacterized	Osmotic stress	1	1												0	1	
UPF0028 protein YchK aka rssA; up B21 8h only; annot as misc. uniprot	Uncharacterized	Osmotic stress	1	1												0	1	
Dipeptide transport ATP-binding protein DppF (TC 3.A.1.5.2)	Stress Response	Osmotic stress	1	0		1					0	0	0	0	0	0	1	0
Dipeptide transport system permease protein DppB (TC 3.A.1.5.2)	Stress Response	Osmotic stress	1	0		1					0	0	0	0	1	0	1	0
Dipeptide transport system permease protein DppC (TC 3.A.1.5.2)	Stress Response	Osmotic stress	1	0		1					0	0	0	0	0	0	1	0
Dipeptide-binding ABC transporter, periplasmic substrate-binding comp	Stress Response	Osmotic stress	1	0		1					0	0	0	0	0	0	1	0
**SUBTOTAL**			34	19	4	5	1	2		1	0	0	0	1	14	1	30	3
Catalase (EC 1.11.1.6)	Stress Response	Oxidative stress	1	1							0	0	0	0	1	0	1	0
Cell wall endopeptidase, family M23/M37	Stress Response	Oxidative stress	1	0							0	0	0	0	0	0	1	0
Gamma-glutamyltranspeptidase (EC 2.3.2.2)	Stress Response	Oxidative stress	1	0							0	0	0	0	1	0	1	0

Glutathione peroxidase family protein	Stress Response	Oxidative stress	1	0							0	0	0	0	0	0	1	0
Glutathione reductase (EC 1.8.1.7)	Stress Response	Oxidative stress	1	0							0	0	0	0	0	0	0	1
Glutathione S-transferase (EC 2.5.1.18)	Stress Response	Oxidative stress	1	0							0	0	0	0	0	0	1	0
Manganese catalase (EC 1.11.1.6)	Stress Response	Oxidative stress	1	0							0	0	0	0	0	0	1	0
Non-heme chloroperoxidase (EC 1.11.1.10)	Stress Response	Oxidative stress	1	0							0	0	0	0	1	0	1	0
Probable glutathione S-transferase (EC 2.5.1.18), YfcG homolog	Stress Response	Oxidative stress	1	0							0	0	0	0	0	0	1	0
Regulatory protein SoxS	Stress Response	Oxidative stress	1	0							0	0	1	0	0	0	0	0
Regulatory protein soxS	Stress Response	Oxidative stress	1	0							0	1	0	1	0	1	0	1
Senescence marker protein-30	Stress Response	Oxidative stress	1	0							0	0	0	0	0	0	1	0
Superoxide dismutase [Cu-Zn] precursor (EC 1.15.1.1)	Stress Response	Oxidative stress	1	0							0	0	0	0	0	0	1	0
Superoxide dismutase [Fe] (EC 1.15.1.1)	Stress Response	Oxidative stress	1	0							0	0	0	0	0	0	1	0
Thioredoxin 2 (EC 1.8.1.8); TrxC	Stress Response	Oxidative stress	1	0	1				OxyR		1	0	0	0	1	0	1	0
**SUBTOTAL**			15	1	1	0	0	0		0	1	1	1	1	4	1	12	2
L,D-transpeptidase YcfS	Stress Response	Peptidoglycan dar	1	0							0	0	0	0	0	0	1	0
**SUBTOTAL**			1	0	0	0	0	0		0	0	0	0	0	0	0	1	0
UPF0337 protein - YjbJ-unchar general stress protein	Stress Response	Stress Response	1	1				1		1	0	0	0	0	1	0	1	0
Carbon starvation induced protein CsiD	Stress Response	Stress Response -	1	1	1					1	1	0	0	0	1	0	1	0
FOG: GGDEF domain	Stress Response	Stress Response -	1	0							0	0	0	0	0	0	1	0
GGDEF family protein	Stress Response	Stress Response -	1	0							0	0	0	0	0	0	1	0
Membrane-bound metal-dependent hydrolase YdjM, induced during SC	Stress Response	Stress Response -	1	0							0	0	0	0	0	1	0	0
Nitrogen regulation protein NtrB (EC 2.7.13.3) (aka glnL)	Stress Response	Stress Response -	1	0							0	0	0	0	0	1	0	1
Nitrogen regulation protein NtrC (aka glnG)	Stress Response	Stress Response -	1	0							0	0	0	0	0	0	0	1
NtrC family Transcriptional regulator, ATPase domain	Stress Response	Stress Response -	1	0							0	0	0	0	1	0	0	0
Probable protease HtpX (EC 3.4.24.-)	Stress Response	Stress Response -	1	0			1				0	0	0	0	0	0	1	0
protein iraP	Stress Response	Stress Response -	1	1							0	0	0	0	1	0	1	0
Protein sprT	Stress Response	Stress Response -	1	0							0	0	0	0	0	1	0	1
Putative ThuR, regulatory protein for trehalosemaltose transport	Stress Response	Stress Response -	1	0							0	0	0	0	0	0	0	1
Rtn protein	Stress Response	Stress Response -	1	0							0	0	0	0	0	0	1	0
Sensory box/GGDEF family protein	Stress Response	Stress Response -	1	0							0	0	0	0	0	0	1	0
Starvation lipoprotein Slp paralog	Stress Response	Stress Response -	1	0							0	0	0	0	0	0	0	1
Stationary phase inducible protein CsiE	Stress Response	Stress Response -	1	0							0	0	0	0	1	0	1	0
Transcriptional activator RamA	Stress Response	Stress Response -	1	0							0	0	0	0	0	0	1	0
Transcriptional regulator, IclR family	Stress Response	Stress Response -	1	0	1						1	0	0	0	1	0	1	0
Uncharacterized protein YqjB	Stress Response	Stress Response -	1	0							0	0	0	0	0	1	0	1
Universal stress protein F	Stress Response	Stress Response -	1	0							0	0	0	0	0	0	1	0
UPF0337 protein - YjbJ-unchar general stress protein	Stress Response	Stress Response -	1	1				1			0	0	0	0	1	0	1	0
**SUBTOTAL**			21	4	2	0	1	2		2	2	0	0	0	7	4	13	6
Cold shock protein CspA	Stress Response	Cold shock	1	0							0	0	0	1	0	1	0	1
FIG005189: putative transferase clustered with tellurite resistance prot	Stress Response	Detoxification	1	0							0	0	0	0	1	0	1	0
TsgA protein homolog	Stress Response	Detoxification	1	0							0	0	0	0	0	0	0	1
Uncharacterized membrane lipoprotein clustered with tellurite resistan	Stress Response	Detoxification	1	0							0	0	0	0	0	0	1	0
Heat shock protein hslJ	Stress Response	Heat shock	1	0					RpoH		0	0	0	0	0	1	0	1
Small heat shock protein-IbpA-like holding chaperone (protein BLAST)	Stress Response	Heat shock	1	0							0	0	0	0	1	0	1	0
**SUBTOTAL**			6	0	0	0	0	0		0	0	0	0	1	2	2	3	3
FIG004088: inner membrane protein YebE	Uncharacterized	Uncharacterized	1	0			1				0	0	0	0	0	1	0	0
FIG00731387: hypothetical protein-YgaU	Uncharacterized	Uncharacterized	1	1							0	0	0	0	1	0	1	0
FIG00731925: hypothetical protein-Two-component-system connector	Uncharacterized	Uncharacterized	1	1							0	0	0	0	0	0	1	0
GENE	SEED CATEGORY	SEED SUBCATEGC	count	RpoS	CRP	Fur	CpxR	rcs	other	osmo-ind**	BR7-2h-up	BR7-2h-down	BR21-2h-up	BR21-2h-down	BR7-8h-up	BR7-8h-down	BR21-8h-up	BR21-8h-down
FIG00732338: hypothetical protein - YgaM	Uncharacterized	Uncharacterized	1	1			1				0	0	0	0	1	0	1	0
FIG01219827: hypothetical protein - ygaC	Uncharacterized	Uncharacterized	1	0		1		1			0	0	0	0	1	0	1	0
probable lipoprotein- ybjP	Uncharacterized	Uncharacterized	1	1							0	0	0	0	0	0	1	0
**SUBTOTAL**			6	4	0	1	2	1		0	0	0	0	0	3	1	5	0
SbmA protein	Virulence, Disease and D	Resistance to anti	1	0					RpoE		0	0	0	0	0	0	0	1
Glyoxalase family protein	Virulence, Disease and D	Resistance to anti	1								1	0	0	0	1	0	1	0
**SUBTOTAL**			2	0	0	0	0	0		0	1	0	0	0	1	0	1	1

**Summary**		count	**RpoS**	**CRP**	**Fur**	**CpxR**	**rcs**	**other**	**osmo-ind****	BR7-2h-up	BR7-2h-down	BR21-2h-up	BR21-2h-down	BR7-8h-up	BR7-8h-down	BR21-8h-up	BR21-8h-down
	Amino Acids and Derivatives	15	11	6	2	1	0		5	4	0	4	1	9	2	14	1
	Carbohydrates	23	16	9	0	0	1	BaeRS, Pho	9	6	0	2	0	15	1	19	4
	Cell Division and Cell Cycle	3	3	0	0	0	0		1	0	0	0	2	0	0	1	1
	Cell Wall and Capsule	7	4	0	0	1	1	RpoE	3	0	0	0	0	3	1	5	2
	DNA Metabolism	2	0	1	0	0	0		0	1	0	0	0	1	0	2	0
	Dormancy and Sporulation	2	2	0	0	0	0	RpoE	2	0	0	0	0	1	0	2	0
	Fatty Acids, Lipids, and Isoprenoids	1	1	0	0	0	0		0	0	0	0	0	1	0	1	0
	Iron acquisition and metabolism	7	2	4	5	0	0		1	0	0	2	0	2	3	7	0
	Membrane Transport	6	3	0	0	1	1		2	0	0	0	0	2	0	4	2
	Nitrogen Metabolism	3	2	0	0	1	0	Fnr	1	0	2	0	1	1	2	1	2
	Protein Metabolism	7	1	0	0	0	2	RpoE, RpoH	0	0	0	0	0	3	0	2	4
	Regulation and Cell signaling	2	0	0	0	0	2		0	0	0	0	0	0	0	2	0

Respiration	2	1	0	0	1	0		0	0	0	0	0	0	1	1	0
RNA Metabolism	3	2	0	1	0	0		0	0	0	0	0	1	0	3	0
Osmotic stress	34	19	4	5	1	2		34	0	0	0	1	14	1	30	3
Oxidative stress	15	1	1	0	0	0		0	1	1	1	1	4	1	12	2
Peptidoglycan damage	1	0	0	0	0	0		0	0	0	0	0	0	0	1	0
Cold shock, Heat Shock, Detoxification	6	0	0	0	0	0		0	0	0	0	1	2	2	3	3
Virulence, Disease and Defense	2	0	0	0	0	0	RpoE	0	1	0	0	0	1	0	1	1
Stress Response-General	21	4	2	0	1	2		2	2	0	0	0	7	4	13	6
Uncharacterized	6	4	0	1	2	1		0	0	0	0	0	3	1	5	0
Total	168	76	27	14	9	12		60	15	3	9	7	70	19	129	31

Weber H, Polen T, Heuveling J, Wendisch VF, Hengge R. 2005. Genome-wide analysis of the general stress response network in Escherichia coli: sigmaS-dependent genes, promoters, and sigma factor selectivity. J Bacteriol **187:**1591-160[illegible]
